# Ratoon Season Rice Reduces Methane Emissions by Limiting Acetic Acid Transport to the Rhizosphere and Inhibiting Methanogens

**DOI:** 10.1002/advs.202507916

**Published:** 2025-11-26

**Authors:** Jingnan Zou, Hailong Xu, Bin Qin, Chaojie Lan, Jinying Li, Bianhong Zhang, Huabin Zhang, Chunlin Guo, Hongfei Chen, Zhongming Fang, Quanzhi Zhao, Wenfei Wang, Changxun Fang, Zhixing Zhang, Wenxiong Lin

**Affiliations:** ^1^ Fujian Provincial Key Laboratory of Agroecological Processing and Safety Monitoring Fujian Agriculture and Forestry University College of Jun Cao Science and Ecology Fuzhou 350002 P. R. China; ^2^ Key Laboratory of Crop Ecology and Molecular Physiology (Fujian Agriculture and Forestry University) Fujian Province University College of Jun Cao Science and Ecology Fuzhou 350002 P. R. China; ^3^ College of Agriculture Fujian Agricultural and Forestry University Fuzhou 350002 China; ^4^ Agricultural Ecology Research Institute School of Life Sciences Fujian Agriculture and Forestry University Fuzhou 350002 China; ^5^ Institute of Rice Industry Technology Research/Key Laboratory of Functional Agriculture Guizhou Provincial Department of Education/Key Laboratory of Plant Resource Conservation and Germplasm Innovation in Mountainous Region Ministry of Education/Key Laboratory of Molecular Breeding for Grain and Oil Crops in Guizhou Province/College of Agricultural Sciences Guizhou University Guiyang 550025 China; ^6^ College of Tropical Crops Hainan University Haikou Hainan 570228 China

**Keywords:** distribution of photosynthate, methane emissions, methanogenic bacteria, ratoon season rice, yield

## Abstract

Rice paddies are a major, persistent source of atmospheric methane (CH_4_), emission rates depend on the partitioning of photosynthate carbon between the rice plant and the rhizosphere microbiome. Although ratoon season rice (RR) is shown to emit far less CH_4_ than main‐crop rice (MC), the mechanisms have remained unresolved. This work conducts a 2‐year field experiment in which RR is compared with MC and with late rice (LR) synchronized to the RR heading stage. Relative to MC and LR, RR lowers daily CH_4_ flux by 91%, raises daily grain yield by 34%–57%, and increases net economic return by 90%–136%. Mechanistically, ^13^C‐labelling reveals that RR diverted more newly fixed carbon to the grain and less to the rhizosphere, thereby restricting acetate availability for methanogens. Rhizosphere metagenomics show reduced abundance of Methanobacteriaceae and down‐regulation of methanogenic genes in RR. This carbon‐reallocation pattern is underpinned by an abscisic acid (ABA)‐mediated interaction between OsCIPK2 and OsSWEET1A, which simultaneously curtailed carbon efflux from roots and enhanced grain filling. This study is the first to establish a comprehensive framework of “ABA regulation—carbon allocation—microbial function—emission reduction and efficiency enhancement.” It provides targetable strategies for carbon allocation and microbial management within climate‐smart rice farming systems.

## Graphical abstract

This study finds that the interaction between ABA‐OsCIPK2‐OsSWEET1A reduces the allocation of methane producing bacteria carbon source (acetic acid) content to the rhizosphere soil of ratoon season rice, thereby reducing methane emissions.

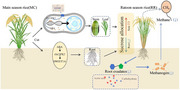

## 1 Introduction

As the primary calorie source for over 3.5 billion people, rice cultivation spans 180 million hectares globally yet faces a dual challenge: but it must reconcile a 30% production surge by 2050 with a sizeable environmental footprint. however, intensify greenhouse‐gas emissions, undermining climate stability and human health. Paddy fields releasing 24 million tons of methane annually – a potent greenhouse gas with 28–34 times the global warming potential of CO_2_ over a century.^[^ ^1^, ^2^, ^3^ ^]^ This creates a critical dilemma: how to raise yields without further methane release on shrinking arable land.^[^ ^3^ ^]^ Classic water‐ and fertilizer‐based mitigation curtail emissions by only 15%–30% and often incur 5% yield losses.^[^ ^4^ ^]^ Recent research suggests that changes in rice cultivation patterns could be an effective strategy to achieve both high rice yields and reduced methane emissions, with the ratoon rice cultivation system being a prime example.^[^ ^5^, ^6^ ^]^

Ratoon season rice (RR) regenerates from basal axillary buds after the main‐crop (MC) harvest, producing a second grain crop within 70–90 days‐≈60% of the MC growth period‐without the need for re‐transplanting, land preparation, or seedling raising. Consequently, its yield per unit time is more than 30% higher than that of the main crop.^[^ ^7^ ^]^ This system is now promoted in countries including India, Japan, the Philippines, Brazil, the USA, and China.^[^ ^8^ ^]^ In China alone, the suitable area for RR is estimated at 5.6 million hectares and is rapidly expanding.^[^ ^6^ ^]^ Numerous studies have consistently shown that CH_4_ emissions from RR are over 70% lower than those from both MC and late rice (LR, synchronized in heading time with RR), while N_2_O emissions remain comparable.^[^ ^7^, ^9^, ^10^, ^11^, ^12^, ^13^ ^]^ The fate of photosynthate is a key determinant of both grain yield and CH_4_ flux. It is estimated that 15%–30% of newly fixed carbon leaks into the rhizosphere, fueling up to 60% of methanogenic activity in paddies. Among these carbon substrates, acetic acid alone accounts for about two‐thirds of total CH_4_ production.^[^ ^14^, ^15^ ^]^ Therefore, redirecting assimilates toward grains and away from rhizodeposition presents a direct path to simultaneously increase yield and reduce methane emissions, a principle demonstrated by the transgenic introduction of barley SUSIBA2 or the knockout of OsGS3 in rice.^[^ ^4^, ^16^ ^]^ Our previous work confirmed that RR inherently employs this strategy, allocating more photosynthate to grains and less to the rhizosphere, thereby resulting in lower methane emissions.^[^ ^7^, ^9^ ^]^ This reduction is particularly relevant to the acetoclastic methanogenesis pathway, which is primarily carried out by methanogens from the genera Methanosarcina and Methanosaeta.

Stable‐isotope probing (SIP) and DNA‐SIP now allow real‐time tracking of photoassimilated ^13^C from plant organs to rhizosphere microbes and trace‐gas fluxes. After pulse‐labelling, ^13^C‐labeled substrates exuded by roots are assimilated by active microorganisms; their ^13^C‐DNA is then separated by density‐gradient ultracentrifugation, directly linking microbial identity to metabolic function. Although 10%–30% of rice photosynthates leak into the rhizosphere and fuel methanogens,^[^ ^17^, ^18^ ^]^ we still lack a systematic view of how this carbon specifically enriches methanogenic archaea and drives CH_4_ formation. Qian et al.^[^ ^19^ ^]^ used paddy soil metagenomics to identify competitive interactions between methanogen functional genes (e.g., mcrA) and sulfate‐reducing bacteria, while also revealing their regulatory role in methane production. Nevertheless, existing studies still lack a systematic understanding of the dynamic response mechanisms of methanogenic microbial functions and gene networks under ^13^C tracing. Therefore, there is an urgent need for cross‐scale research that integrates in situ metagenomics with stable isotope probing (DNA‐SIP) techniques.

**Figure 1:**
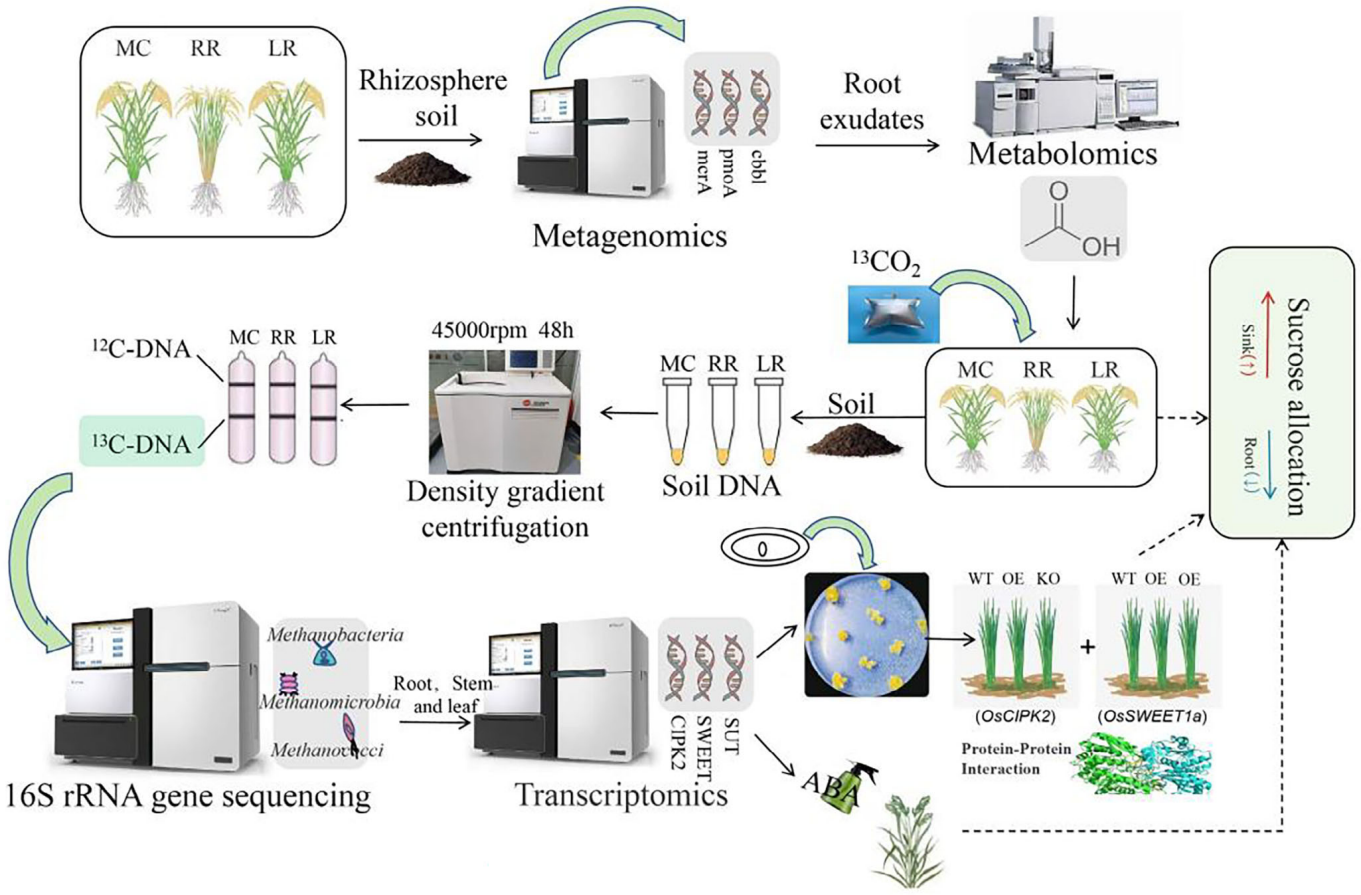
Schematic workflow of the multi‐omics experiment. Three rice‐culture systems‐main‐crop rice (MC), ratoon season rice (RR), and late‐season rice (LR)‐were established with synchronized heading dates between RR and LR. MC, LR and RR rhizosphere soils were profiled by metagenomics/metabolomics; ^13^CO_2_‐labeled root exudates were tracked by DNA‐SIP to identify active microbes; metatranscriptomics highlighted photosynthate‐allocation genes; By studying the ABA induced OsCIPK2‐OsSWEET1A interaction, we aim to explore the mechanisms of carbon allocation and CH_4_ emissions.

Our previous research found that the photosynthate allocation pattern in ratoon season rice (RR) is significantly associated with elevated abscisic acid (ABA) levels during the late grain‐filling stage.^[^ ^7^ ^]^ This hormone speeds assimilate flow, especially to inferior spikelets, thereby raising yield.^[^ ^20^ ^]^ The same effect can be achieved by spraying ABA or knocking out OsABA8ox2.^[^ ^21^ ^]^ ABA‐triggered CIPKs can then phosphorylate sucrose transporters, as exemplified by the MdCIPK23→MdSUT2.2 module in apple.^[^ ^22^, ^23^, ^24^ ^]^ Conversely, rice mutants for OsSUT1, OsSWEET11, and OsSWEET14 retain assimilates in stems and reduce sucrose transport to grains, thus lowering yield.^[^ ^25^, ^26^, ^27^ ^]^ This presents a compelling hypothesis: could ABA‐mediated signaling coordinate RR's carbon reallocation through OsCIPK2‐OsSWEET1A molecular cascades, simultaneously optimizing yield and suppressing methanogenesis? We therefore hypothesize that in RR, enhanced ABA signaling promotes OsCIPK2 expression and the OsCIPK2‐OsSWEET1A interaction, leading to increased ^13^C import into grains and reduced acetate in root exudates. The consequent scarcity of substrates would then suppress methanogen activity and lower CH_4_ flux. Thus, we propose that ABA signalling acts as a master regulator that redistributes photosynthate toward the grain and away from the rhizosphere, simultaneously boosting yield and curbing CH_4_ emissions (**Figure** **1**).

## 2 Results

### 2.1 Ratoon Season Rice Simultaneously Reduces Methane Emissions and Enhances Economic Benefit

**Figure 2:**
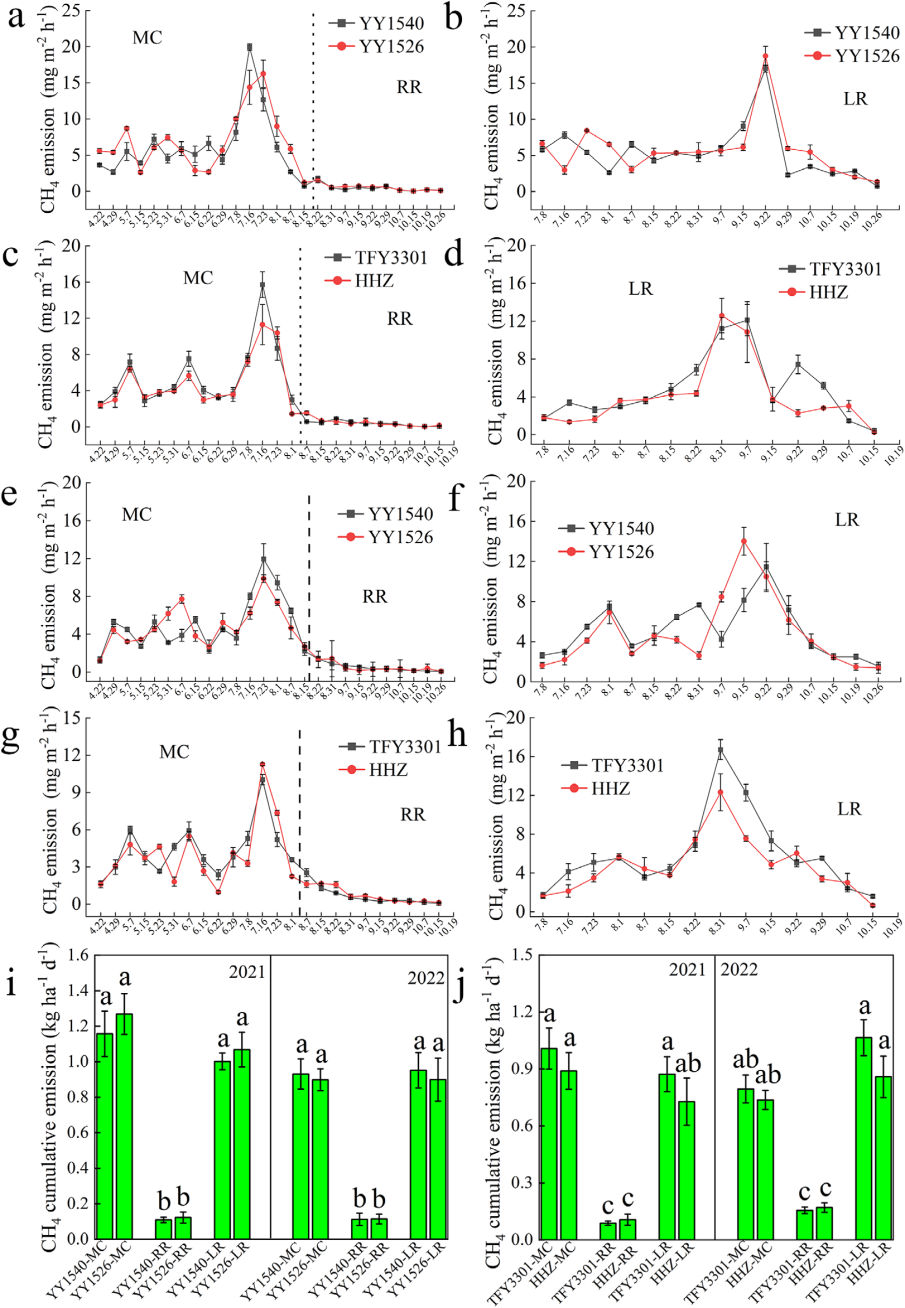
Variations in CH_4_ emission flux and daily average emission under different rice cultivation modes for different varieties of MC, RR and LR in 2021 and 2022. a) CH_4_ emission flux for YY1540 and YY1526 MC and RR in 2021. b) CH_4_ emission flux for YY1540 and YY1526 LR in 2021. c) CH_4_ emission flux for TFY3301 and HHZ MC and RR in 2021. d) CH_4_ emission flux for TFY3301 and HHZ LR in 2021. e) CH_4_ emission flux for YY1540 and YY1526 MC and RR in 2022. f) CH_4_ emission flux for YY1540 and YY1526 LR in 2022. g) CH_4_ emission flux for TFY3301 and HHZ MC and RR in 2022. h) CH_4_ emission flux for TFY3301 and HHZ LR in 2022. i) Daily average CH_4_ emission for YY1540 and YY1526 MC, RR, and LR. j) Daily average CH_4_ emission for TFY3301 and HHZ MC, RR and LR. All data are presented as Means ± SD (n = 3 biological replicates), in (i) and (j) the different lowercase letters above the column graphs indicate significant differences by Duncan's test (*p* < 0.05). All figures were plotted using Origin 2021 software. Source data are provided in the source data file.

During a 2‐year field experiment in southeast China involving four rice varieties cultivated under main‐crop (MC), ratoon season rice (RR), and late rice (LR) regimes, the daily average methane flux from RR was consistently and significantly lower than from MC and LR. The emission peaks for MC and LR occurred during the grain‐filling stage, in contrast to RR, where fluxes peaked at the tillering stage (**Figure** **2**). When calculated as cumulative flux divided by growth duration, the daily CH_4_ emission from RR was 77%–99% lower than that from both MC and LR (Figure 2i,j), while the daily grain yield increased by 25%–36% (Tables S3 and S4, Supporting Information). To assess the generalizability of these findings, a complementary experiment was conducted in a tropical region. Consistent with the results from the subtropical site, RR in the tropical region also demonstrated a substantial reduction in daily CH_4_ emissions (78.45%–91.42%) and an increase in daily yield (24.01%–33.31%) compared to MC and LR (Figure S4; Table S5, Supporting Information).

Among the different varieties, the hybrid YY1540 showed the highest absolute CH_4_ emissions and yield, whereas the conventional variety HHZ showed the lowest. In contrast to CH_4_, daily N_2_O fluxes did not differ significantly among the three cultivation regimes. Emissions of N_2_O were transient, spiking only shortly after fertilization events, underscoring its direct link to nitrogen fertilizer application (Figures S5–S7, Supporting Information). Owing to the reduced requirement for agricultural inputs and practices such as seed sowing, seedling raising, transplanting, and land preparation, the RR system exhibited lower carbon and nitrogen footprints and higher resource use efficiency (Figures S8 and S9, Supporting Information). Consequently, the daily net economic return from RR increased by 90.15% to 136.47% compared to the other systems (Figure S10, Supporting Information).

Despite the clear emission reduction and economic advantages, the underlying mechanism for the low CH_4_ emissions from RR remains unclear. Given that CH_4_ production is fundamentally governed by methanogenic archaea and their functional genes, we hypothesized that the observed reduction is linked to differences in the abundance and function of methanogenic communities in the rhizosphere soil among MC, RR, and LR.

### 2.2 Changes in Functional Genes of Methane Production in Rhizosphere Soil Under Different Rice Cultivation Modes

**Figure 3:**
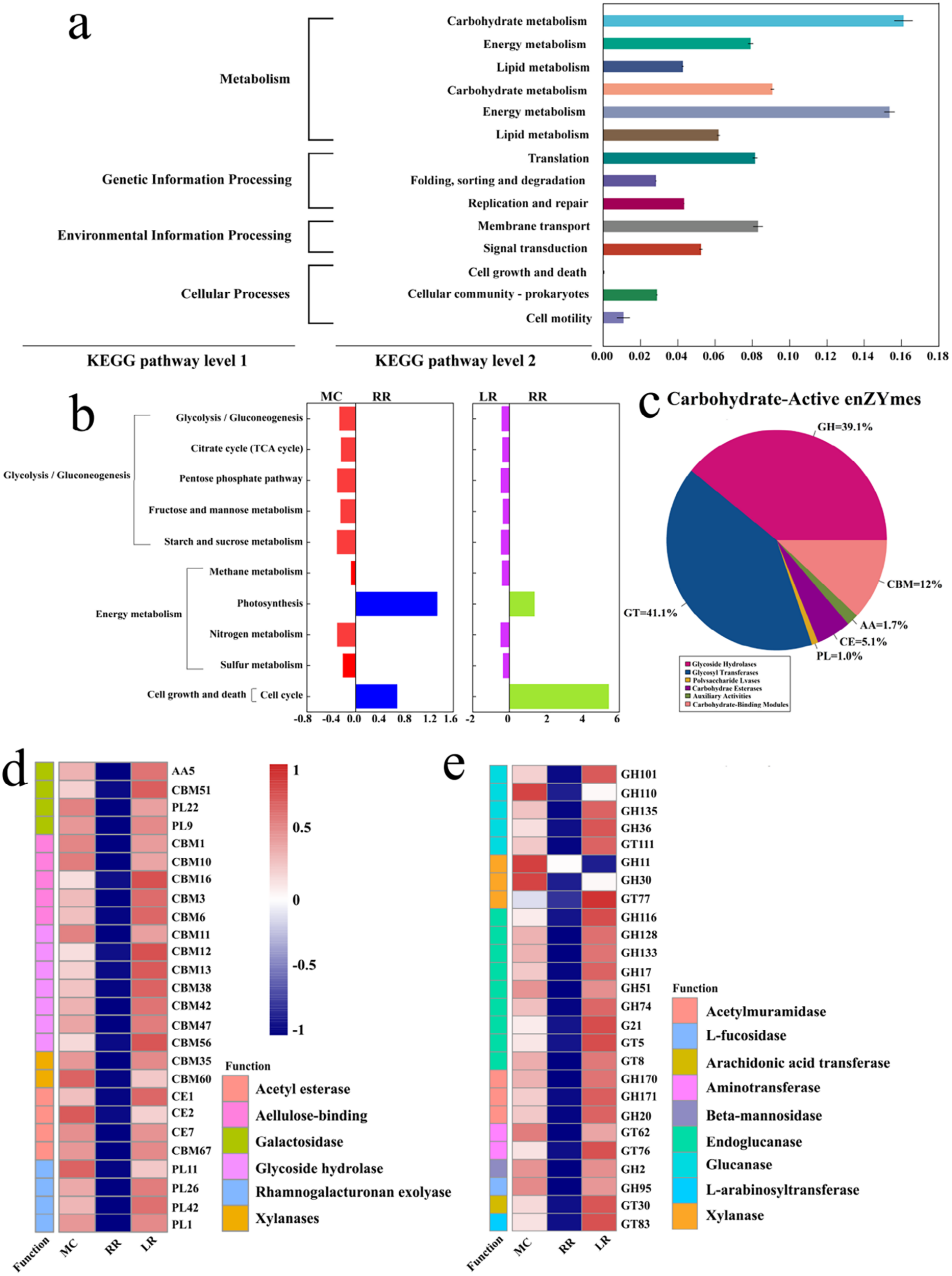
Metagenomics of different varieties of MC, RR and LR under different rice cultivation modes in 2022. a) Kyoto Encyclopedia of Genes and Genomes(KEGG) annotation pathways for metagenomics of YY1540 MC, RR and LR in 2022; Percentage of genes involved in different KEGG metabolic pathways in total genes in metagenomics. b) Comparison of total gene abundance differences in KEGG metabolic pathways among different treatments for YY1540 MC, RR and LR in 2022; an adjusted *p*‐value < 0.05 and |log_2_(fold change)| >2 was applied to define significantly up or down regulated genes. c) Proportion of different types of carbohydrate enzymes in the total carbohydrate enzymes for YY1540 MC, RR and LR in 2022; among which GH is a glycoside hydrolase, CBM is a carbohydrate binding enzyme, AA is an auxiliary modular enzyme, CE is a carbohydrate esterase, and PL is a polysaccharide lyase. d,e) Relative abundance of carbohydrate functional enzymes for YY1540 MC, RR and LR in 2022, the *p*‐value < 0.05 and |log_2_(fold change)| >2 adjusted using R software are used to define significantly upregulated or downregulated genes, with color levels indicating high (red) to low (blue) abundance. All figures were created using R software version 4.4.1. Source data are provided in the source data file.

The reduced CH_4_ emission was associated with downregulated expression of key methanogenic pathway genes in the resident microorganisms. To investigate the methane emission mechanism, a metagenomic analysis was performed. The metagenomic assembly yielded 1 507 575 genes annotated in the KEGG database, KEGG functional annotations were predominantly associated with carbohydrate metabolism, energy metabolism, amino acid metabolism, and cell growth or death (**Figure** **3**a). Among the differentially abundant KEGG pathways, key metabolic processes including sucrose and starch metabolism (within carbohydrate metabolism) and methane metabolism (within energy metabolism) were significantly downregulated in RR compared to both MC and LR (Figure 3b).

A total of 461 265 genes were assigned to the CAZy database. RR showed a significant reduction in the abundance of most genes associated with glycoside hydrolases (GHs), glycosyltransferases (GTs), and carbohydrate‐binding modules (CBMs) compared to MC and LR. This widespread downregulation indicates a suppressed capacity for microbial hydrolysis of organic matter into fatty acids and other organic acids in the RR rhizosphere (Figure 3c–e).

**Figure 4:**
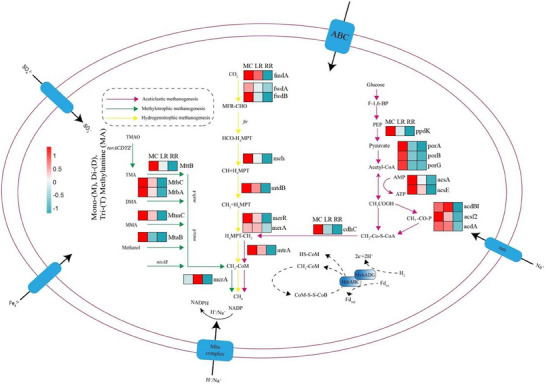
Metagenomic methane metabolism annotation pathways and gene differential heatmaps for YY1540 MC, RR and LR under different rice cultivation modes in 2022, an adjusted *p*‐value < 0.05 and |log_2_(fold change)| >2 was applied to define significantly up or down regulated genes, the color scale indicates high (red) to low (blue) abundance, graphics are created using R software version 4.4.1. Source data are provided in the source data file.

Metagenomic analysis identified 195 283 differentially abundant KEGG genes, including 1145 encoding enzymes essential for methanogenesis across the methylotrophic, CO_2_‐reductive, and acetoclastic pathways. The gene abundance for all three pathways was significantly lower in RR compared to MC and LR (**Figure** **4**). Within the CO_2_‐reductive pathway, key genes including methylene‐H_4_MPT cyclohydrolase (mch), methylene‐H_4_MPT reductase (mer), and the terminal methyl‐coenzyme‐M reductase (mcr) were downregulated by 0.4 to 2.8‐fold. Similarly, in the acetoclastic pathway, genes such as pyruvate‐phosphate dikinase (ppdK), acetyl‐CoA synthetase (acsA) and glutaryl‐CoA dehydrogenase (acdA) exhibited 0.3 to 3.2‐fold lower abundance.

Functionally, Mer and Mch facilitate the reduction of one‐carbon intermediates to organic acids that feed into the methanogenic electron transport chain, while Mcr catalyzes the final step of acetyl‐CoA reductive cleavage to CH_4_. Thus, their coordinated suppression directly impedes methane formation (Figure 4). Differences in functional genes of methanogen may be influenced by different rice root exudates. To directly identify the specific chemical drivers, we subsequently analyzed the composition of root exudates.

### 2.3 Root Exudate and Rhizosphere Soil Metabolomics under Different Rice Cultivation Modes

**Figure 5:**
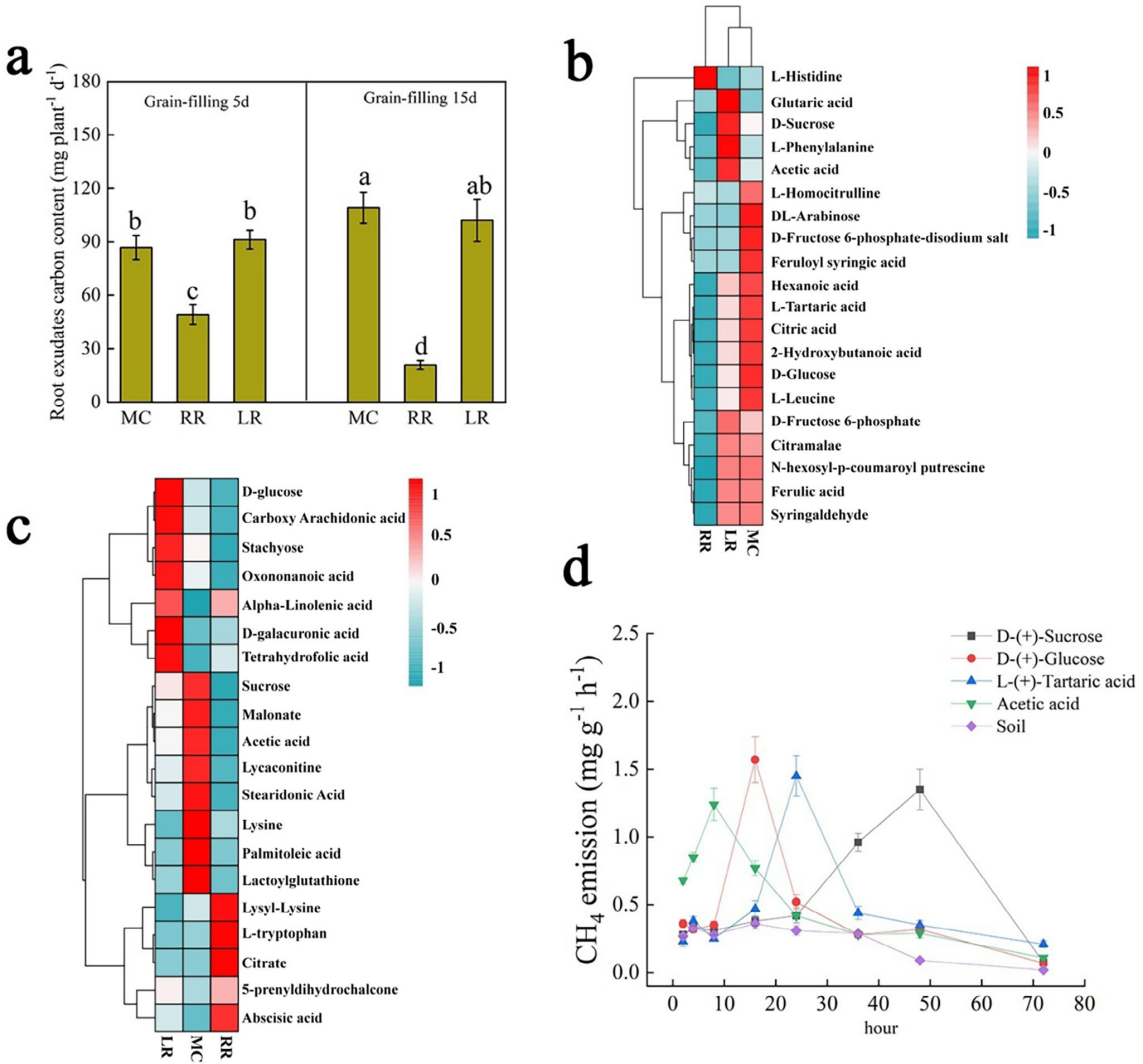
Metabolomics of different varieties of MC, RR and LR under different rice cultivation modes in 2022. a) Carbon content in root exudates on the 5th and 15th days for YY1540 MC, RR, and LR in 2022; Means ± SD. (n = 3 biological replicates), the different lowercase letters above the column graphs indicate significant differences by Duncan's test (*p* < 0.05). Figures were plotted using Origin 2021 software. b) Heatmap of differential metabolites in root exudates for YY1540 MC, RR, and LR in 2022; the *p*‐value < 0.05 and |log_2_(fold change)| >2 adjusted using R software version 4.4.1 are used to define significantly upregulated or downregulated metabolites, with color levels indicating high (red) to low (blue) abundance. c) Heatmap of differential metabolites in rhizosphere soil for YY1540 MC, RR, and LR in 2022; the *p*‐value < 0.05 and |log_2_(fold change)| >2 adjusted using R software version 4.4.1 are used to define significantly upregulated or downregulated metabolites, with color levels indicating high (red) to low (blue) abundance. d) CH_4_ emission flux after adding differential metabolites to the soil of YY1540 ratoon season rice. Means ± SD. (n = 3 biological replicates). Figures were plotted using Origin 2021 software. Source data are provided in the source data file.

We first assessed the composition of root exudates and found that the levels of acetic acid and carbohydrates were lower in RR than in MC and LR. Consistent with this, the total organic carbon content in RR exudates decreased by 43.3% to 80.8% (**Figure** **5**a). To comprehensively characterize these changes, we performed GC‐MS metabolomic analysis of root exudates at day 15 of the heading stage. We detected a total of 122 metabolites (File S2, Supporting Information). Principal component analysis (PCA) revealed a clear separation among the three cultivation modes (Figure S11, Supporting Information). The identified metabolites were primarily classified as organic acids, amino acids, sugars, and flavonoids based on KEGG pathway annotation (Figure S12, Supporting Information). Among them, key organic acids, including L‐(+)‐tartaric acid and acetic acid, as well as carbohydrates involved in central metabolism, such as D‐(+)‐sucrose, D‐(+)‐glucose, and D‐fructose, were significantly less abundant in RR compared to MC and LR (Figure S13, Supporting Information; Figure 5b).

To determine whether the observed differences in root exudates translated to the soil environment, we analyzed the rhizosphere soil metabolome. This analysis identified 612 metabolites (Figure S14, Supporting Information). Consistent with the root exudate profile, compounds in the carbohydrate metabolism pathway, notably acetic acid and sucrose, were significantly less abundant in the RR rhizosphere soil compared to MC and LR (Figures S15 and S16, Supporting Information; Figure 5c). To test the functional impact of these metabolites, we performed a spike‐in experiment by adding identified differential metabolites (sucrose, glucose, tartaric acid, acetic acid) to RR soil. All additions significantly stimulated CH_4_ emissions. Notably, acetic acid induced the most rapid response, reaching peak emission within 10 h, suggesting it is a preferred substrate for methanogenic microorganisms (Figure 5d). This spike effect was abolished in autoclaved RR soil, confirming that the response is driven by living microbiota (Figure S17, Supporting Information). Collectively, these results demonstrate that RR reduces the allocation of photosynthates‐including key substrates like acetic acid‐to the rhizosphere, thereby limiting the metabolic activity of methanogenic microorganisms and reducing CH_4_ emissions. Given that exudate composition reflects whole‐plant carbon partitioning, we subsequently tracked the in vivo allocation of photoassimilates.

### 2.4 Characteristics of Photosynthate Accumulation and Distribution in Different Rice Cultivation Modes

**Figure 6:**
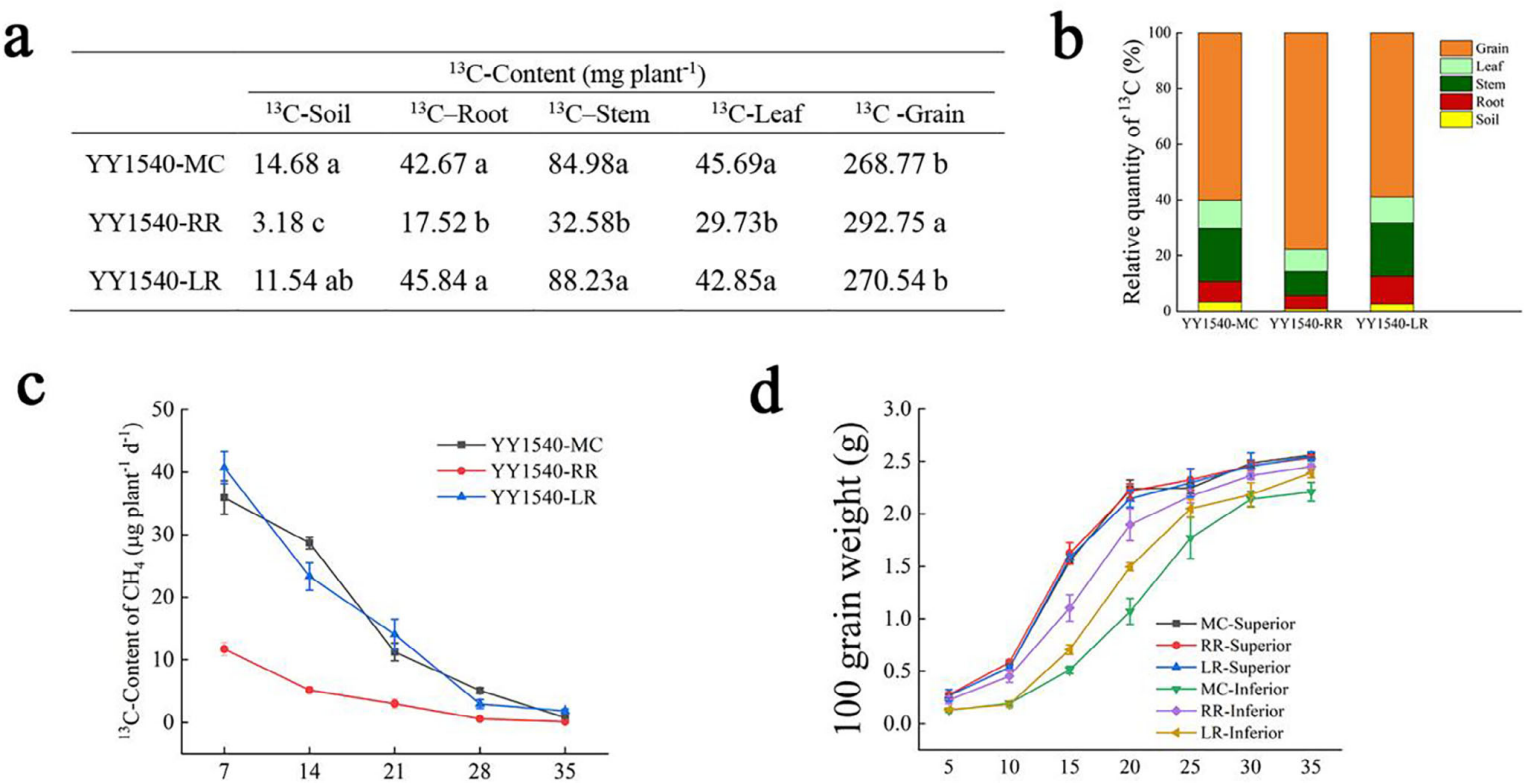
Photosynthate distribution for different varieties of MC, RR and LR under different rice cultivation modes in 2021 and 2022. a) ^13^C content in different tissues at maturity for YY1540 MC, RR and LR in 2022; Data are presented as n = 3 independent replicates, values with a column followed by different letters are significantly different by Duncan's test (*p* < 0.05). b) Relative distribution rate of ^13^C for YY1540 MC, RR and LR in 2022; Data are presented as n = 3 independent replicates. c) ^13^CH_4_ emissions for YY1540 MC, RR and LR in 2022; Means ± SD. (n = 3 biological replicates). d) Grain filling dynamics for superior grains and the inferior of YY1540 MC, RR and LR in 2022. Means ± SD. (n = 3 biological replicates). All the figure were plotted using Origin 2021 software. Source data are provided in the source data file.

To investigate photosynthate partitioning, we first conducted pot experiments, which reliably simulated field conditions as evidenced by comparable yield and CH_4_ emission patterns between the two systems (Figure S18, Supporting Information; Table S6, Supporting Information). These experiments confirmed that RR directs less photosynthate to its underground parts compared to MC and LR. Across genotypes, the non‐structural carbohydrate (NSC) export rate in RR was 41% to 231% higher than in MC and LR. (Tables S7 and S8, Supporting Information). ^13^CO_2_ pulse‐labelling revealed that RR allocated 29%–32% more newly fixed ^13^C to grains and 66%–78% less to the rhizosphere soil compared to MC and LR (**Figure** **6**a,b). Consequently, the evolution of ^13^CH_4_ derived from the labeled ^13^CO_2_ decreased by 67%–94% in RR (Figure 6c). This enhanced grain filling, particularly in inferior spikelets, was achieved at the direct expense of root carbon exudation (Figure 6d).

Source‐sink manipulation experiments further corroborated this carbon trade‐off. Panicle removal in LR increased CH_4_ emissions, indicating a redirection of carbon to the rhizosphere. Conversely, reciprocal soil transplantation between RR and LR plants altered emission patterns: RR soil transferred to LR plants reduced their CH_4_ emissions, while LR soil transferred to RR plants increased emissions. Moreover, soil sterilization at the heading stage, which did not alter soil physicochemical properties, significantly reduced CH_4_ emissions in both RR and LR (Figure S19, Supporting Information). These pot experiments collectively demonstrate that different cultivation modes regulate CH_4_ emissions through plant‐mediated modulation of the soil microbial community (Figure S20, Supporting Information). These findings support a model where RR reduces the allocation of photosynthetic products to the rhizosphere, leading to a decrease in root exudates (e.g., acetic acid). This, in turn, suppresses the function of methanogenic archaea and reduces CH_4_ emissions. To directly identify the microbes utilizing plant‐derived photosynthates and to eliminate interference from native soil organic carbon, we employed DNA‐based stable isotope probing (DNA‐SIP).

### 2.5 ^13^CDNA‐SIP under Different Rice Cultivation Modes

**Figure 7:**
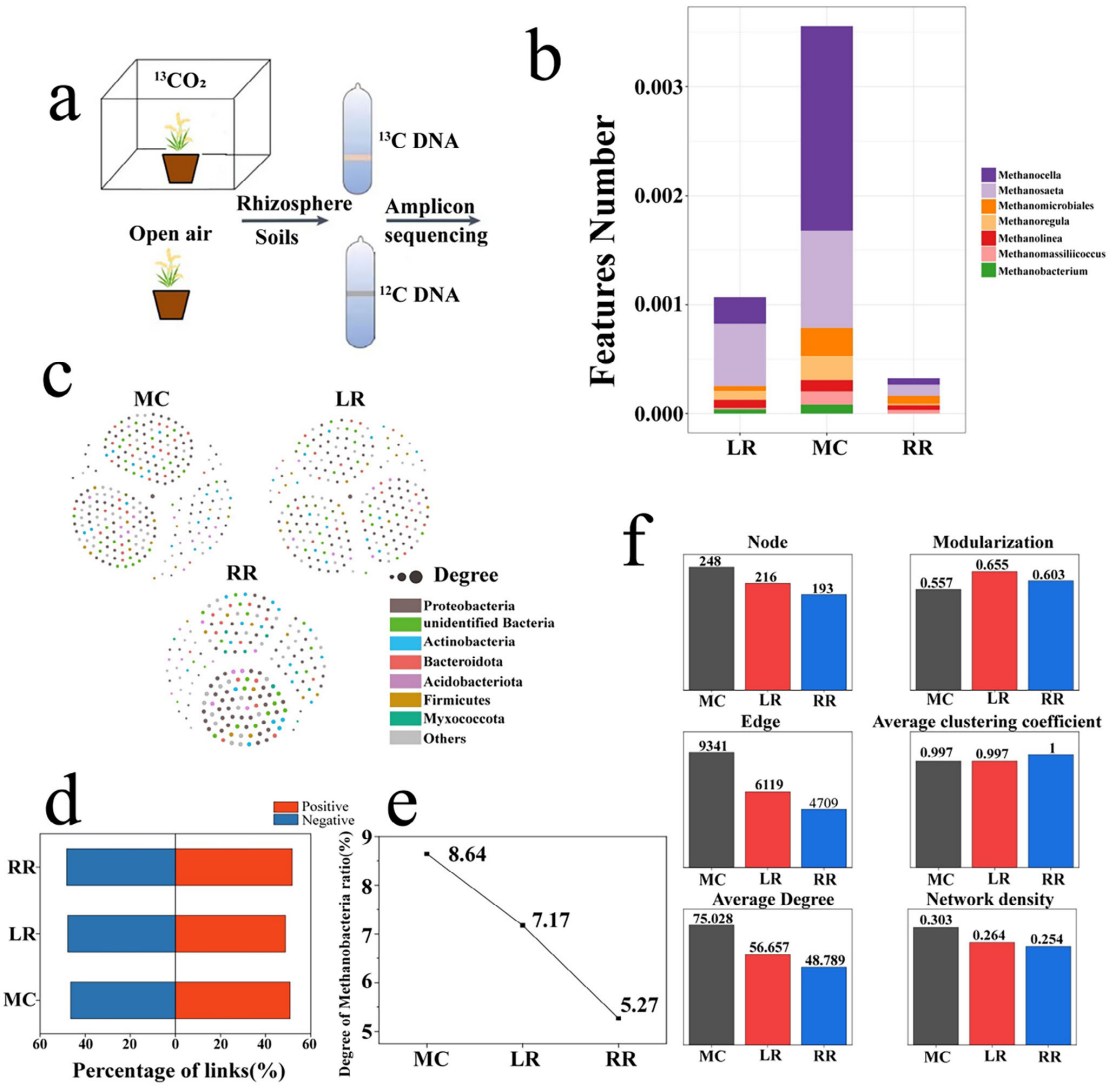
^13^C‐labeled DNA‐SIP of YY1540 MC, RR and LR under different rice cultivation modes in 2022. a) Schematic illustration of the ^13^C‐DNA‐SIP experimental workflow for YY1540. b) Relative abundance of major methanogenic genera in the ^13^C‐labeled DNA fraction. Data are presented as the mean of n = 3 independent replicates. c) Genus‐level co‐occurrence networks of the ^13^C‐labeled microbiota. Nodes represent microbial genera, and edges represent strong and significant (Spearman's |r| >0.65, *p* < 0.01) correlations. d) Proportion of positive (co‐occurrence) and negative (mutual exclusion) connections in the networks. e) Proportion of keystone nodes (hubs and connectors) identified as methanogenic archaea. f) Key topological properties of the microbial co‐occurrence networks. All data analysis and visualization for panels a–f were performed using R software version 4.4.1. Microbial co‐occurrence networks were constructed by generating correlation matrices with the R packages reshape2 and WGCNA, and visualized in Gephi 0.10.1. Only robust correlations (Spearman's |r| >0.65, *p* < 0.01) were retained as edges. Network topological properties were calculated using the R package igraph. Source data are provided as a Source Data file.

The utilization of ^13^C photosynthate by methanogens in the rhizosphere soil of ratoon season rice decreased. To trace which microbes specifically assimilate root‐exuded carbon, DNA was extracted from ^13^CO_2_‐labelled rhizosphere of MC, RR and LR and subjected to isopycnic CsCl centrifugation. We clearly observed a sharp contrast between the heavy density fraction and the light density control fraction in the DNA gradient, demonstrating successful isolation of the ^13^C‐labeled portion, representing organisms that had incorporated ^13^C from root exudates (**Figure** **7**a; Figure S21, Supporting Information). Amplicon sequencing of this heavy DNA yielded 17 078 bacterial/archaeal ASVs distributed across 42 phyla, 73 classes, 145 orders, 258 families and 432 genera (File S4, Supporting Information). PCoA revealed clear separation among the three cultivation modes (Figure S22, Supporting Information), confirming cultivation‐specific ^13^C‐microbiota. Dominant ^13^C‐utilising genera included Flavisolibacter, Pseudomonas, Sphingomonas, Massilia and Aquabacterium. RR significantly enriched Proteobacteria, Acidobacteriota, Gemmatimonadetes, Firmicutes, Myxococcota and Chloroflexi, but reduced methanotrophic/methanogenic taxa such as Methylomirabilota, Bacteroidota and additional Myxococcota (Figure S23, Supporting Information). Thus, RR enhances the relative abundance of ^13^C‐labelled heterotrophic bacteria while suppressing methanogenic archaea.

K‐means clustering partitioned ASVs into seven temporal/abundance clusters; clusters 1 and 6, assigned to methanogens, were lower in RR than in MC or LR (Figure S24 and S25, Supporting Information). Molecular ecological networks corroborated this shift: RR networks were smaller (193 nodes, 4709 links) than MC (249 nodes, 9341 links) or LR (216 nodes, 6119 links), and exhibited the lowest network density and average degree (Figure 7c,f), implying reduced microbial interaction strength. Most importantly, methanogenic genera constituted the smallest fraction of keystone nodes in RR (Figure 7d,e). Genus‐level quantification identified seven methanogens, all significantly less abundant in RR relative to MC. Methanobacterium, Methanolinea, Methanoregula, Methanocella and Methanosaeta were also lower than in LR, whereas Methanomicrobiales was undetectable in RR (Figure 7b).

Collectively, diminished exudation of acetate starves methanogens of their preferred substrates, translating reduced ^13^C allocation into lower archaeal abundance and, consequently, lower CH_4_ emission. However, the mechanism of the differences in the allocation of photosynthetic products in different rice cultivation modes is not clear. We therefore combined physiological assays with transcriptomic profiling to identify the key genes and pathways that skew photo‐assimilate flow toward grain at the expense of root exudation in ratoon season rice.

### 2.6 Interaction of ABA‐OsCIPK2‐OsSWEET1A Reduces Photosynthetic Product Allocation to the Rhizosphere

**Figure 8:**
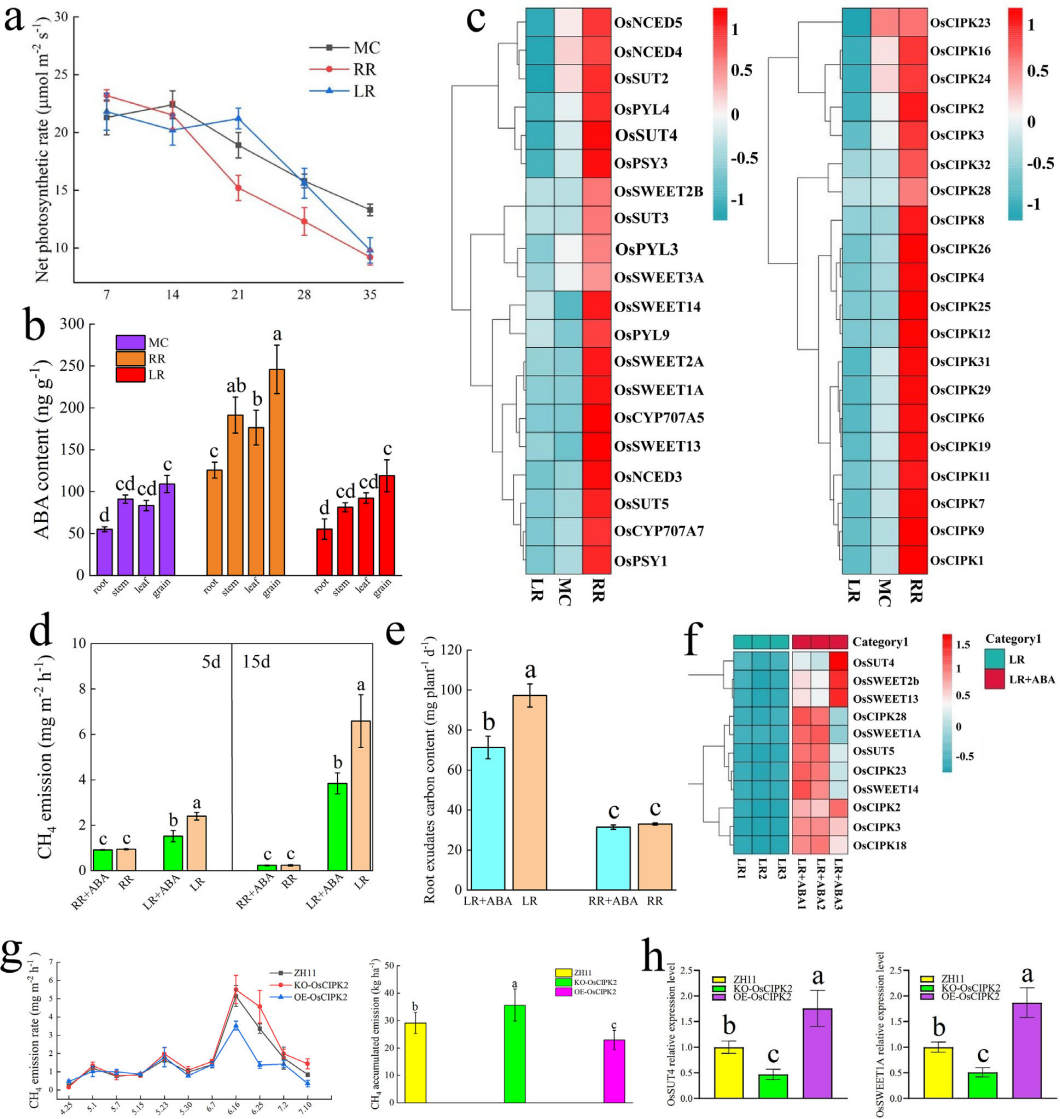
Transcriptome and differential gene function validation of YY1540 MC, RR and LR under different rice cultivation modes in 2022. a) Net photosynthetic rate of YY1540 MC, RR, and LR in 2022; Means ± SD. (n = 3 biological replicates) and figures were plotted using Origin 2021 software. b) ABA content of YY1540 MC, RR, and LR in 2022; Means ± SD. (n = 3 biological replicates), the different lowercase letters above the column graphs indicate significant differences by Duncan's test (*p* < 0.05). c) Heatmap of differentially expressed genes in the transcriptome of YY1540 MC, RR, and LR in 2022; Genes with a *p*‐value < 0.05 and |log_2_(fold change)| >2 are displayed. d) Effect of ABA treatment on CH_4_ emissions of YY1540 RR and LR on the 5th and 15th days; Means ± SD. (n = 3 biological replicates), the different lowercase letters above the column graphs indicate significant differences by Duncan's test (*p* < 0.05). e) Effect of ABA treatment on carbon content in root exudates of YY1540 RR and LR; Means ± SD. (n = 3 biological replicates), the different lowercase letters above the column graphs indicate significant differences by Duncan's test (*p* < 0.05). f) Heatmap of differential genes in the OsCIPK family, OSUT family, and OsSWEET family of YY1540 RR and LR under ABA treatment; Genes with a *p*‐value < 0.05 and |log_2_(fold change)| >2 are displayed. g) Effect of overexpression and knockout of OsCIPK2 on CH_4_ emission flux(left) and cumulative emissions(right); the CH_4_ emission flux through Means ± SD. (n = 3 biological replicates), the CH_4_ cumulative emissions through Means ± SD. (n = 3 biological replicates), the different lowercase letters above the column graphs indicate significant differences by Duncan's test (*p* < 0.05). h) Effect of overexpression and knockout of OsCIPK2 on the relative expression levels of OsSUT4 and OsSWEET1A. Means ± SD. (n = 3 biological replicates), the different lowercase letters above the column graphs indicate significant differences by Duncan's test (*p* < 0.05). All line and column graphs were generated using Origin 2021 software. Heatmaps were generated using R software version 4.4.1, with color levels indicating high (red) to low (blue) abundance. Source data are provided as a Source Data file.

Ratoon season rice reduces photosynthetic product allocation to the rhizosphere through the interaction of ABA‐OsCIPK2‐OsSWEET1A. Net photosynthesis in RR exceeded MC/LR at 7 and 14 d after heading but declined earlier but on the 21st and 28th days (**Figure** **8**a). On the 21st day post‐heading, the ABA content in roots, stems, leaves, and grains was significantly higher in RR compared to MC and LR (Figure 8b), suggesting that ABA accelerates assimilate remobilization during grain filling. RNA‐seq on day 21 separated the three treatments by PCA. RR versus MC/LR yielded 750/899 up‐regulated and 774/572 down‐regulated genes (Figure S26, Supporting Information). GO/KEGG enrichment highlighted ABA response, carbohydrate metabolism and transport. ABA‐biosynthesis genes (OsPSY1/3, OsNCED3/4/5) were induced, whereas the catabolic gene OsCYP707A6 was repressed. Receptors PYL3/4/9 and most OsCIPK family members (especially OsCIPK2) were up‐regulated, as were sucrose‐transport genes OsSUT2‐5 and OsSWEET1A/2A/2B/3A/13/14 (Figure 8c), indicating that ABA signaling primes the CIPK‐SWEET regulatory cascade.

When LR leaves were sprayed with ABA, root ABA rose to RR levels within 15 d (Figure S27, Supporting Information). After just 5 days of ABA application, a significant 36.40% reduction in daily CH_4_ flux was observed, with cumulative emissions decreasing by 41.6% by day 15 (Figure 8d). NSC in root, stem and leaf fell 15%–22%, root‐exudate carbon declined 21.4% (Table S9; Figure S28, Supporting Information; Figure 8e), and inferior‐grain filling improved, raising yield 4.6% (Figure S29; Table S10, Supporting Information). Expression of OsCIPK2/3/18/23/28 and OsSWEET1A/2B/13/14 was simultaneously elevated (Figure 8f). These results demonstrate that ABA directly recapitulates the RR phenotype by activating the expression of specific OsCIPK and sucrose transporter genes.

We selected the Japonica rice variety Zhonghua 11 (ZH11), with the most significant expression differences and the richest transcripts, to construct OsCIPK2 overexpression and knockout mutant materials. The results showed OsCIPK2 overexpression reduced CH_4_ emissions, with significant differences on June 16 and June 25. The OsCIPK2 mutant increased CH_4_ emissions, with significant differences on June 25. Compared to the wild‐type ZH11, OsCIPK2 overexpression significantly reduced cumulative CH_4_ emissions by 21.44%, while the OsCIPK2 mutant increased cumulative CH_4_ emissions by 22.29% (Figure 8g). Additionally, the study found that OsCIPK2 overexpression increased NSC transport rate by 5.87%–18.63%, while the OsCIPK2 mutant decreased NSC transport rate by 7.07%–11.57% (Table S11, Supporting Information). Compared to wild‐type ZH11, OsCIPK2 overexpression increased the ^13^C allocation to grains by 21.12% and reduced the soil allocation by 34.10%, while the OsCIPK2 mutant decreased the ^13^C allocation to grains by 17.64% and increased the soil allocation by 20.38% (Table S12; Figure S30, Supporting Information). OsCIPK2 overexpression increased yield by 11.33%, while the OsCIPK2 mutant reduced yield by 8.57% (Table S13, Supporting Information). OsCIPK2 overexpression increased the expression of OsSUT4 and OsSWEET1A in the sucrose transporter family (Figure 8h), indicating that OsCIPK2 functions, at least in part, through these transporters to reduce photosynthate allocation to the rhizosphere.

**Figure 9:**
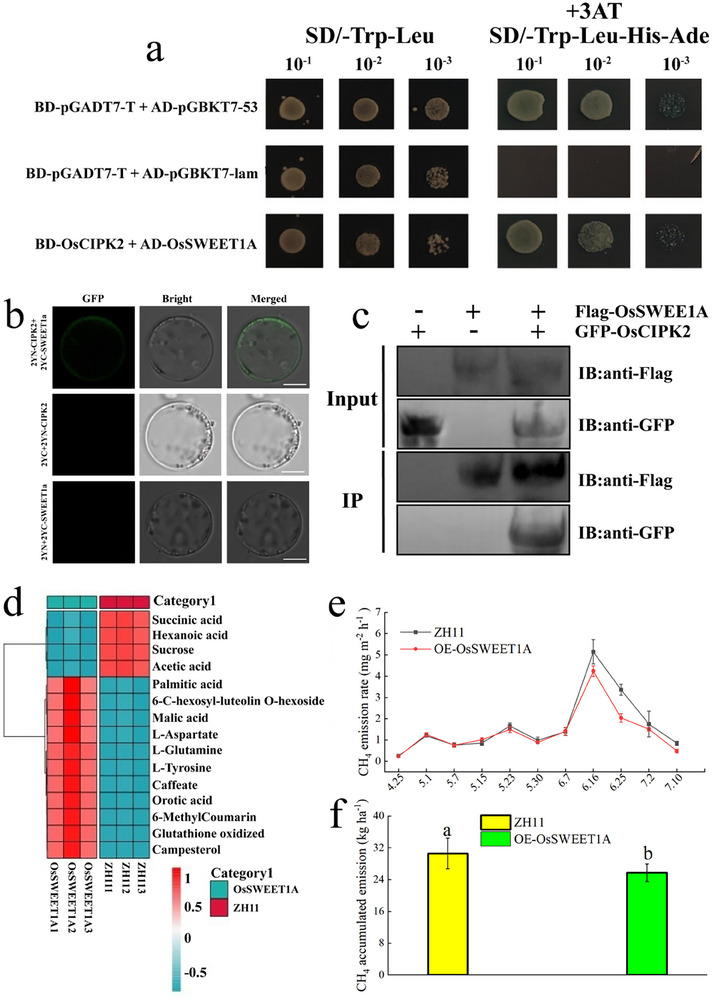
OsCIPK2 interacts with OsSWEET1A to regulate photosynthate distribution and reduce CH_4_ emissions. a) Yeast two‐hybrid (Y2H) assay showing the interaction between OsCIPK2 and OsSWEET1A. Yeast cells co‐expressing the indicated protein fusions were serially diluted (10^−1^ to 10^−3^) and spotted onto control medium (DDO: ‐Leu/‐Trp) and selective medium (QDO/X: ‐Ade/‐His/‐Leu/‐Trp with X‐α‐Gal). AD‐T + BD‐Lam and AD‐T + BD‐53 served as negative and positive controls, respectively. AD, GAL4 activation domain; BD, GAL4 DNA‐binding domain. b) BiFC detection of protein interaction between OsCIPK2 and OsSWEET1A; Confirmation of interaction between OsSWEET1A and OsCIPK2 using the bimolecular fluorescence complementation assay. Representative cells are shown, as imaged by laser‐scanning confocal microscopy. 2YN‐OsCIPK2/2YC and 2YC/2YN‐OsSWEET1A were used as the negative control. The empty vector was co‐expressed with each recombinant vector and used as a control. Scale bar = 20 µm. c) Reciprocal co‐immunoprecipitation (Co‐IP) of OsCIPK2 and OsSWEET1A in rice protoplasts. Protein complexes were immunoprecipitated using GFP‐Trap beads (for GFP‐OsCIPK2) or anti‐FLAG beads (for OsSWEET1A‐FLAG), followed by immunoblot analysis with the indicated antibodies. d) Heatmap of differentially abundant metabolites in root exudates from OsSWEET1A overexpression lines. Metabolites with a *p*‐value < 0.05 and |log_2_(fold change)| >2 are shown. The color scale represents relative abundance from high (red) to low (blue). e) CH_4_ emission flux from wild‐type (WT) and OsSWEET1A overexpression (OE) lines. Data are presented as Means ± SD (n = 3 biological replicates). f) Cumulative CH_4_ emissions from WT and OsSWEET1A‐OE lines. Data are presented as Means ± SD (n = 3 biological replicates). Different lowercase letters above the bars indicate significant differences determined by Duncan's test (*p* < 0.05). All line and column graphs were generated using Origin 2021 software. Heatmaps figure were generated using R software version 4.4.1. Source data are provided as a Source Data file.

To dissect how OsCIPK2 steers sucrose transport, we pulled down its interacting proteins from GFP‐OsCIPK2 transgenic leaves. Co‐IP/MS recovered 611 candidates enriched in photosynthesis, carbon and amino‐acid metabolism, glutathione cycling and organic‐acid turnover (Figure S31, Supporting Information). Among them, the sucrose transporters OsSUT4 and OsSWEET1A appeared. Yeast two‐hybrid validation showed that OsCIPK2 directly interacts with OsSWEET1A (**Figure** **9**a). Confocal laser‐scanning microscopy showed that when OsCIPK2‐YFP^N^ and OsSWEET1A‐YFP^C^ were co‐expressed transiently in rice protoplasts, strong green fluorescence was reconstituted at the plasma membrane, no fluorescence was observed in the negative controls (OsCIPK2‐YFP^N^ + YFP^C^ or YFP^N^ + OsSWEET1A‐YFP^C^). Thus, the BiFC assay demonstrates that OsCIPK2 and OsSWEET1A physically interact in vivo (Figure 9b). Lysates expressing GFP‐OsCIPK2 and OsSWEET1A‐FLAG were incubated with GFP‐Trap agarose beads (Chromotek) or FLAG antibody magnetic beads (Sigma), respectively. Western blotting of the immunoprecipitates revealed that FLAG antibody detected the OsSWEET1A‐FLAG fusion protein in the GFP‐Trap eluate, whereas GFP antibody detected the GFP‐OsCIPK2 fusion protein in the FLAG‐bead eluate. These Co‐IP data independently confirm the interaction between OsCIPK2 and OsSWEET1A (Figure 9c). This confirms that OsCIPK2 can regulate sucrose transport by interacting with OsSWEET1A and enhancing its expression.

To determine the functional outcome of this interaction, we generated OsSWEET1A overexpression lines. These lines exhibited a 14.00% reduction in cumulative CH_4_ emissions (Figure 9e,f), an increase in NSC transport rate of 5.92%–11.02% (Table S14, Supporting Information), and a 7.04% yield increase (Table S15, Supporting Information) compared to the wild‐type ZH11. Metabolomic analysis of the rhizosphere soil revealed that OsSWEET1A overexpression led to decreased levels of succinic acid, sucrose, and acetic acid (Figure 9d). In conclusion, our results delineate a mechanism in which ratoon rice elevates ABA levels, promoting the interaction and expression of OsCIPK2 and OsSWEET1A. This molecular module reduces the allocation of carbon substrates (e.g., acetic acid) to the rhizosphere, thereby inhibiting the activity of methanogenic archaea and ultimately reducing CH_4_ emissions.

## 3 Discussion

### 3.1 Ratoon Rice: A Synergistic Strategy for Methane Mitigation and Agricultural Sustainability

Our study demonstrates that ratoon season rice (RR) achieves dual benefits of greenhouse gas reduction and yield enhancement through optimized carbon allocation. Compared to MC and LR, RR directs more photosynthates to grains, concurrently reducing rhizospheric carbon inputs. This reallocation diminishes organic acids (e.g., acetic acid) and carbohydrates in root exudates, thereby suppressing methanogenic activity and associated gene expression (mcrA, Acs, Acd). The mechanism underlying this differential photosynthate distribution involves coordinated regulation by the ABA‐OsCIPK2‐OsSWEET1A module, which optimizes carbon allocation to the grain sink while correspondingly reducing its distribution to the rhizosphere.

This study found that, compared to MC and LR, RR increases daily yield by 34%–57% while reducing production costs by >42.86%, translating to 90%–136% higher economic returns per unit time. The results of the ^13^CO_2_ isotope distribution study confirmed that more ^13^C assimilates in RR were allocated to the grains (Figure 6), correspondingly reducing the allocation to the belowground part, thereby lowering the substrate content of plant‐derived ^13^C available for conversion into ^13^CH_4_ by rhizosphere soil methanogenic microbes (Figure 6c).^[^ ^14^ ^]^ Furthermore, reciprocal soil transplantation experiments revealed that the RR‐associated microbial community is a key driver of the observed phenotype. CH_4_ emissions decreased when LR plants were grown in RR soil, whereas emissions increased when RR plants were grown in LR soil. When the two soils were sterilized and exchanged for planting, there was no significant difference in CH_4_ emissions between treatments. This indicates that the reduction in CH_4_ emissions in RR is related to the reduced allocation of photosynthates to the rhizosphere,^[^ ^10^, ^11^, ^12^, ^13^, ^28^ ^]^ different rice cultivation patterns reduce CH_4_ emissions by altering the soil microbial environment. Methane emissions account for over 90% of greenhouse gases from rice paddies, representing 12% of agricultural greenhouse gas emissions, thus making them the primary source of greenhouse gas emissions in crop cultivation. The ecological implications are significant: China's current RR cultivation (1.1 million ha) already reduces methane emissions per yield by 41.3%. Scaling this model to the potential 4.5 million ha could boost national rice production by 16.9 million tons (8% of total output) while substantially mitigating agricultural methane—a critical step toward carbon neutrality. This positions RR as a scalable solution reconciling food security with climate goals.^[^ ^6^, ^29^, ^30^ ^]^

### 3.2 Methanogenesis Inhibition via Substrate Limitation in RR Rhizosphere

The microbial CH_4_ synthesis pathways can be divided into three types based on the substrates used: acetate pathway, H_2_/CO_2_ pathway, and methylated compound pathway. Studies have shown that the microbial methane synthesis pathway using acetate as a substrate is the main pathway for CH_4_ production in rice fields, accounting for about 90% of the CH_4_ produced. Metabolomic results show that RR reduces the carbohydrate and acetic acid content in root exudates. Adding differential metabolites to the rhizosphere soil of ratoon season rice can increase CH_4_ emissions, with acetic acid being directly utilized by methanogens to produce CH_4_ most rapidly. In contrast, sucrose and succinate need to be converted into acetate via the tricarboxylic acid cycle under the action of acetyl‐CoA to be utilized by methanogens. In the acetoclastic methanogenic pathway of the RR rhizosphere, the reduced availability of acetate leads to the downregulation of key enzymes such as acetyl‐CoA synthetase (Acs) and acetate‐CoA ligase (Acd). This, in turn, suppresses the entire pathway, including the final step catalyzed by methyl‐coenzyme M reductase (encoded by mcrA), thereby reducing CH_4_ production.

Meanwhile, using DNA‐SIP nucleic acid labeling technology, the microbial communities directly utilizing rice ^13^C photosynthates excreted into the soil were tracked, and the abundance of all methanogenic microbes at the genus level was analyzed. Results showed that there were seven methanogenic microbial communities, and their expression abundance in RR was significantly lower than in MC, particularly Methanobacterium, Methanolinea, Methanoregula, Methanocella, and Methanosaeta in RR, whose relative abundance was significantly lower than that of LR, and Methanomicrobiales were not detected in RR (Figure 7b). Metabolomic analysis of root exudates indicated that this result was mainly due to the reduction of methanogen‐available substrates (acetic acid) in root exudates of RR (Figure 5b), ultimately leading to reduced expression abundance of these methanogen functional groups. Currently, it is known that only methanogens from Methanosarcina and Methanosaeta can produce methane through the acetate fermentation pathway, with Methanosaeta being obligately acetoclastic.^[^ ^31^, ^32^ ^]^ Thus, it is believed that the reduction in the distribution of photosynthates to the belowground part in ratoon season rice reduces the content of carbon compounds like acetate and sucrose, leading to fewer substrates available for methanogens, inhibiting their abundance, and ultimately reducing the synthesis and emission of ^13^CH_4_ (Figure 6c).

### 3.3 ABA‐OsCIPK2‐OsSWEET1A Joint Regulation Reduces the Distribution of Photosynthetic Products to the Rhizosphere

This study also found that during the middle and late grain filling stages of RR, the ABA content in organs increases, net photosynthetic rate significantly decreases, ^13^C transfer rate increases, increasing the filling of inferior grains, improving seed setting rate and thousand‐grain weight, ultimately increasing the harvest index and daily yield of RR (Tables S3–S5, Supporting Information, Figure 8a,b).^[^ ^33^, ^34^ ^]^ Transcriptome analysis also found the expression of genes involved in ABA synthesis and receptor pathways was significantly upregulated, and the abundance of CIPKs and sugar transport proteins (SUT and SWEET) was also significantly upregulated. ABA, synthesized from β‐carotene precursors via enzymes like OsPSY and OsNCED, is perceived intracellularly by receptors such as PYL3/4/9.^[^ ^35^ ^]^ This ABA‐receptor complex then inhibits type 2C protein phosphatases (PP2Cs), thereby relieving the inhibition of SnRK2 kinases and enabling the phosphorylation and activation of downstream targets, including CIPKs.^[^ ^36^ ^]^ Activated CIPKs, potentially in a calcium‐dependent manner, subsequently phosphorylate sucrose transporters like SUTs and SWEETs to promote sucrose allocation.^[^ ^22^ ^]^ Previous studies have shown that RR regenerates from the axillary buds of the stubble of the MC after cutting. Due to the induced damage signal from cutting, the antioxidative capacity in the process of axillary bud ratoon is enhanced, and the content of ABA and IAA increases, accelerating the outward transport of stored dry matter from the stubble, stimulating axillary bud ratoon and grain filling, thereby improving yield and rice quality.^[^ ^37^, ^38^, ^39^ ^]^ This study also confirmed that exogenous ABA treatment of LR reduced the allocation of photosynthates to the rhizosphere, thereby reducing CH_4_ emissions (Figure 8). It was found that ABA promoting the expression of OsCIPK2, which interacts with OsSWEET1A to enhance the translocation of photosynthates to the grains and improve yield,^[^ ^40^ ^]^ while correspondingly reducing the allocation of photosynthates to the belowground part, leading to a reduction in the content of acetic acid and sucrose in root exudates, thereby lowering the substrate concentration for soil CH_4_ synthesis, ultimately affecting CH_4_ emissions. Thus, the reduction in CH_4_ emissions in RR is also related to a series of coordinated regulations by ABA‐OsCIPK2‐OsSWEET1A in the aboveground part. This result is also supported by previous related research conclusions.^[^ ^25^, ^26^, ^27^ ^]^

Ratoon rice system “one crop, two harvests” offers extra grain with lower methane, yet its spread is hindered by four practical bottlenecks: (i) stubbles harbor striped stem‐borer and brown planthopper, leading to heavier pest pressure; (ii) the crop needs moist soil 15–20 days before main‐crop harvest to awaken buds, but dry soil 7 d later for low‐stubble cutting‐conflicting water regimes; (iii) few varieties combine strong ratooning ability, high yield and local climate fitness, and breeding such lines is slow; (iv) management schedules (water, N, stubble height, harvest date) differ from those of single or double rice, demanding new skills and machinery that many farmers lack. Demonstration plots are still sparse, so visible success stories are rare. Breaking this impasse requires integrated action. Breeders should accelerate the release of pest‐tolerant, climate‐matched varieties with high ratoon vigor; researchers need to package simple, green protocols for pest, water and nutrient management that can run with existing machinery; extension services must expand on‐farm demonstrations and hands‐on training; and policymakers should offer start‐up subsidies, insurance and small‐scale irrigation upgrades to lower the adoption risk. With these combined measures, the environmental and economic gains shown here can move from experimental plots to farmer fields.

## 4 Conclusion 

**Figure 10:**
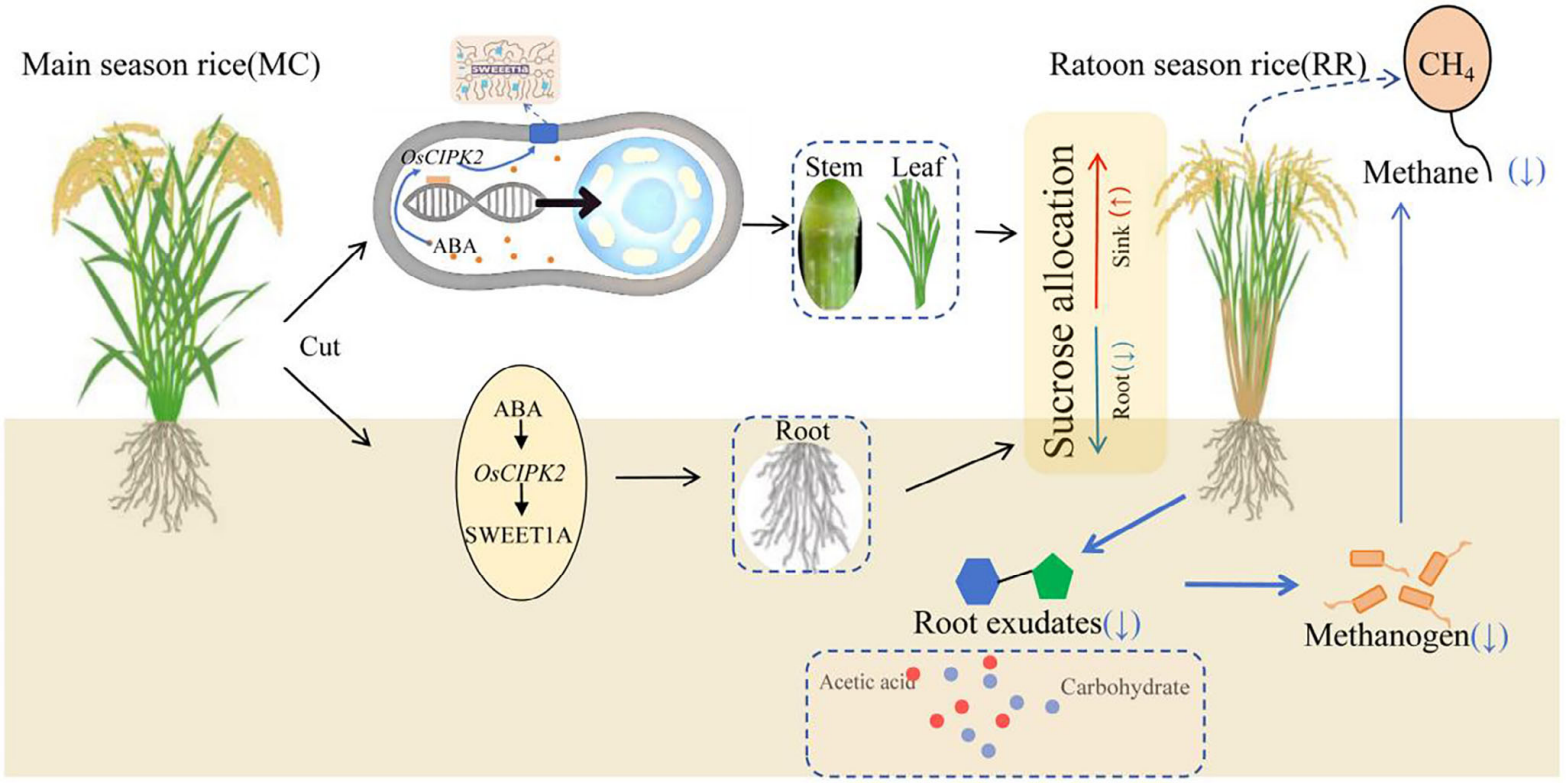
Schematic illustration of the mechanism underlying reduced CH_4_ emissions and increased yield in ratoon season rice (RR). Following the harvest of the main crop (MC), RR regenerates from the remaining stubble. The significant reduction in CH_4_ emissions from RR is driven by elevated abscisic acid (ABA) levels, which promote the interaction between OsCIPK2 and OsSWEET1A. This molecular cascade reduces the allocation of photosynthate‐derived carbon (e.g., acetate) to the rhizosphere, thereby limiting the carbon substrates available for methanogenic archaea and ultimately leading to lower CH_4_ emissions.

This study unveils a novel, bidirectional coordination mechanism in RR that links aboveground phytohormone signaling with belowground microbial processes to reduce CH_4_ emissions. The results clearly indicate that the reduction in CH_4_ emissions in ratoon season rice is through increased ABA content, leading to ABA‐OsCIPK2‐OsSWEET1A interactions decreased allocation of photosynthates to the rhizosphere, reducing the content of substrates (such as acetic acid) for CH_4_ synthesis in the soil, thereby inhibiting the methanogenesis process. These results provide new insights and pathways for further research on low‐carbon emission ratoon rice cultivation and molecular breeding (**Figure** **10**).

## 5 Experimental Section

### Experiment 1: Study on Methane Emissions and Methanogenic Function of Rhizospheric Soil—Experimental Sites and Period

This study was conducted over two consecutive rice‐growing years (March 2021 to December 2022) at the experimental base of Fujian Agriculture and Forestry University in Fuzhou, Fujian Province, China (26°5′N, 119°13′E; average altitude 12 m). The site experiences a subtropical monsoon climate. Figure S1, Supporting Information details the precipitation and average daily temperature during the 2021 and 2022 growing seasons. Additionally, a complementary experiment using the local variety ‘Yuxiang 1803′ (YX1803; main season growth period 110 days, ratoon season 75 days) was conducted during the ratoon season at the South Breeding Experimental Base of Hainan University in Yazhou District, Sanya City, Hainan Province, China (18°43′N, 108°36′E). The Hainan site has a tropical monsoon maritime climate with an average annual temperature of 25.4 °C, average annual rainfall of ≈1100 mm, and sunshine duration of 2287.3 h.

### Soil Characteristics

Fuzhou Site (2021): pH 5.3; total nitrogen (N) 1.42 g kg^−1^; available phosphorus (P) 22.83 mg kg^−1^; available potassium (K) 150.00 mg kg^−1^; organic matter 21.0 g kg^−1^.

Fuzhou Site (2022): pH 5.1; total N 2.16 g kg^−1^; available P 25.47 mg kg^−1^; available K 143.24 mg kg^−1^; organic matter 22.7 g kg^−1^.

Sanya Site (2023): pH 5.8; organic matter 15.62 g kg^−1^; total N 1.62 g kg^−1^; available P 81.33 mg kg^−1^; available K 72.65 mg kg^−1^.

Sanya Site (2024): pH 5.7; organic matter 21.42 g kg^−1^; total N 1.87 g kg^−1^; available P 122.45 mg kg^−1^; available K 55.47 mg kg^−1^.

### Rice Materials

Tested varieties included: Indica hybrid rice: ‘Taifengyou 3301′ (TFY3301), with a main season growth duration of 130 days. Japonica conventional rice: ‘Huanghuazhan’ (HHZ), 130 days. Indica‐japonica hybrid rice: ‘Yongyou 1540′ (YY1540) and ‘Yongyou 1526′ (YY1526), 145 days. Local indica variety (Sanya site only): ‘Yuxiang 1803′ (YX1803), 130 days. The ratoon season growth duration for all varieties was 75 days.

### Experimental Design and Cultivation Practices

The experiment employed a randomized complete block design with three replicates. Each plot area was 20 m^2^. Small dikes were constructed between plots to ensure independent irrigation and drainage. All varieties were sown on March 25th and transplanted on April 25th. Transplanting spacing was 30 cm × 15 cm, with three seedlings per hill.

### Definition and Management of Rice Cropping Patterns

Three distinct rice cropping patterns were implemented:

Main Crop Rice (MC): This represents the primary, single‐season rice cultivation cycle. HHZ, YX1803, and TFY3301 MC were harvested manually on July 25th, while YY1540 and YY1526 MC were harvested on August 10th. All MC plots were harvested to a low stubble height of 15 cm.

Fertilization (Fuzhou Site): Nitrogen (N) was applied as urea: 200 kg N ha^−1^ for TFY3301 and HHZ; 225 kg N ha^−1^ for YY1540 and YY1526. Application was split: 30% basal, 20% tillering, 30% panicle initiation, and 20% grain filling. Phosphorus (P) was applied as a single basal application (12% P_2_O_5_ fertilizer) at 562.5 kg ha^−1^. Potassium (K) was applied as 60% K_2_O fertilizer, split equally (50:50) between basal and grain filling stages, totaling 300 kg K ha^−1^. (The protocol for YX1803 in Sanya followed that of TFY3301 MC).

Ratoon Season Rice (RR): This system utilizes the regenerating tillers from the stubble left after the MC harvest to produce a second crop without replanting. It was initiated by the low stubble harvest (15 cm) of the preceding MC. The first fertilization, applied 3 days after MC harvest to promote ratoon tillering, consisted of urea at 90 kg N ha^−1^ (75% of the total N applied to RR). The second fertilization, applied during the RR heading stage to promote grain filling, consisted of urea at 30 kg N ha^−1^ (25% of the total RR N).

Late Rice (LR): This pattern involves raising seedlings and transplanting them into the field, with the heading stage synchronized to that of the RR. Except for being established ≈3 months later than the MC, the fertilization and cultivation practices for LR were identical to those for MC. This design enabled the comparison of greenhouse gas emissions between RR and LR under identical temperature and environmental conditions during the critical heading stage.

### Water Management

For MC and LR: A 3 cm water layer was maintained during the tillering stage. Irrigation was suspended 1 week prior to panicle initiation, while soil moisture was kept above 35% to suppress non‐productive tillers. A 3 cm water layer was re‐established during panicle initiation. Alternate wetting and drying (AWD) was implemented during the grain filling stage. Irrigation was terminated 10 days before harvest, reducing soil moisture to ≈30% at maturity.

For RR: A 3 cm water layer was maintained during the tillering stage, followed by AWD from grain filling to maturity.

Independent irrigation and drainage for each plot were ensured by the surrounding ridges.

### Pest and Disease Control

Strict control measures were implemented throughout the rice growth period using standard local practices to minimize yield loss and potential confounding effects on methane emissions.

### Weed Control

Manual weeding was employed exclusively to prevent any potential effects of herbicides on soil microorganisms and to effectively manage weed competition.

### Greenhouse Gas Emissions and Economic Benefits of Different Rice Cropping Patterns

During the 2‐year field experiment, greenhouse gases were collected from the MC, RR, and LR, every 7–10 days between 9 AM and 12 PM. The concentrations of CH_4_ and N_2_O in the collected gas samples were measured using a gas chromatograph (Agilent 7890B, USA). The samples were injected into 30 mL pre‐evacuated vials for analysis. CH_4_ gases were automatically detected by a hydrogen flame ionization detector (FID). The column temperature was set at 55 °C, and standard gases were provided by gas bottles stored in the laboratory. The emissions of CH_4_ and N_2_O were calculated according to the method mentioned by Qin et al.,^[^ ^41^ ^]^ and the flux of CH_4_ and N_2_O for each sample was calculated. The formula for calculating the flux of greenhouse gas emissions is as follows:

(1) $$F=60\times h\times \frac{\mathrm{Mw}\times 273.15}{22.41\times \left(273.15+T\right)}\times \frac{\mathrm{dC}}{\mathrm{dT}}$$

The formula for calculating the flux of greenhouse gas emissions is as follows: (F) is the greenhouse gas emission flux (mg m^−2^ h^−1^), 60 is the conversion factor to hours, (h) is the height of the gas collection chamber (m), (T) is the average temperature inside the chamber during sampling (°C),

(Mw) is the molar mass of the detected gas (g mol^−1^), (dC/dT) is the gas emission rate (1 × 10^−6^ L^−1^ min^−1^, i.e., ppmv min^−1^).

To evaluate the resource use efficiency of the MC, RR, and LR, this study conducted resource use accounting based on actual agronomic inputs and rice yield. According to Yu et al.,^[^ ^42^ ^]^ the net resources and resource use efficiency were calculated as follows:

(2) $$\mathrm{Net}\;\mathrm{Energy}=\mathrm{Energy}\;\mathrm{Output}\;\left(\mathrm{GJ}\;ha^{-1}\right)-\mathrm{Energy}\;\mathrm{Input}\;\left(\mathrm{GJ}\;ha^{-1}\right)$$

(3) $$\begin{array}{ccc} \mathrm{Resource} & \mathrm{Use} & \mathrm{Efficiency}\\ & & =\mathrm{Energy}\;\mathrm{Output}\;\left(\mathrm{GJ}\;ha^{-1}\right)\setminus \mathrm{Energy}\;\mathrm{Input}\;\left(\mathrm{GJ}\;ha^{-1}\right) \end{array}$$

In this study, the environmental footprint was determined using indirect carbon emissions and reactive nitrogen losses from agronomic inputs during production, processing, and transportation, as well as direct carbon emissions and field reactive nitrogen losses. The carbon footprint (CF) and nitrogen footprint (NF) were calculated using the methods described by Zou et al,^[^ ^7^ ^]^ with nitrogen content determined according to the method of Qin et al.^[^ ^41^ ^]^

The net ecosystem economic benefit is a comprehensive parameter for evaluating the economic benefits of cropping systems, calculated according to the method mentioned in previous studies^[^ ^42^ ^]^:

(4) $$\begin{array}{ccc} & \mathrm{Net} & \mathrm{Ecosystem}\mathrm{Economic}\;\mathrm{Benefit}=\mathrm{Economic}\;\mathrm{Output}\\ & & -\mathrm{Agricultural}\;\mathrm{Input}\;\mathrm{Costs}-\mathrm{Carbon}\;\mathrm{Footprint}\;\mathrm{Cost}\;\left(\mathrm{CF}\;\mathrm{cost}\right)\\ & & -\mathrm{Nitrogen}\;\mathrm{Footprint}\;\mathrm{Cost}\;\left(\mathrm{NF}\;\mathrm{cost}\right) \end{array}$$

At the maturity stage of each rice type, 20 rice plants were randomly selected from each treatment to calculate their yield and yield components, including the number of effective panicles, the number of grains per panicle, the seed setting rate, and the thousand‐grain weight.

### Metagenomic Sampling and Measurement of Rhizosphere Soil of YY1540 under Different Rice Cultivation Systems (Research on Functional Genes of Methanogenic Microorganisms)

Total soil DNA from each treatment was extracted using the Solarbio DNA extraction kit (Solarbio Technology, Beijing, China). Paired‐end reads (2 × 150 bp) were generated on the Illumina Hiseq4000 platform (Illumina Inc., San Diego, CA, USA). SOAPaligner was used to align high‐quality reads from each sample to the gene catalog (http://soap.genomics.org.cn/), with a standard of “identity >95%.” The obtained genes were annotated in databases to analyze their functions, including GO (Gene Ontology Consortium) for describing gene and protein functions, KEGG (Kyoto Encyclopedia of Genes and Genomes) for comprehensive information on biological genomes, pathways, and compounds, and CAZy (Carbohydrate‐Active Enzymes Database) for carbohydrate‐active enzymes based on published literature and related proteins.

### DNA Extraction, Purification, and Quantification of YY1540 under Different Rice Cultivation Systems

Total soil DNA was extracted using the Solarbio DNA extraction kit (Solarbio Technology, Beijing, China). Absolute quantification of the mcrA gene of methanogenic archaea was performed according to Zou et al.^[^ ^7^ ^]^

### Collection and Organic Carbon Content Measurement of Root Exudates of YY1540 under Different Rice Cultivation Systems

On the 5th and 15th days after heading of YY1540's RR and LR of the same period, three rice plants were randomly selected. The rice roots were carefully washed with sterile water until clean, then placed in a bucket containing 1 liter of ultrapure water with microbial inhibitors and cultured for 1 day. After removing the plants, the culture solution was collected, filtered, and nutrient ions were removed using resin to obtain root exudates.^[^ ^43^ ^]^ Each treatment was repeated three times for the measurement of organic carbon content in the rice plant exudates.^[^ ^44^ ^]^

### Metabolite Analysis of Root Exudates and Rhizosphere Soil of YY1540 under Different Rice Cultivation Systems (Study on Differences in Root Secretion Composition)

Root Exudates Metabolism: For each treatment, 1 g of root exudate sample collected from YY1540 MC, RR, and RR was placed in a 5 mL Eppendorf (EP) tube. Then, 1 mL of methanol: H_2_O = 3:1 (v/v) and 1 mL of chloroform, along with 10 µl of adonitol (0.5 mg mL^−1^) as an internal standard, were added. The samples were centrifuged at 12 000 rpm for 10 min at 4 °C with the addition of 1 mL of methanol: H_2_O = 3:1 (v/v) and 1 mL of chloroform. Gas chromatography‐mass spectrometry was performed using a Varian 240GC‐450MS with a FactorFour: VF‐5 ms column (30 m × 0.25 mm × 0.25 µm).^[^ ^7^ ^]^

Rhizosphere Soil Metabolism: For each rhizosphere soil sample collected from YY1540 MC, RR, and LR, 0.05 g was weighed and extracted with 1 mL of extraction solution containing an internal standard (methanol, acetonitrile, and water in a 2:2:1 ratio, internal standard concentration 20 mg L^−1^) and centrifuged at 12 000 rpm for 15 min at 4 °C. Metabolomics analysis was performed using a Waters Acquity I‐Class PLUS ultra‐high‐performance liquid chromatograph coupled with a Waters Xevo G2‐XS QTOF high‐resolution mass spectrometer. Mobile phase A was 0.1% formic acid aqueous solution; mobile phase B was 0.1% formic acid acetonitrile. The injection volume was 1 µL.

### Verification of Differential Metabolite Addition Test

Ten grams of rhizosphere soil from ratoon season rice YY1540 were collected into fifteen 100 mL double‐neck bottles. To each bottle, 10 mL of dd H_2_O was added. Every three bottles received 1 mL of 50 µmol L^−1^ solutions of sucrose, glucose, acetic acid, or tartaric acid, respectively, and CH_4_ emissions were measured at intervals of 0–80 h.

### Experiment 2: Analysis of the allocation mechanism of photosynthetic products in different rice cultivation modes—NSC Distribution in Different Rice Cultivation Modes

During the heading and maturity stages of rice, three representative plants of MC, RR, and LR from different rice cultivation modes were randomly selected.

The non‐structural carbohydrate (NSC) content (sucrose and starch) in different plant parts was determined according to the method described by Zou et al.^[^ ^7^ ^]^

Meanwhile, at the heading stage, panicles that bloomed on the same day and were relatively uniform in size were selected and marked with tags. From heading to 35 days post‐heading, five tagged panicles were taken every 5 days and divided into superior and inferior grains. The superior grains were the grains on the primary branches at the top three rachis, and the inferior grains were the grains on the secondary branches at the bottom three rachis of the panicle base.^[^ ^45^ ^]^ The two types of grain samples were placed in an oven, deactivated at 105 °C for 0.5 h, and then dried at 80 °C to constant weight for the determination of grain dry matter and its quality.^[^ ^37^ ^]^

### (^13^C) Isotope Labeling of YY1540 at Heading Stage in Different Rice Cultivation Modes

To trace assimilate partitioning in RR, MC and LR of cultivar YY1540 were grown in 20 kg field soil inside 33‐cm‐tall, 20‐cm‐diameter red pots (three seedlings per pot) and managed as in the field. The pots were placed in a growth chamber with conditions set at 22 °C, 16 h of light, and 8 h of darkness to observe their growth dynamics. At heading stage, plants were transferred to transparent acrylic cylinders (body + detachable lid, internal fan, sampling port) for pulsed ^13^CO_2_ labelling. After 2 h flushing with ambient air to deplete ^12^CO_2_, 1 L >99 at % ^13^CO_2_ was injected daily for 8 h over 10 consecutive days; cylinders were then refilled with ^12^CO_2_ to maximize ^13^C retention. Unlabeled pots 10 m away served as natural‐abundance controls. Soil, plant organs and head‐space gas were sampled every 7 days and analyzed by EA/HT‐IRMS (Thermo Fisher, Bremen) against PDB; δ^13^C and total C% were calculated following Zou et al.^[^ ^9^ ^]^

### Pan Exchange Cultivation and Panicle Trimming Regulation of YY1540 RR and LR in Different Planting Soils

Experiment 2: In 2022, a panicle trimming experiment was conducted using potted plants to explore the effect of grain sink regulation on the translocation and distribution of photosynthate. The pot experiment method was the same as described above, with three rice seedlings transplanted into each pot, for a total of 60 pots, and water and fertilizer management consistent with the field trials. On the 5th day after the full heading stage of YY1540 RR and LR, 20 uniformly growing rice plants were selected, and 50% of their panicles were trimmed. Greenhouse gas emissions were measured using the same method as the field trials.

During the heading stage of RR and LR, three pots each of LR and RR were carefully selected, and the plants were removed, and the soil on the roots was washed off before being transplanted into after sterilized soil at 130 °C. Additionally, three more pots each of LR and RR were selected, and after washing off the soil from the roots (LR plants were transplanted into RR soil and vice versa), greenhouse gas emissions were measured on the 5th and 20th days after transplantation to explore the impact of different rice soils on greenhouse gas emissions and their association with soil microorganisms.

### Sampling and Analysis of Rhizosphere Soil Samples of YY1540 under Different Rice Cultivation Systems

For YY1540 MC, RR, and LR, three rice plants were randomly selected. Soil adhering to the roots within 5 mm was considered rhizosphere soil. After collecting the rhizosphere soil, it was air‐dried naturally, passed through a 2 mm sieve to remove debris, and mixed into a composite sample. The sample was then preserved with dry ice, transported back to the laboratory, and stored at −80 °C for subsequent soil DNA extraction and related analyses.

### DNA‐SIP of YY1540 Rhizosphere (13C‐Labelled Photoassimilates Only)

We labeled potted plants with ^13^CO_2_ for 10 consecutive days during the heading stage of MC, RR, and LR, and collected rhizosphere soil at maturity, using unlabeled ^13^C rhizosphere soil as a control, the total DNA of 0.5 g ^13^C rhizosphere soil samples was extracted using the Solarbio DNA extraction kit (Solarbio Technology, Beijing, China). For density gradient centrifugation, 12.0 µg of DNA, 15 mL of 7.163 mol CsCl solution, and 3 mL of gradient buffer (GB) (0.1 mol Tris, 0.1 mol KCl, and 1 mmol EDTA, pH = 8.0) were mixed in a 50 mL sterile centrifuge tube. The initial buoyant density of the mixture was measured using a DR‐A1 digital Abbe refractometer (Atago Co., LTD, Tokyo, Japan). The mixture was then divided into three 5.2 mL centrifuge tubes (Beckman, product number 342 412). The tubes were sealed with a tube sealer (Beckman Coulter, USA) and centrifuged continuously for 48 h at 45 000 rpm at 4 °C in an Optima XPN‐80 ultracentrifuge (Beckman Coulter, USA).^[^ ^46^ ^]^ After centrifugation, DNA fractions were collected by replacing the gradient medium from the top of the ultracentrifuge tube with sterile water using an LSP01‐2A single syringe pump (Longer Pump, Baoding, Hebei, China) at a flow rate of 290 µL min^−1^, collected by a BSZ‐100 automatic fraction collector (Shanghai Jiapeng Technology Co., Ltd., China). A total of 15 density‐resolved DNA fractions were obtained. The ^13^C DNA was extracted to a concentration of 1 ng µL^−1^ and sequenced using NovaSeq 6000. Reads were trimmed (fastp), ASVs called (QIIME2 2023.2) and annotated against SILVA 138.1 (≥80% identity). Network topology (degree, betweenness, modularity, etc.) and modules (MCODE) were computed with Gephi and Cytoscape 3.9.1 for labelled archaea and bacteria.^[^ ^47^, ^48^ ^]^

### Measurement of Net Photosynthetic Rate of YY1540 under Different Rice Cultivation Systems (Exploring the Physiological Mechanisms of Photosynthetic Product Allocation between Ratoon and Single Season Rice)

On the day of full heading at 11:00, fully grown flag leaves were selected to measure the net photosynthetic rate (Pn) using a photosynthesis meter (Li‐6400; LiCor, Huntington Beach, CA, USA) with a CO_2_ concentration of 400 (CO_2_) mol^−1^, leaf temperature at 25 °C, and relative humidity ≈25%. Measurements were taken every 7 days.^[^ ^49^ ^]^

### Measurement of ABA Content in YY1540 under Different Rice Cultivation Systems

The abscisic acid (ABA) content of rice samples at different stages was measured using high‐performance liquid chromatography (HPLC).^[^ ^9^ ^]^

### Transcriptomics Measurement of YY1540 under Different Rice Cultivation Systems (Searching for the Molecular Mechanisms of Photosynthetic Product Allocation between Ratoon and Single season Rice)

For YY1540, MC, RR, and LR, and leaves were collected on the 15th day after heading for transcriptome analysis. Total RNA of rice was extracted using the RNA prep Pure Plant Kit (Tiangen, Beijing, China) according to the manufacturer's instructions. The total starting amount for library construction for each sample was 1 µg. The library was sequenced on the Illumina NovaSeq platform according to the manufacturer's instructions, generating 150 bp paired‐end reads. The Hisat2 tool software was used for alignment to the reference genome. Gene functions were annotated based on sequence alignment with the following databases: KO (KEGG Ortholog database); and GO (Gene Ontology).

### Experiment 3: (Verify the physiological mechanism hormone signaling gene regulatory network for the distribution of photosynthetic products between ratoon and single season rice)—Exogenous ABA Spraying Experiment on YY1540 during ratoon Season and Late Season at Full Heading Stage

On March 25, 2022, YY1540 rice seeds were sown and cultivated in a moist nursery bed. On April 25, three rice plants were transplanted into each plastic bucket. The height of the buckets was 33 cm a diameter of 20 cm. Each bucket contained 20 kg of soil collected from the 0–20 cm tillage layer of the same experimental field. Water and fertilizer management were the same as in the field experiment. On the first day of heading of YY1540 in both the RR and LR, 3 pots were selected and sprayed with 5 mL of 100 µmol L^−1^ ABA solution on the entire plant every night at 8:00 pm for 3 consecutive days. Samples were taken at the heading and maturity stages to measure NSC content, root exudate carbon content, yield components, and greenhouse gas emissions.

### Transgenic Material Verification Experiment

Total RNA extracted from wild‐type ZH11 leaves (Trizol) was reverse‐transcribed (PrimeScript RT, TaKaRa) to obtain full‐length cDNA. CDS of OsCIPK2 and OsSWEET1A were amplified with SnapGene‐designed primers carrying 16‐nt homologous arms, recombined into pCambia3301‐35S::GFP, and the resulting vectors introduced into Agrobacterium EHA105 by freeze‐thaw. Positive clones were verified by sequencing and used for Agrobacterium‐mediated transformation of ZH11;^[^ ^50^ ^]^ primer sequences are listed in Table S1, Supporting Information.

To generate OsCIPK2 CRISPR lines, two high‐spec‐score sgRNAs were selected with Cas‐Designer, synthesized as sgRNAF/R (Table S1, Supporting Information), annealed and cloned into BasI‐digested pAtU6‐M via T4 ligation. After sequence‐verification, the U6‐sgRNA cassette was PCR‐amplified, fused by infusion to p2 × 35S‐Cas9‐P2A‐GFP‐rbcS‐E9t, and the entire Cas9‐sgRNA expression module (KpnI/EcoRI fragment) was ligated into KpnI/EcoRI‐linearized binary vector pJim19(Genta). The resulting plasmid was electroporated into Agrobacterium tumefaciens AGL0, selected on LB containing spectinomycin and rifampicin (28 °C, 48 h), and used to infect ZH11 calli following standard protocols^[^ ^51^ ^]^ and the verification results are shown in Figures S2 and S3, Supporting Information.

Under growth chamber conditions of 22 °C with 16 h light/8 h dark, three seedlings per pot were grown, referencing field management and fertilization methods. Samples were taken at the full heading and maturity stages to measure NSC content, yield components, greenhouse gas emissions, and rhizosphere exudate metabolomics.

### Co‐Immunoprecipitation to Identification of OsCIPK2 Interacting Protein

Leaves of OsCIPK2‐GFP transgenic rice were ground in liquid nitrogen and extracted with ice‐cold Pi‐IP buffer plus protease inhibitors. After 12 000 × g, 4 °C, 15 min, the lysate was filtered through Miracloth. GFP‐Trap agarose (10–20 µL, Chromotek) was pre‐washed twice with Pi‐IP buffer, added to the extract, and rotated at 4 °C for 2–3 h. Beads were pelleted (2 000 × g, 1 min), proteins containing OsCIPK2 were confirmed by Western blot, gel‐excised, and subjected to mass spectrometry identification.^[^ ^51^ ^]^ Experimental samples were separated using the EASY‐nLC 1200 nanoflow HPLC system. Peptide samples were analyzed using the Q Exactive HF‐X mass spectrometer.

### Bimolecular Fluorescence Complementation (BiFC) Assay for Protein–Protein Interaction

The full‐length open reading frames (ORFs) of CIPK2 and SWEET1A were cloned into p2300‐2YN and p2300‐2YC, respectively, to generate transient‐expression constructs. Protoplasts isolated from the leaf sheaths of 10‐day‐old rice seedlings (cv. Zhonghua 11, ZH11) were co‐transfected with the two constructs or with the corresponding empty‐vector controls. After 12–16 h of dark incubation, fluorescence signals were acquired with a Leica SP8 laser‐scanning confocal microscope.

### Co‐Immunoprecipitation (Co‐IP) Assay for Protein–Protein Interaction

OsCIPK2 was cloned into a pCAMBIA1300 backbone with an N‐terminal GFP tag, and OsSWEET1A into the same backbone with a C‐terminal 3×FLAG tag. Both constructs were introduced into Agrobacterium tumefaciens EHA105. Protoplasts isolated from 7‐day‐old etiolated rice seedlings were transfected with GFP‐OsCIPK2 or OsSWEET1A‐FLAG alone, or co‐transfected with both. After 16 h, cells were lysed in 1 mL ice‐cold native buffer and cleared (12 000 rpm, 15 min, 4 °C); 100 µL supernatant was kept as input. The remainder was incubated with 25 µL GFP‐Trap agarose (ChromoTek) or 20 µL anti‐FLAG magnetic beads (Sigma) for 2 h at 4 °C with gentle rotation. Beads were washed three times, and bound proteins were eluted by boiling in 1× SDS loading buffer (8 min), resolved on 10% SDS‐PAGE, and immunoblotted with anti‐GFP and anti‐FLAG antibodies to verify the OsCIPK2–OsSWEET1A interaction.

### Yeast Two‐Hybrid Identification of OsCIPK2 and OsSWEET1A Interaction

The OsCIPK2 gene was cloned into the pGADT7 vector, and the OsSWEET1A gene was cloned into the pGBKT7 vector. Two plasmids (empty or fused plasmids of PGADT7 and PGBKT7, 1 µg each) and 5 µL of carrier DNA were added to Y2HGold competent cells. The yeast cells were resuspended in the premix by pipetting up and down. The mixture was centrifuged at 3,000 g for 3 min. 100 µL of double‐dropout liquid medium was added to resuspend the cells and plated on the corresponding dropout medium. Plates were incubated at 30 °C for 3–5 days, 3–5 positive clones were selected and cultured in 500 µL of double‐dropout liquid medium for 6 h, then centrifuged at 3000 g for 3 min. 10 µL of the yeast suspension was diluted tenfold and 100‐fold in ddH_2_O and spotted on double‐dropout and triple‐dropout solid medium. Plates were incubated upside down at 30 °C for 12 h to observe yeast growth and photographed.^[^ ^50^ ^]^

### Real‐Time Reverse Transcription Polymerase Chain Reaction

Actin was used as an internal reference, and primers spanning introns were designed using the NCBI database (https://www.ncbi.nlm.nih.gov/genbank/). PCR amplification was used to check the quality of the reverse transcription. Gene primers were designed using Primer Express 3.0.1 software, and primers are listed in Table S2, Supporting Information. Gene expression was calculated using the 2^−ΔΔCt^ method and repeated three times.^[^ ^50^ ^]^

### Statistical Analysis

All measurements included no fewer than three biological replicates. Statistical significance was assessed using one‐way analysis of variance (ANOVA) followed by Duncan's multiple range test in IBM SPSS Statistics 20.0, with the significance threshold set at *p* < 0.05. Results are presented as mean ± standard deviation (SD) and figures were plotted using Origin 2021 software. Heatmaps were generated from data standardized by *p*‐value < 0.05 and |log_2_(fold change)| >2. Microbial community networks were constructed by generating correlation matrices with the R packages reshape2 and WGCNA, and subsequently visualized in Gephi 0.10.1. Only edges with Spearman's correlation coefficients |r| >0.65 and *p* < 0.01 were retained. Network topological properties were calculated using the R package igraph.

## Conflict of Interest

The authors declare no conflict of interest.

## Author Contributions

J.Z.: Conceptualization, Methodology, Investigation, Writing – original draft, Writing – review & editing. H.X., C.L., B.Q., J.L., H.Z., C.G., H.C., Z.F., and Q.Z.: Investigation, Formal analysis, Visualization. C.F., Z.Z., and W.L.: Investigation, Data curation.

## Availability of Data and Materials

All physiological measurements that support the results of this study are included in this paper and its data file: Primary document dataset.xlsx; dataset.xlsx; File 1.xlsx; File 2.xlsx; File 3.xlsx;

The transcriptomics data supporting the findings of this study are available in Zenodo and can be accessed via the following URL: https://doi.org/10.5281/zenodo.13831141.

The metagenomic data supporting the findings of this study are available in Zenodo and can be accessed via the following URL: https://doi.org/10.5281/zenodo.13729947.

The microbiome data has been deposited in the NCBI database with accession number PRJNA1158317 and can be accessed via the following URL: https://www.ncbi.nlm.nih.gov/sra/PRJNA1158317.

## Supporting information



Supporting Information

Supporting Information

## References

[1] IPCC , Contribution of Working Group I to the Sixth Assessment Report of the Intergovernmental Panel on Climate Change, Cambridge University Press, Cambridge 2021.

[2] H. Zhou , F. Tao , Y. Chen , L. Yin , Y. Li , Y. Wang , C. Su , Sci. Total Environ. 2024, 935, 173441. 38782289 10.1016/j.scitotenv.2024.173441

[3] J. Liu , X. Huang , H. Jiang , H. Chen , Agric. Water Manage. 2021, 245, 106667.

[4] Y. Kwon , J.‐Y. Lee , J. Choi , S.‐M. Lee , D. Kim , J.‐K. Cha , H. Park , J.‐W. Kang , T. H. Kim , H. G. Chae , N. R. Kabange , K.‐W. Oh , P. J. Kim , Y.‐S. Kwak , J.‐H. Lee , C.‐M. Ryu , Nat. Clim. Change 2023, 13, 1329.

[5] W. X. Lin , J. Integr. Agric. 2019, 18, 246.

[6] S. Peng , C. Zheng , X. Yu , Crop Environ. 2023, 2, 5.

[7] J. Zou , H. Xu , C. Lan , B. Qin , J. Li , W. J. Nyimbo , H. Lin , Z. Pang , N. Fallah , C. Guo , C. Fang , Z. Zhang , H. Alwathnani , C. Rensing , H. Chen , W. Lin , Field Crops Res. 2024, 312, 109385.

[8] W. Wang , A. He , G. Jiang , H. Sun , M. Jiang , J. Man , X. Ling , K. Cui , J. Huang , S. Peng , L. Nie , Adv. Agron. 2020, 159, 135.

[9] J. Zou , Z. Pang , Z. Li , C. Guo , H. Lin , Z. Li , H. Chen , J. Huang , T. Chen , H. Xu , B. Qin , P. Letuma , W. Lin , W. Lin , J. Integr. Agric. 2024, 23, 806.

[10] S. Yuan , K. G. Cassman , J. Huang , S. Peng , P. Grassini , Field Crops Res. 2019, 234, 66. 31007365 10.1016/j.fcr.2019.02.004 PMC6472545

[11] X. Yu , S. Yuan , X. Tao , J. Huang , G. Yang , Z. Deng , L. Xu , C. Zheng , S. Peng , Sci. Total Environ. 2021, 24, 149246. 10.1016/j.scitotenv.2021.149246 34358744

[12] Y. Xu , L. Liang , B. Wang , J. Xiang , M. Gao , Z. Fu , P. Long , H. Luo , C. Huang , Sci. Total Environ. 2022, 813, 152550. 34952059 10.1016/j.scitotenv.2021.152550

[13] K. Song , G. Zhang , H. Yu , H. Xu , S. Lv , J. Ma , Eur. J. Soil Sci. 2021, 72, 1478.

[14] Y. Liu , T. Ge , Z. Zhu , S. Liu , Y. Luo , Y. Li , P. Wang , O. Gavrichkova , X. Xu , J. Wang , J. Wu , G. Guggenberger , Y. Kuzyakov , Soil Biol. Biochem. 2019, 133, 97.

[15] Y. Jiang , K. J. van Groenigen , S. Huang , B. A. Hungate , C. van Kessel , S. Hu , J. Zhang , L. Wu , X. Yan , L. Wang , J. Chen , X. Hang , Y. Zhang , W. R. Horwath , R. Ye , B. A. Linquist , Z. Song , C. Zheng , A. Deng , W. Zhang , Global Change Biol. 2017, 23, 4728. 10.1111/gcb.13737 28464384

[16] J. Su , C. Hu , X. Yan , Y. Jin , Z. Chen , Q. Guan , Y. Wang , D. Zhong , C. Jansson , F. Wang , A. Schnürer , C. Sun , Nature 2015, 523, 602. 26200336 10.1038/nature14673

[17] P. Li , J. Liu , M. Saleem , G. Li , L. Luan , M. Wu , Z. Li , Microbiome. 2022, 10, 108. 35841078 10.1186/s40168-022-01287-y PMC9287909

[18] Y. Li , R. Yang , M. M. Häggblom , M. Li , L. Guo , B. Li , M. Kolton , Z. Cao , M. Soleimani , Z. Chen , Z. Xu , W. Gao , B. Yan , W. Sun , Microbiome. 2022, 10, 186. 36329505 10.1186/s40168-022-01379-9 PMC9632085

[19] L. Qian , X. Yu , H. Gu , F. Liu , Y. Fan , C. Wang , Q. He , Y. Tian , Y. Peng , L. Shu , S. Wang , Z. Huang , Q. Yan , J. He , G. Liu , Q. Tu , Z. He , Microbiome. 2023, 11, 71. 37020239 10.1186/s40168-023-01501-5 PMC10074775

[20] J. Yang , J. Zhang , Z. Wang , K. Liu , P. Wang , J. Exp. Bot. 2006, 57, 149. 16330527 10.1093/jxb/erj018

[21] G.‐Q. Wang , H.‐X. Li , L. Feng , M.‐X. Chen , S. Meng , N.‐H. Ye , J. Zhang , J. Exp. Bot. 2019, 70, 1597. 30690492 10.1093/jxb/erz010 PMC6411378

[22] Q.‐J. Ma , M.‐H. Sun , H. Kang , J. Lu , C.‐X. You , Y.‐J. Hao , Plant Cell Environ. 2019, 42,918. 29791976 10.1111/pce.13349

[23] Q.‐J. Ma , M.‐H. Sun , J. Lu , Y.‐J. Liu , D.‐G. Hu , Y.‐J. Hao , Plant Physiol. 2017, 174, 2348. 28600345 10.1104/pp.17.00502 PMC5543958

[24] Q.‐J. Ma , M.‐H. Sun , Y.‐J. Liu , J. Lu , D.‐G. Hu , Y.‐J. Hao , Plant Physiol. Biochem. 2016, 109, 442. 27816825 10.1016/j.plaphy.2016.10.026

[25] L.‐B. Wu , J.‐S. Eom , R. Isoda , C. Li , S. N. Char , D. Luo , V. Schepler‐Luu , M. Nakamura , B. Yang , W. B. Frommer , New Phytol. 2022, 234, 975. 35211968 10.1111/nph.18054

[26] S. Wang , S. Liu , J. Wang , K. Yokosho , B. Zhou , Y.‐C. Yu , Z. Liu , W. B. Frommer , J. F. Ma , L.‐Q. Chen , Y. Guan , H. Shou , Z. Tian , Natl. Sci. Rev. 2020, 7, 1776. 34691511 10.1093/nsr/nwaa110 PMC8290959

[27] Y. Wu , S.‐K. Lee , Y. Yoo , J. Wei , S.‐Y. Kwon , S.‐W. Lee , J.‐S. Jeon , G. An , Mol. Plant 2018, 11, 833. 29656028 10.1016/j.molp.2018.04.002

[28] J. Hu , M. Bettembourg , L. Xue , R. Hu , A. Schnürer , C. Sun , Y. Jin , J. F. Sundström , Sci. Total Environ. 2024, 920, 170980. 38373456 10.1016/j.scitotenv.2024.170980

[29] T. Chen , P. Weng , C. Lan , F. Nyumah , C. Guo , W. Lin , Z. Zhang , H. Chen , W. Lin , Technol. Agron. 2024, 4, 0015.

[30] W. Lin , P. Weng , W. Lin , C. Shao , C. Guo , Z. Li , H. Chen , T. Chen , Chin. J. Appl. Ecol. 2024, 35, 827. 10.13287/j.1001-9332.202403.008 38646771

[31] O. V. Danilova , N. E. Suzina , J. Van De Kamp , M. M. Svenning , L. Bodrossy , S. N. Dedysh , Microb. Ecol. J. 2016, 10, 2734. 10.1038/ismej.2016.48 PMC5113839 27058508

[32] E. Damm , B. Rudels , U. Schauer , S. Mau , G. Dieckmann , Sci. Rep. 2015, 5, 1334. 10.1038/srep16179 PMC4639778 26553610

[33] Z. Wang , Y. Xu , T. Chen , H. Zhang , J. Yang , J. Zhang , Planta 2015, 241, 1091. 25589060 10.1007/s00425-015-2245-0

[34] K.‐Y. Zhu , J.‐Q. Yan , Y. Shen , W.‐Y. Zhang , Y.‐J. Xu , Z.‐Q. Wang , J.‐C. Yang , J. Integr. Agric. 2021, 21, 947.

[35] H. M. Nonhebel , K. Griffin , Funct. Plant Biol. 2020, 47, 716. 32438973 10.1071/FP19291

[36] K. Chen , G.‐J. Li , R. A. Bressan , C.‐P. Song , J.‐K. Zhu , Y. Zhao , J. Integr. Plant Biol. 2020, 62, 25. 31850654 10.1111/jipb.12899

[37] J. Huang , Y. Pan , H. Chen , Z. Zhang , C. Fang , C. Shao , H. Amjad , W. Lin , W. Lin , Field Crops Res. 2020, 258, 107962.

[38] F. Lin , J. Huang , S. Lin , P. Letuma , D. Xie , C. Rensing , W. Lin , J. Sci. Food Agric. 2023, 103, 3569. 36257928 10.1002/jsfa.12278

[39] F. Lin , C. Rensing , Z. Pang , J. Zou , S. Lin , P. Letuma , Z. Zhang , W. Lin , Field Crops Res. 2022, 283, 108521.

[40] V. Radchuk , Z. M. Belew , A. Gündel , S. Mayer , A. Hilo , G. Hensel , R. Sharma , K. Neumann , S. Ortleb , S. Wagner , A. Muszynska , C. Crocoll , D. Xu , I. Hoffie , J. Kumlehn , J. Fuchs , F. F. Peleke , J. J. Szymanski , H. Rolletschek , H. H. Nour‐Eldin , L. Borisjuk , Plant Cell 2023, 35, 2186. 36857316 10.1093/plcell/koad055 PMC10226576

[41] B. Qin , J. Zou , L. Cao , M. Wang , Y.‐X. Zhang , Agric. Ecosyst. Environ. 2022, 342, 108235.

[42] X. Yu , L. Xu , S. Yuan , G. Yang , H. Xiang , Y. Fu , J. Huang , S. Peng , J. Cleaner Prod. 2023, 393, 136249.

[43] P. Pétriacq , A. Williams , A. Cotton , A. E. McFarlane , S. A. Rolfe , J. Ton , Plant J. 2017, 92, 147. 28742258 10.1111/tpj.13639 PMC5639361

[44] C. Wang , L. Xue , Y. Dong , R. Jiao , Sci. Total Environ. 2021, 758, 143695. 33257064 10.1016/j.scitotenv.2020.143695

[45] Z. Zhang , H. Zhao , F. Huang , J. Long , G. Song , W. Lin , Plant J. 2019, 99, 344. 30912217 10.1111/tpj.14329

[46] J. Wang , X. Zhang , H. Yao , J. Microbiol. Methods 2020, 173, 105938. 32360380 10.1016/j.mimet.2020.105938

[47] Q. Min , Z. Wu , J. Yao , S. Wang , L. Duan , S. Liu , M. Zhang , Y. Luo , D. Ye , Y. Huang , L. Chen , K. Xu , J. Zhou , Cardiovasc. Diabetol. 2024, 23, 54. 38331798 10.1186/s12933-024-02144-y PMC10854096

[48] M. M. Yuan , X. Guo , L. Wu , Y. Zhang , N. Xiao , D. Ning , Z. Shi , X. Zhou , L. Wu , Y. Yang , J. M. Tiedje , J. Zhou , Nat. Clim. Change 2021, 11, 343.

[49] J. Zou , H. Yu , Q. Yu , X. Jin , L. Cao , M. Wang , M. Wang , C. Ren , Y. Zhang , Ind. Crops Prod. 2021, 163, 113323.

[50] L. Chen , H. Yang , Y. Fang , W. Guo , H. Chen , X. Zhang , W. Dai , S. Chen , Q. Hao , S. Yuan , C. Zhang , Y. Huang , Z. Shan , Z. Yang , D. Qiu , X. Liu , L.‐S. P. Tran , X. Zhou , D. Cao , Plant Biotechnol. J. 2021, 19, 702. 33098207 10.1111/pbi.13496 PMC8051608

[51] Y. Zhao , Y. Shi , G. Jiang , Y. Wu , M. Ma , X. Zhang , X. Liang , J.‐M. Zhou , New Phytol. 2022, 234, 607. 35090194 10.1111/nph.17997

