# Supplementary material for: Ratoon Season Rice Reduces Methane Emissions by Limiting Acetic Acid Transport to the Rhizosphere and Inhibiting Methanogens
Source: Adv Sci (Weinh). 2025 Nov 26;13(8):e07916. doi: 10.1002/advs.202507916 (PMC12884797; doi:10.1002/advs.202507916)
Supplement: Supplementary file 1 — Supporting Information [file ADVS-13-e07916-s002.docx]

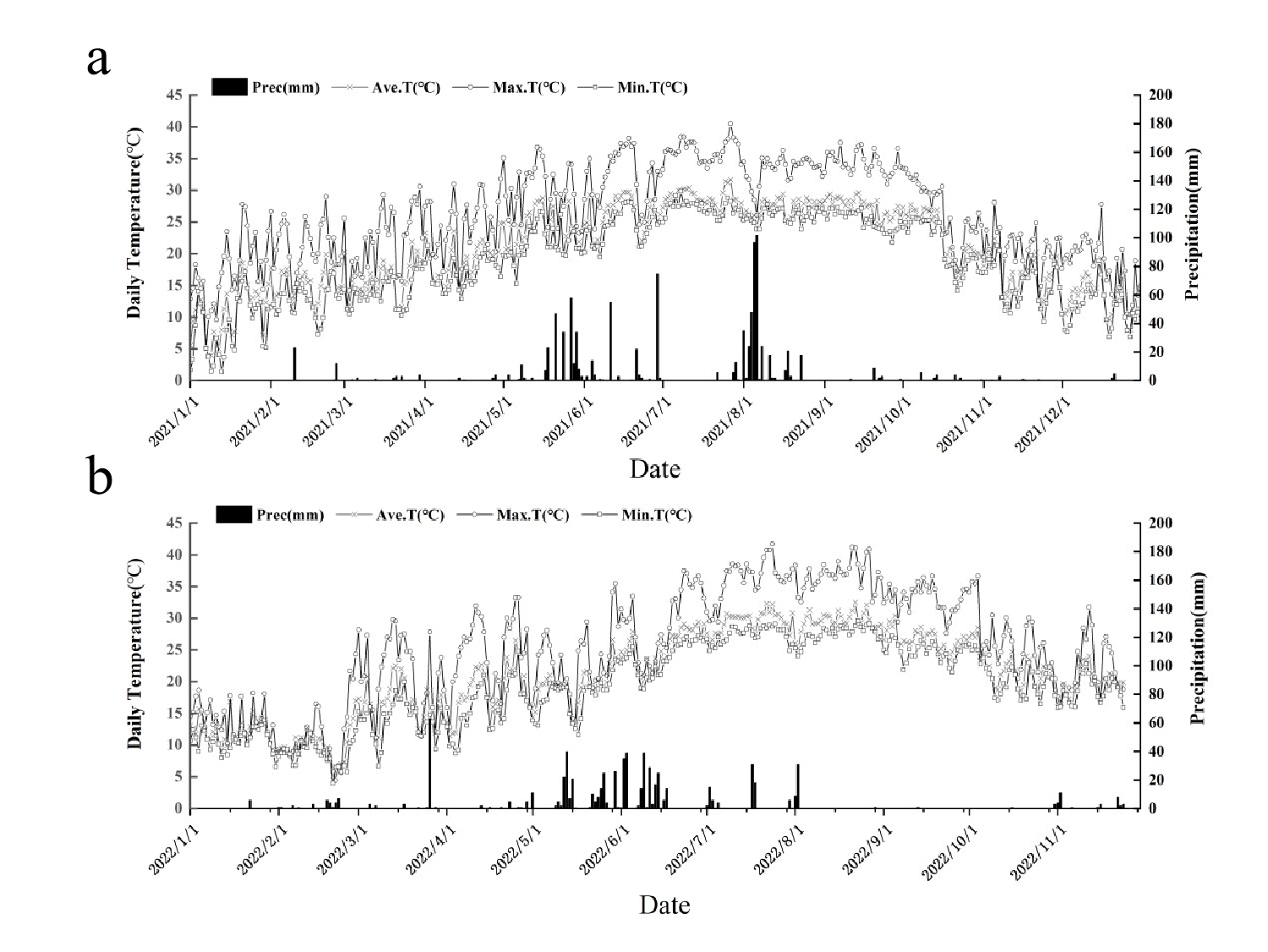


Supplementary Figure 1.a) Changes in rainfall and temperature for different rice varieties, including main crop rice (MC), ratoon season rice (RR), and late season rice (LR) with concurrent anthesis, under various rice cultivation patterns in 2021. b) Changes in rainfall and temperature for different rice varieties, including MC, RR, and LR with concurrent anthesis, under various rice cultivation patterns in 2022.

Supplementary table 1 Constructing vector primers

| Gene |  | Primer（5’-3’） | Intended Use |
| --- | --- | --- | --- |
| *OsSWEET1a* | F | TTCTGTTCCAGGGGCCCCTGGGATCCatggagcacatcgcgaggttc | Construction of Overexpression Vectors |
|  | R | CGATGCGGCCGCTCGAGTCGACCCGGGcacctggctggcgaccttgtcg |  |
| *OsCIPK2* | F | TCCAGCTCCAGGATCCATGGCGGAGCAGAGAGGAAATAT | Construction of Overexpression Vectors |
|  | R | GAGAAAGCTTGGATCCTTAGCACGTTGGCTGCTGCTG |  |
| *OsCIPK2* | F | ggcaGTTACGGGCATGGTAAACCT | Construction of Knockout Vectors |
|  | R | aaacAGGTTTACCATGCCCGTAAC |  |


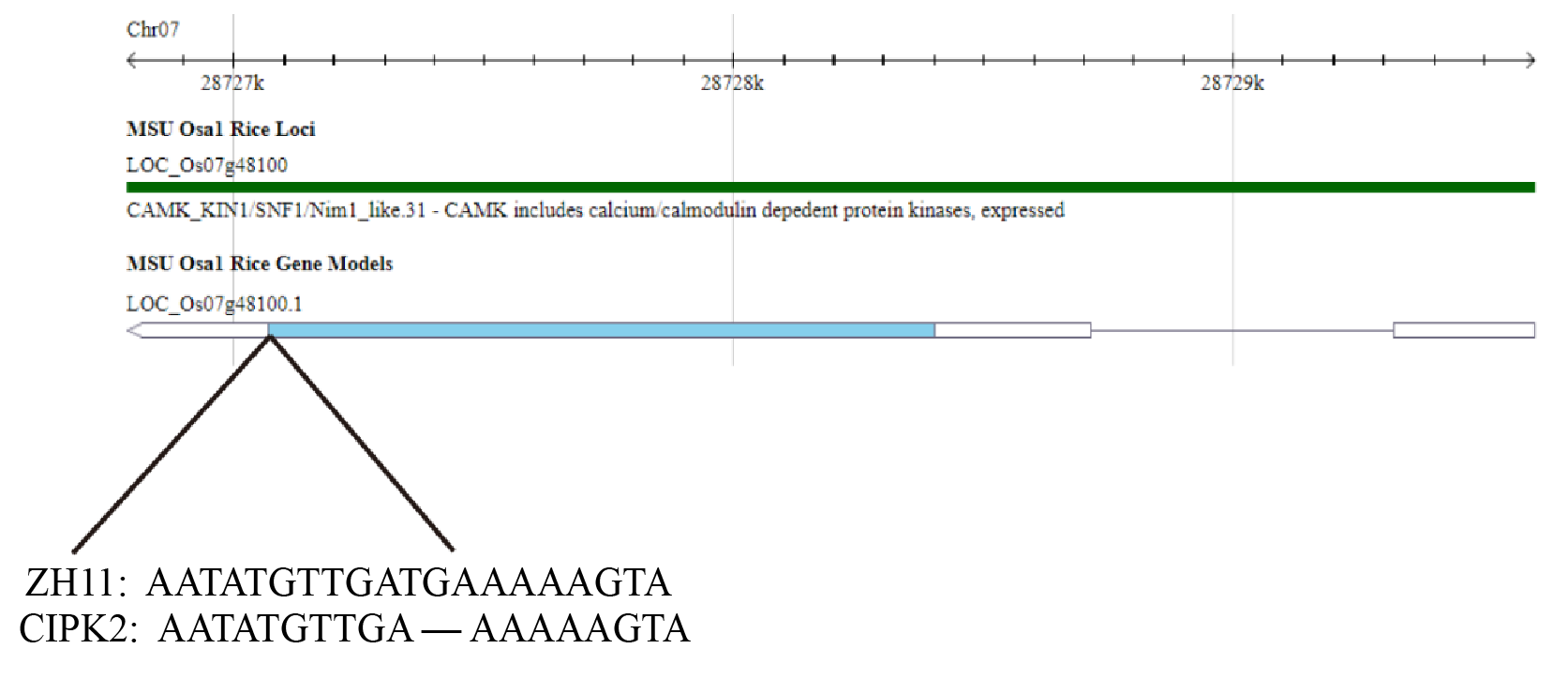


Supplementary Figure 2. Analysis of mutation sites in wild-type ZH11 and mutant OsCIPK2.Note: ZH11 represents the wild-type ZH11, and CIPK2 represents the mutant OsCIPK2.


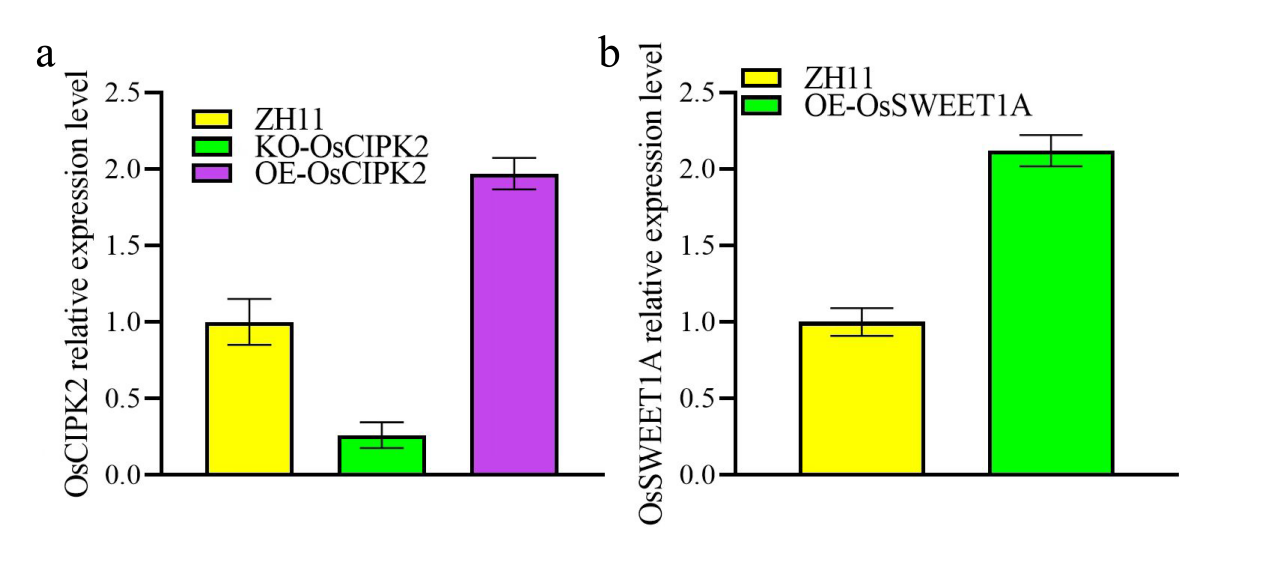


Supplementary Figure 3. Relative expression levels of OsCIPK2 and OsSWEET1A in wild-type Zhonghua 11 and CIPK2 overexpression/mutant lines. a) The impact on the expression levels of wild-type ZH11, OsCIPK2 overexpression, and OsCIPK2 mutant lines; b) The impact on the expression levels of wild-type ZH11 and OsSWEET1A overexpression. Data are presented as mean ± SD (n=3 independent replicates). Source data values are provided in the source data file.

Supplementary table 2 The primer for PCR

| Gene |  | Primer（5’-3’） | Accession NO |
| --- | --- | --- | --- |
| *OsSUT4* | F | CGTGAGTGCGTGATTTGTGT | LOC_Os02g58080 |
|  | R | CGTGGTGTGCTTGTGTGTTT |  |
| *OsSWEET2b* | F | ATCCCGCATCCAACAACCAT | LOC_Os01g50460 |
|  | R | GATGAGGCAATTCAGCAGCG |  |
| *OsSWEET13* | F | TCAGAGCTCTCCCTTCCCTC | LOC_Os12g29220 |
|  | R | GGACAGGCCAGCCATTTTTG |  |
| *OsCIPK28* | F | GTTGGCCTCATGGAGCAGAT | LOC_Os05g39870 |
|  | R | TGCTCTGGCCATTTTGTGGA |  |
| *OsSWEET1a* | F | TCGGGAGAGCTAGAAGCGAT | LOC_Os01g65880 |
|  | R | TCCAGAAGGTGACAACTGGC |  |
| *OsSUT5* | F | GCCGTGTTGTTCAGTGTTCC | LOC_Os02g36700 |
|  | R | GATCACCAGCTGTGGGACAA |  |
| *OsCIPK23* | F | TGACTGATGCAAACCTGAGCA | LOC_Os07g05620 |
|  | R | AGGCCAGGCAGACTCATCTA |  |
| *OsSWEET14* | F | ACATGGGTCGCCGTAAGAAG | LOC_Os11g31190 |
|  | R | AGCATGTCTGCAGCTTCACT |  |
| *OsCIPK2* | F | GAGCACGTCAAAGGTGGAGA | LOC_Os07g48100 |
|  | R | CGGTGATAAACACCCCTGCT |  |
| *OsCIPK3* | F | AGCAACTGATCAACGCCGTA | LOC_Os07g48760 |
|  | R | ACTTTGATGTTCCCGGCAGT |  |
| *OsCIPK18* | F | ATCGCGATCTGAAGCCAGAG | LOC_Os05g26820 |
|  | R | CCGTCATACCCTTTTCGGCT |  |


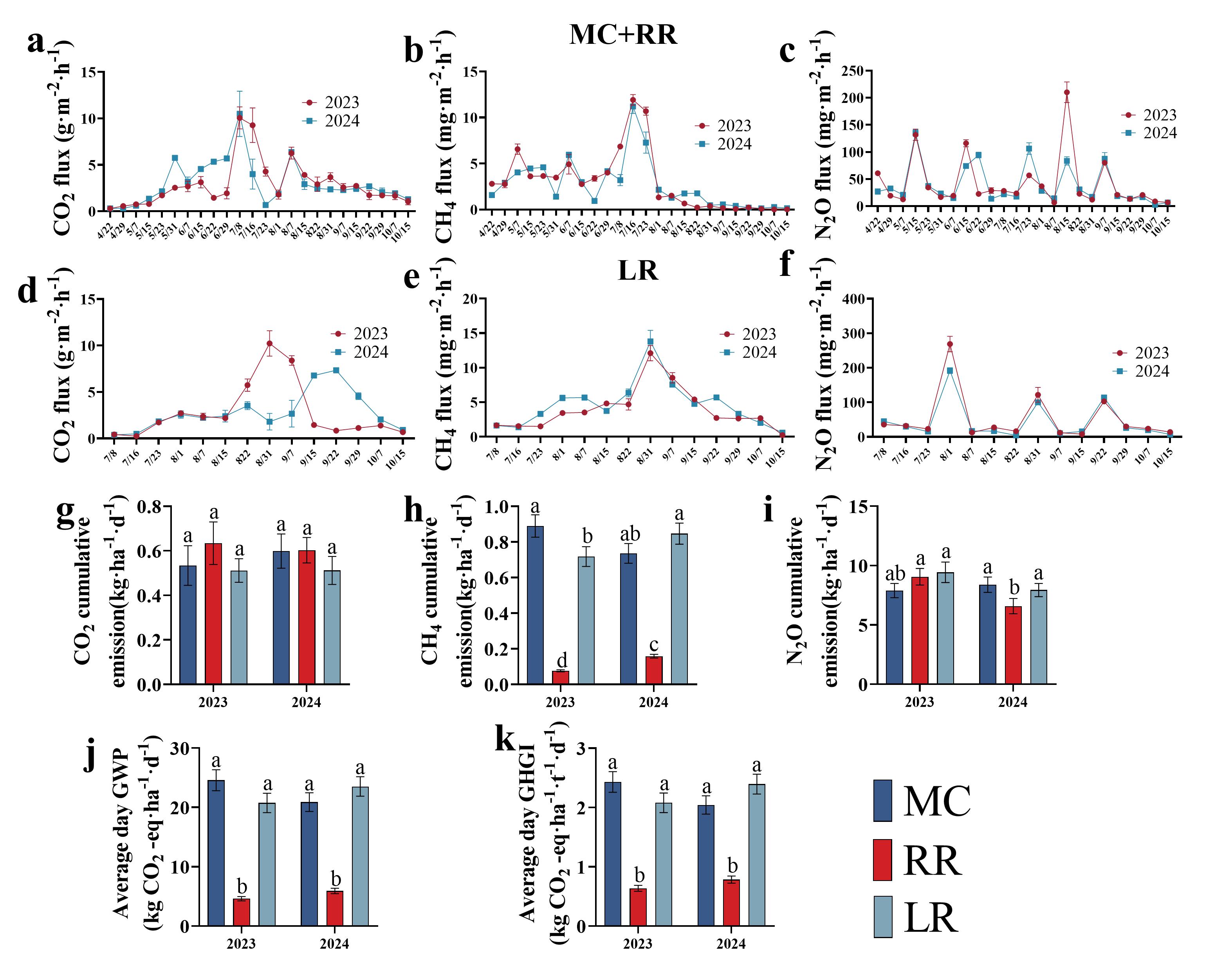


Supplementary Figure 4. Greenhouse gas emissions and daily average greenhouse gas emissions of RR, MC, and LR of YX1803 during the same period. a) CO_2_ emission flux for YX1803 MC and RR in 2023 and 2024; Means ± SD. (n = 3 biological replicates) b) CH_4_ emission flux for YX1803 MC and RR in 2023 and 2024; Means ± SD. (n = 3 biological replicates). c) N_2_O emission flux for YX1803 MC and RR in 2023 and 2024; Means ± SD. (n = 3 biological replicates). d) CO_2_ emission flux for YX1803 LR in 2023 and 2024; Means ± SD. (n = 3 biological replicates). e) CH_4_ emission flux for YX1803 LR in 2023 and 2024; Means ± SD. (n = 3 biological replicates). f) N_2_O emission flux for YX1803 LR in 2023 and 2024; Means ± SD. (n = 3 biological replicates). g) Daily average CO_2_ emission for YX1803 MC, RR and LR. Means ± SD. (n = 3 biological replicates), the different lowercase letters above the column graphs indicate significant differences by Duncan’s test (*p* < 0.05) and figures were plotted using Origin 2021 software. h) Daily average CH_4_ emission for YX1803 MC, RR and LR. Means ± SD. (n = 3 biological replicates), the different lowercase letters above the column graphs indicate significant differences by Duncan’s test (*p* < 0.05) and figures were plotted using Origin 2021 software. i) Daily average N_2_O emission for YX1803 MC, RR and LR. Means ± SD. (n = 3 biological replicates), the different lowercase letters above the column graphs indicate significant differences by Duncan’s test (*p* < 0.05) and figures were plotted using Origin 2021 software. j) Daily average GWP for YX1803 MC, RR and LR. Means ± SD. (n = 3 biological replicates), the different lowercase letters above the column graphs indicate significant differences by Duncan’s test (*p* < 0.05) and figures were plotted using Origin 2021 software. k) Daily average GHGI for YX1803 MC, RR and LR. Means ± SD. (n = 3 biological replicates), the different lowercase letters above the column graphs indicate significant differences by Duncan’s test (*p* < 0.05) and figures were plotted using Origin 2021 software. Figure a-f figures were plotted using Origin 2021 software. Source data are provided in the source data file.


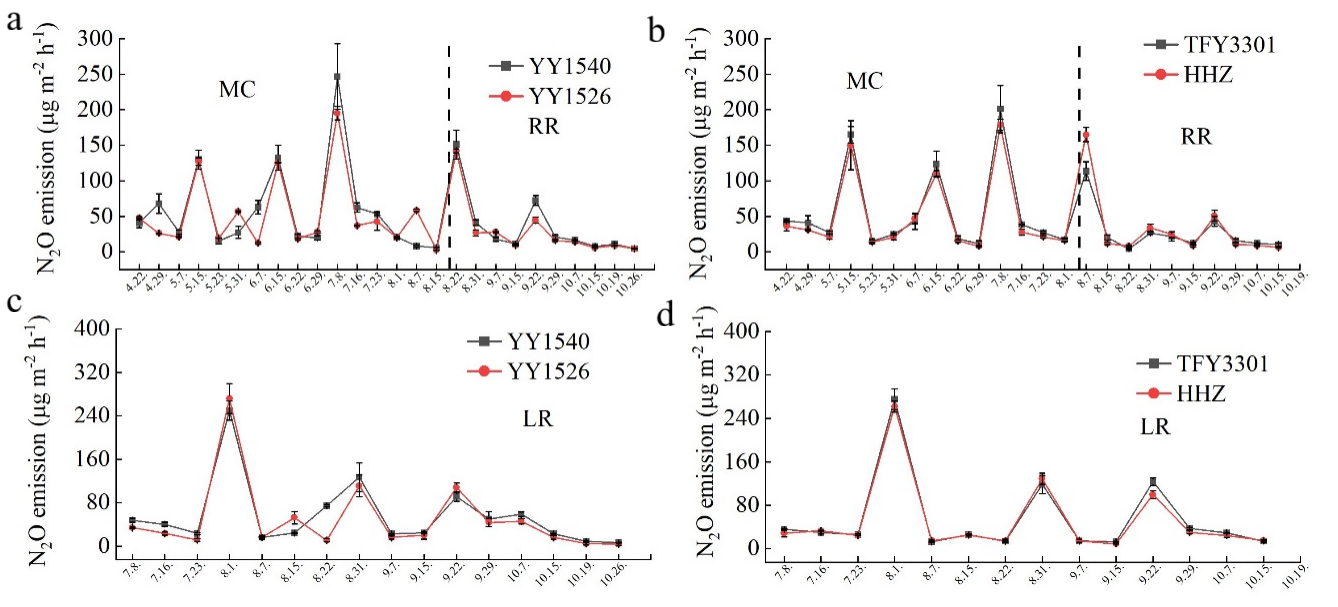


Supplementary Figure 5. N_2_O emission flux of RR, MC, and LR during the same period in 2021. a) N_2_O emission flux for YY1540 and YY1526 MC and RR in 2021; Means ± SD. (n = 3 biological replicates) b) N_2_O emission flux for TFY3301 and HHZ MC and RR in 2021; Means ± SD. (n = 3 biological replicates). c) N_2_O emission flux for YY1540 and YY1526 MC and RR in 2021; Means ± SD. (n = 3 biological replicates). d) N_2_O emission flux for TFY3301 and HHZ MC and RR in 2021; Means ± SD. (n = 3 biological replicates), figures were plotted using Origin 2021 software. Source data are provided in the source data file.


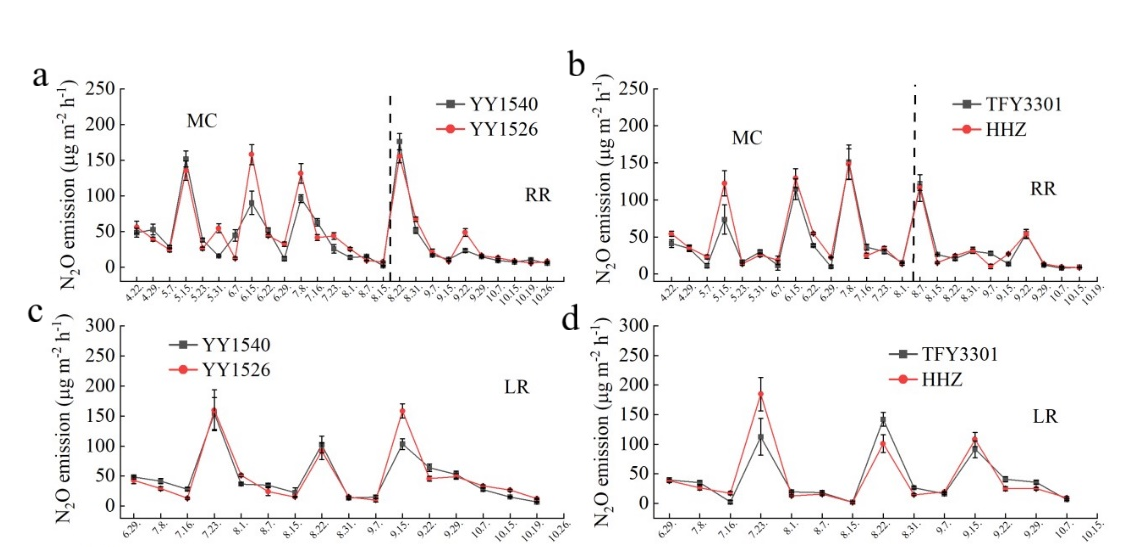


Supplementary Figure 6. N_2_O emission flux of RR, MC, and LR during the same period in 2022. a) N_2_O emission flux for YY1540 and YY1526 MC and RR in 2022; Means ± SD. (n = 3 biological replicates) b) N_2_O emission flux for TFY3301 and HHZ MC and RR in 2022; Means ± SD. (n = 3 biological replicates). c) N_2_O emission flux for YY1540 and YY1526 MC and RR in 2022; Means ± SD. (n = 3 biological replicates). d) N_2_O emission flux for TFY3301 and HHZ MC and RR in 2022; Means ± SD. (n = 3 biological replicates), figures were plotted using Origin 2021 software. Source data are provided in the source data file.


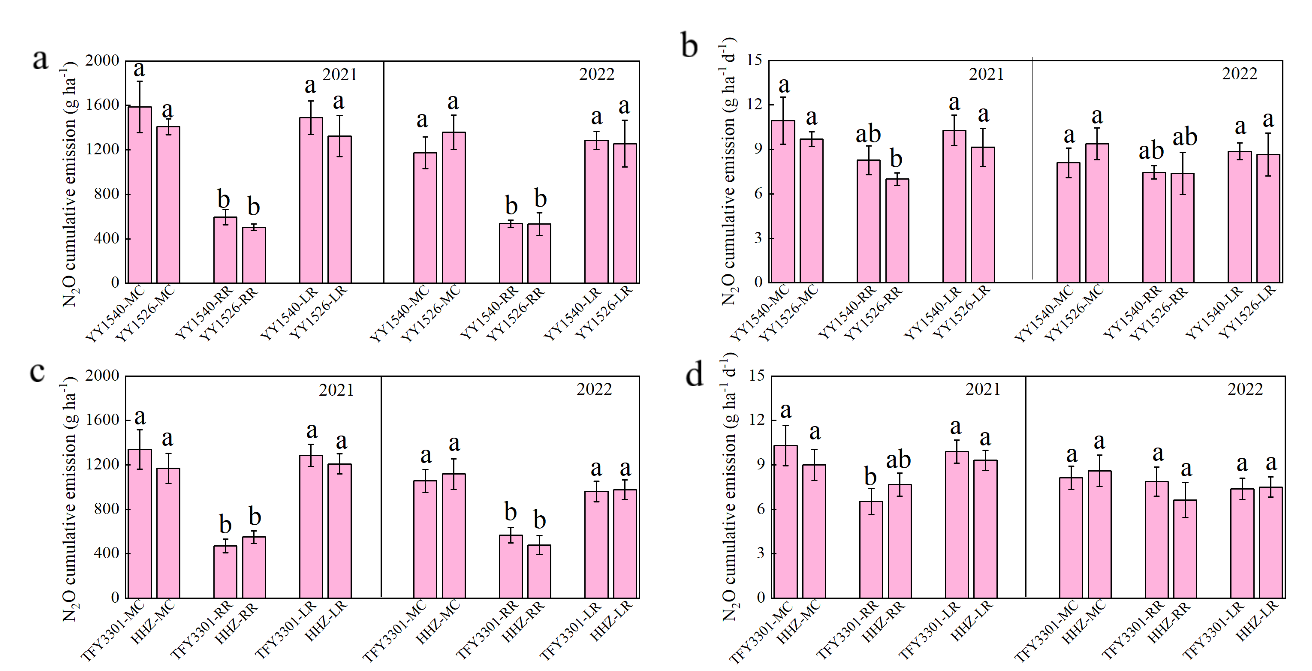


Supplementary Figure 7. Cumulative and daily average N_2_O emissions of RR, MC, and LR during the same period. a) Cumulative N_2_O emissions YY1540 and YY1526 MC, RR and LR. Means ± SD. (n = 3 biological replicates), the different lowercase letters above the column graphs indicate significant differences by Duncan’s test (*p* < 0.05) and figures were plotted using Origin 2021 software. b) Daily N_2_O emissions YY1540 and YY1526 MC, RR and LR. Means ± SD. (n = 3 biological replicates), the different lowercase letters above the column graphs indicate significant differences by Duncan’s test (*p* < 0.05) and figures were plotted using Origin 2021 software. c)Cumulative N_2_O emissions TFY3301 and HHZ MC, RR and LR. Means ± SD. (n = 3 biological replicates), the different lowercase letters above the column graphs indicate significant differences by Duncan’s test (*p* < 0.05) and figures were plotted using Origin 2021 software. d)Daily N_2_O emissions TFY3301 and HHZ MC, RR and LR. Means ± SD. (n = 3 biological replicates), the different lowercase letters above the column graphs indicate significant differences by Duncan’s test (*p* < 0.05) and figures were plotted using Origin 2021 software. Source data are provided in the source data file.


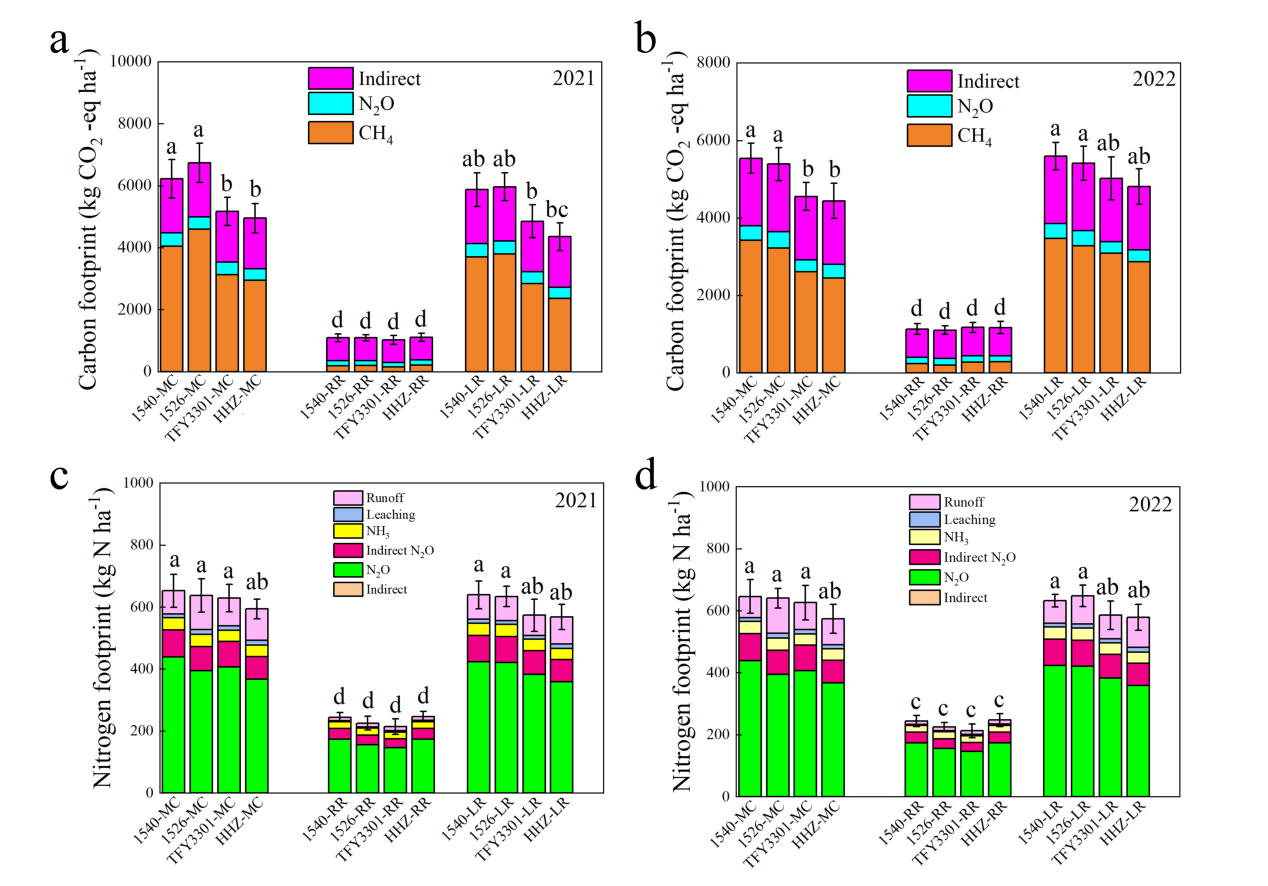


Supplementary Figure 8. Carbon and nitrogen footprint of RR, MC, and LR with heading at the same time. a) Carbon footprint for different varieties of MC, RR, and LR in 2021. Means ± SD. (n = 3 biological replicates), the different lowercase letters above the column graphs indicate significant differences by Duncan’s test (*p* < 0.05) and figures were plotted using Origin 2021 software. b) Carbon footprint for different varieties of MC, RR, and LR in 2022. Means ± SD. (n = 3 biological replicates), the different lowercase letters above the column graphs indicate significant differences by Duncan’s test (*p* < 0.05) and figures were plotted using Origin 2021 software. c)Nitrogen footprint for different varieties of MC, RR, and LR in 2021. Means ± SD. (n = 3 biological replicates), the different lowercase letters above the column graphs indicate significant differences by Duncan’s test (*p* < 0.05) and figures were plotted using Origin 2021 software. d)Nitrogen footprint for different varieties of MC, RR, and LR in 2022. Means ± SD. (n = 3 biological replicates), the different lowercase letters above the column graphs indicate significant differences by Duncan’s test (*p* < 0.05) and figures were plotted using Origin 2021 software. Source data are provided in the source data file.


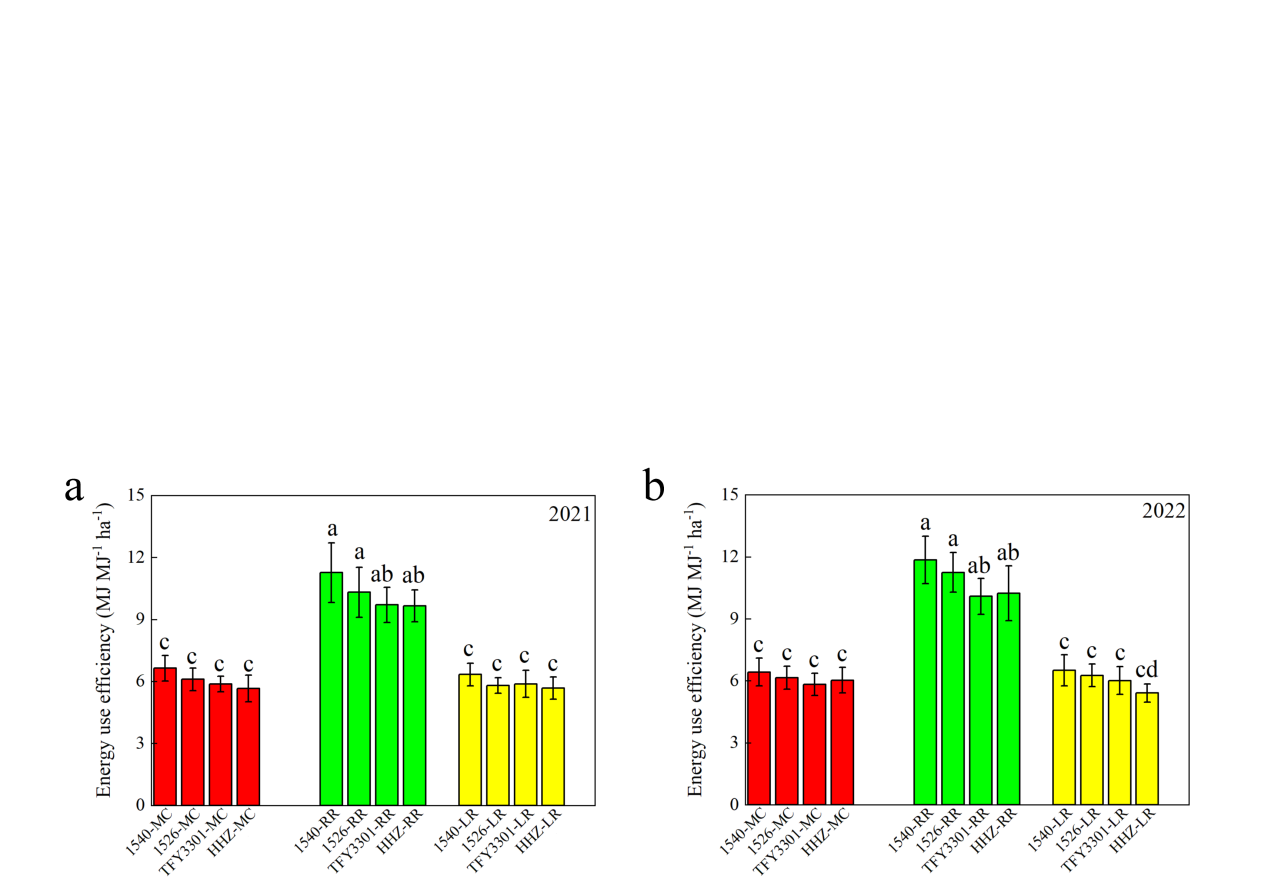


Supplementary Figure 9. Resource utilization efficiency of RR, MC, and LR during the same period. a) Resource utilization efficiency for different varieties of MC, RR, and LR in 2021. Means ± SD. (n = 3 biological replicates), the different lowercase letters above the column graphs indicate significant differences by Duncan’s test (*p* < 0.05) and figures were plotted using Origin 2021 software.; b) Resource utilization efficiency for different varieties of MC, RR and LR in 2022. Means ± SD. (n = 3 biological replicates), the different lowercase letters above the column graphs indicate significant differences by Duncan’s test (*p* < 0.05) and figures were plotted using Origin 2021 software. Source data are provided in the source data file.


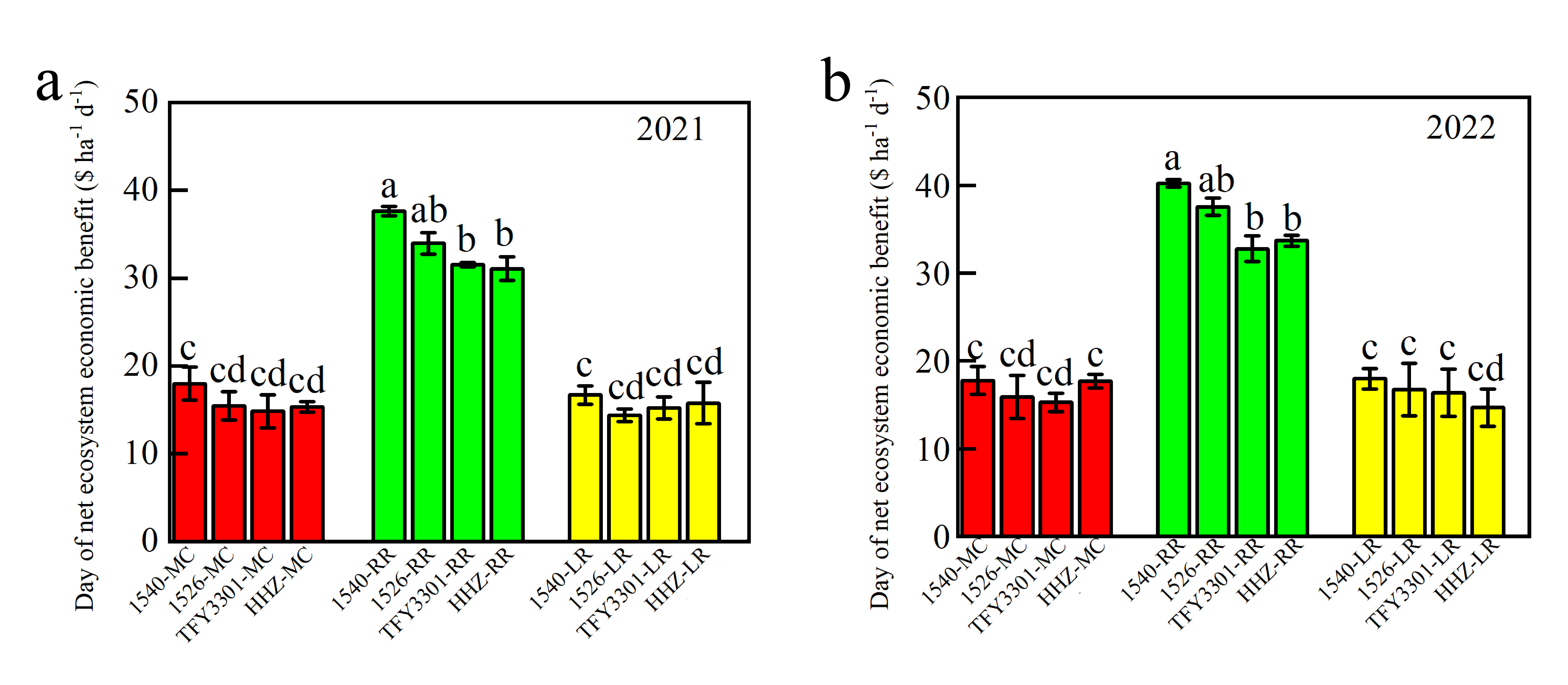


Supplementary Figure 10. Day of net ecosystem economic benefits of RR, MC, and LR during the same period from 2021 to 2022. a) Day of net ecosystem economic benefits of MC, RR, and LR in 2021. Means ± SD. (n = 3 biological replicates), the different lowercase letters above the column graphs indicate significant differences by Duncan’s test (*p* < 0.05) and figures were plotted using Origin 2021 software.; b) Day of net ecosystem economic benefits for different varieties of MC, RR and LR in 2022. Means ± SD. (n = 3 biological replicates), the different lowercase letters above the column graphs indicate significant differences by Duncan’s test (*p* < 0.05) and figures were plotted using Origin 2021 software. Source data are provided in the source data file.

Supplementary table 3. Comparison of yield among different varieties of RR, MC, and LR during the same period in 2021.

|  | Variety | Panicles m^-2^ | Spikelets per panicle | Grain filling rate  (%) | 1000-grain weight  (g) | Grain yield  (t ha^−1^) | Yield per day  (kg ha^-1^ d^-1^) |
| --- | --- | --- | --- | --- | --- | --- | --- |
| MC | YY1540 | 279.55b | 232.81a | 80.58 ab | 22.50 b | 11.79a | 81.37c |
|  | 1526 | 268.22b | 220.51ab | 81.81ab | 22.42b | 10.84ab | 74.81d |
|  | TFY3301 | 252.97b | 207.33 b | 82.63 ab | 22.61 b | 9.79b | 75.37d |
|  | HHZ | 247.89b | 216.74 ab | 78.34 b | 22.44b | 9.44b | 72.65de |
| RR | YY1540 | 372.88a | 104.24 c | 87.95 a | 24.75a | 8.46bc | 117.51a |
|  | GYF | 380.21a | 100.69 c | 85.75 a | 23.61ab | 7.75c | 107.64ab |
|  | TFY3301 | 370.24a | 95.46 c | 88.33 a | 23.38 ab | 7.29c | 101.37ab |
|  | HHZ | 375.80a | 93.85 c | 86.21a | 23.89 ab | 7.26c | 100.88ab |
| LR | YY1540 | 264.55b | 219.54 ab | 85.58 a | 22.64 b | 11.25a | 77.60d |
|  | YY1526 | 268.25b | 225.41 ab | 80.81ab | 21.10bc | 10.31ab | 71.10de |
|  | TFY3301 | 259.37b | 213.72 ab | 78.34 b | 22.61 b | 9.81b | 75.52d |
|  | HHZ | 245.45b | 204.15 b | 82.79 ab | 22.83 b | 9.47b | 72.85de |

Note: In YY1540, YY1526, TFY3301, and HHZ varieties, MC refers to the main crop rice, RR refers to the ratoon season rice, LR refers to the (late rice) with the heading stage synchronized to that of the RR. Data are presented as n=20 independent replicates, values with a column followed by different letters are significantly different by Duncan’s test (*p* < 0.05).

Supplementary table 4. Comparison of yield among different varieties of RR, MC, and LR during the same period in 2022.

|  | Variety | Panicles m^-2^ | Spikelets per panicle | Grain filling rate  (%) | 1000-grain weight  (g) | Grain yield  (t ha^−1^) | Yield per day  (kg ha^-1^ d^-1^) |
| --- | --- | --- | --- | --- | --- | --- | --- |
| MC | YY1540 | 306.65c | 197.20b | 78.76c | 23.98a | 11.42a | 78.76c |
|  | 1526 | 308.61c | 195.56b | 78.78c | 23.00b | 10.93a | 75.41c |
|  | TFY3301 | 282.38c | 187.63c | 90.14a | 22.93b | 9.73b | 74.89c |
|  | HHZ | 287.82c | 186.52c | 79.45c | 23.46a | 10.06b | 76.97c |
| RR | YY1540 | 423.16a | 98.69d | 85.98b | 24.79a | 8.90c | 123.62a |
|  | GYF | 400.23ab | 101.36d | 87.27a | 23.88a | 8.45d | 117.42ab |
|  | TFY3301 | 393.64ab | 96.37d | 84.05b | 23.79a | 7.58e | 105.35b |
|  | HHZ | 385.53ab | 98.43d | 85.14b | 23.81a | 7.69e | 106.84b |
| LR | YY1540 | 285.42d | 213.82a | 83.46bc | 22.73b | 11.57a | 79.84c |
|  | YY1526 | 293.31d | 195.62b | 82.57bc | 23.51a | 11.13a | 76.81c |
|  | TFY3301 | 269.25d | 203.87ab | 78.98c | 23.14a | 10.03b | 77.16c |
|  | HHZ | 265.56d | 184.54c | 82.61d | 22.32b | 9.03c | 69.50d |

Note: In YY1540, YY1526, TFY3301, and HHZ varieties, MC refers to the main crop rice, RR refers to the ratoon season rice, LR refers to the (late rice) with the heading stage synchronized to that of the RR. Data are presented as n=20 independent replicates, values with a column followed by different letters are significantly different by Duncan’s test (*p* < 0.05).

Supplementary table 5. Comparison of yield and its component among YX1803 of the MC, the RR, and the LR in 2023-2024

| Year | Treatment | Panicle (plant) | Spikelets per panicle | 1000-grain  weight (g) | Grain filling rate  (%) | Yield  (t ha^-1^) |
| --- | --- | --- | --- | --- | --- | --- |
| 2023 | MC | 18.23b | 213.08a | 22.47a | 79.23b | 10.12a |
|  | RR | 31.53a | 122.74b | 23.98a | 88.36a | 7.24b |
|  | LR | 17.42b | 198.36a | 21.98a | 77.12b | 9.98a |
| 2024 | MC | 18.31b | 223.86a | 22.56a | 77.63b | 10.23a |
|  | RR | 33.01a | 130.23b | 23.98a | 88.12a | 7.56b |
|  | LR | 17.09b | 202.52a | 22.04a | 81.42b | 9.83a |

Note: MC refers to the main crop rice, RR refers to the ratoon season rice, LR refers to the (late rice) with the heading stage synchronized to that of the RR. Data are presented as n=20 independent replicates, values with a column followed by different letters are significantly different by Duncan’s test (*p* < 0.05).


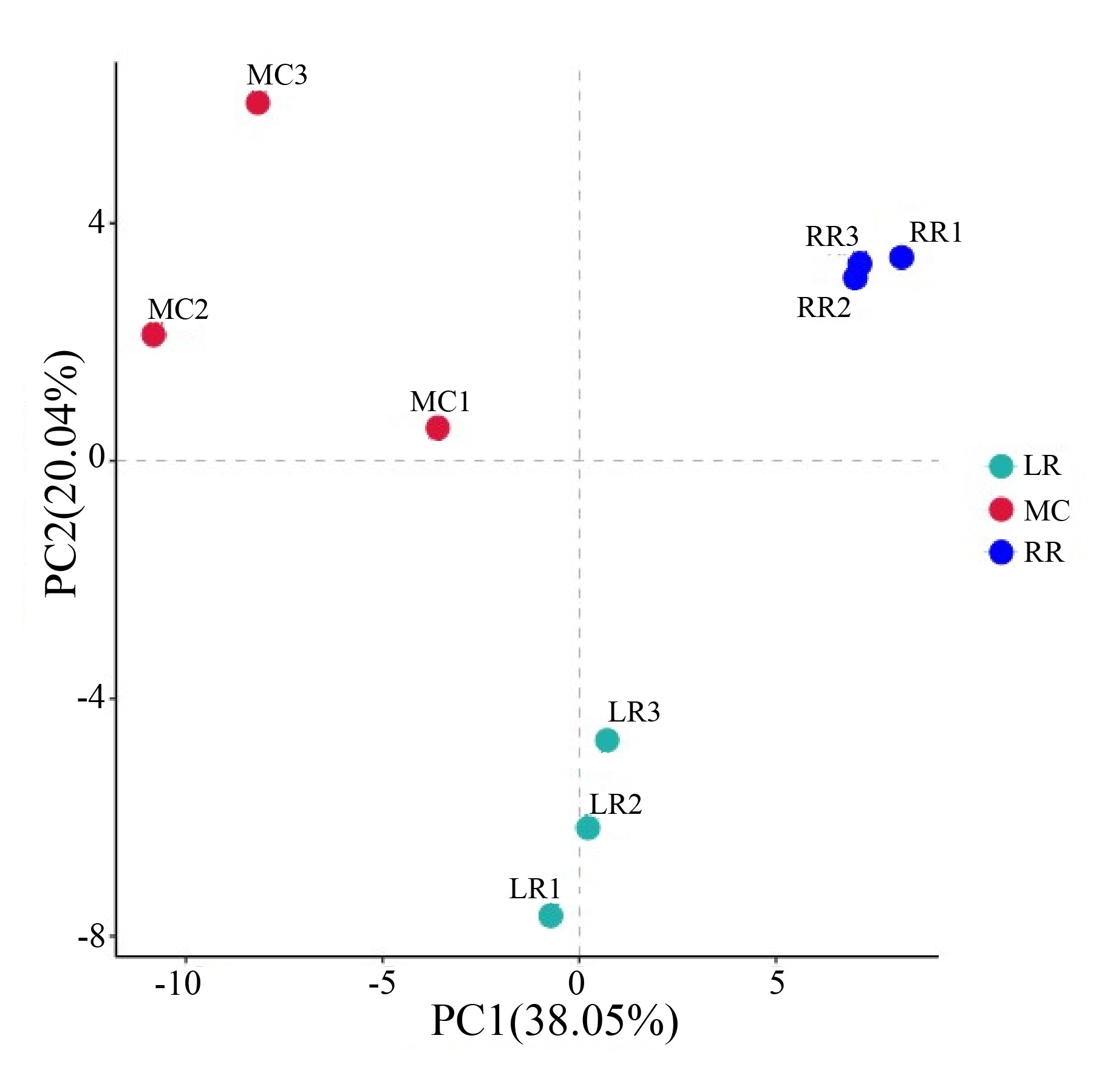


Supplementary Figure 11. Changes in Root exudates Metabolic PCA under Different Planting Patterns. Graphics are all created using R software version 4.4.1. Source data are provided in the source data file.


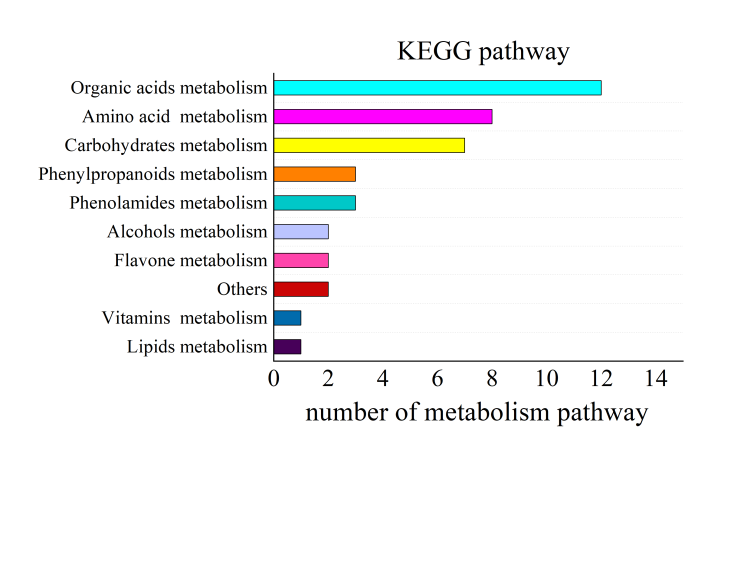


Supplementary Figure 12. Changes in Root exudates Metabolic KEGG under Different Planting Patterns. Graphics are all created using R software version 4.4.1. Source data are provided in the source data file.


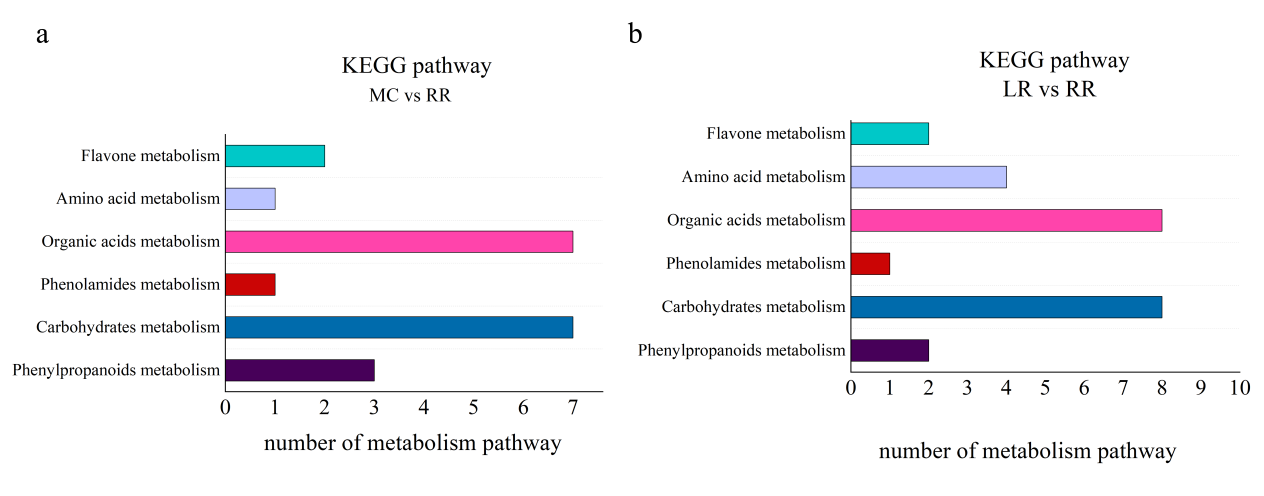


Supplementary Figure 13. Differences in KEGG Metabolism in Root exudates Metabolic under Different Planting Patterns. a) Comparison of differential KEGG metabolic pathways in root exudate metabolism between MC and RR under different planting patterns; b) Comparison of differential KEGG metabolic pathways in root exudate metabolism between LR and RR under different planting patterns. Graphics are all created using R software version 4.4.1. Source data are provided in the source data file.


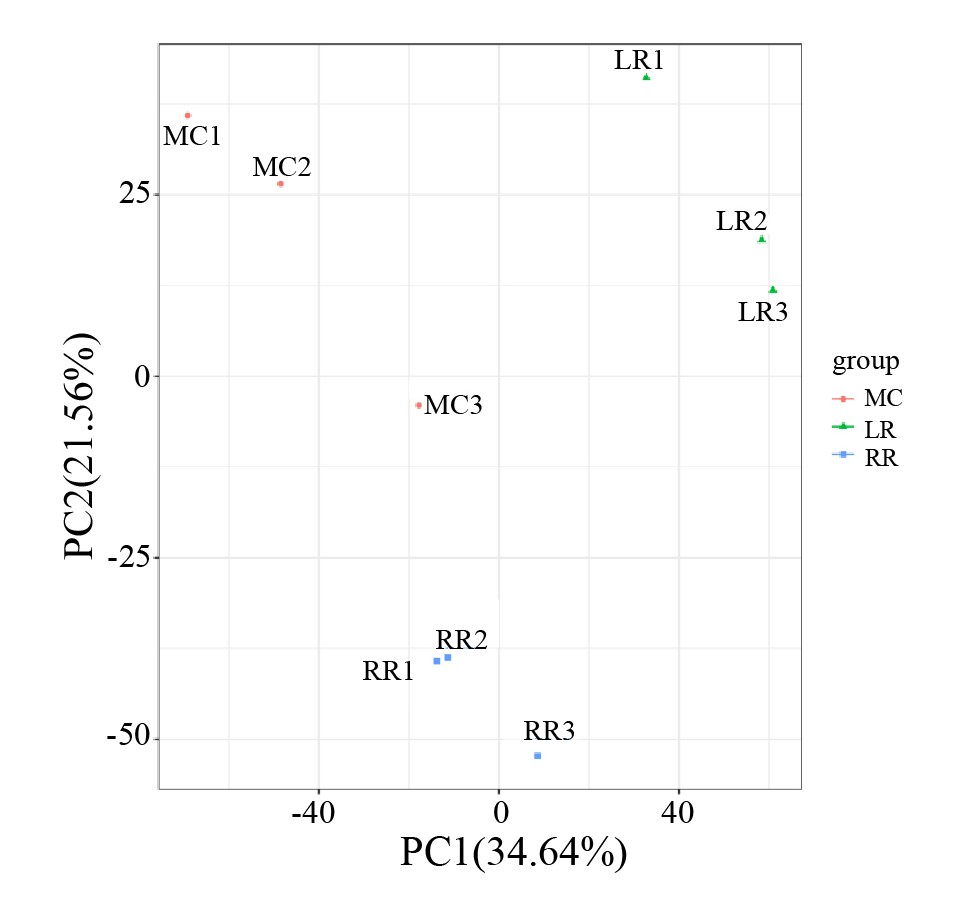


Supplementary Figure 14. Changes in Soil Metabolic PCA under Different Planting Patterns. Note: MC refers to YY1540 main crop rice, RR refers to YY1540 ratoon season rice, and LR refers to YY1540 (late season) synchronized in rice heading time. Graphics are all created using R software version 4.4.1. Source data are provided in the source data file.


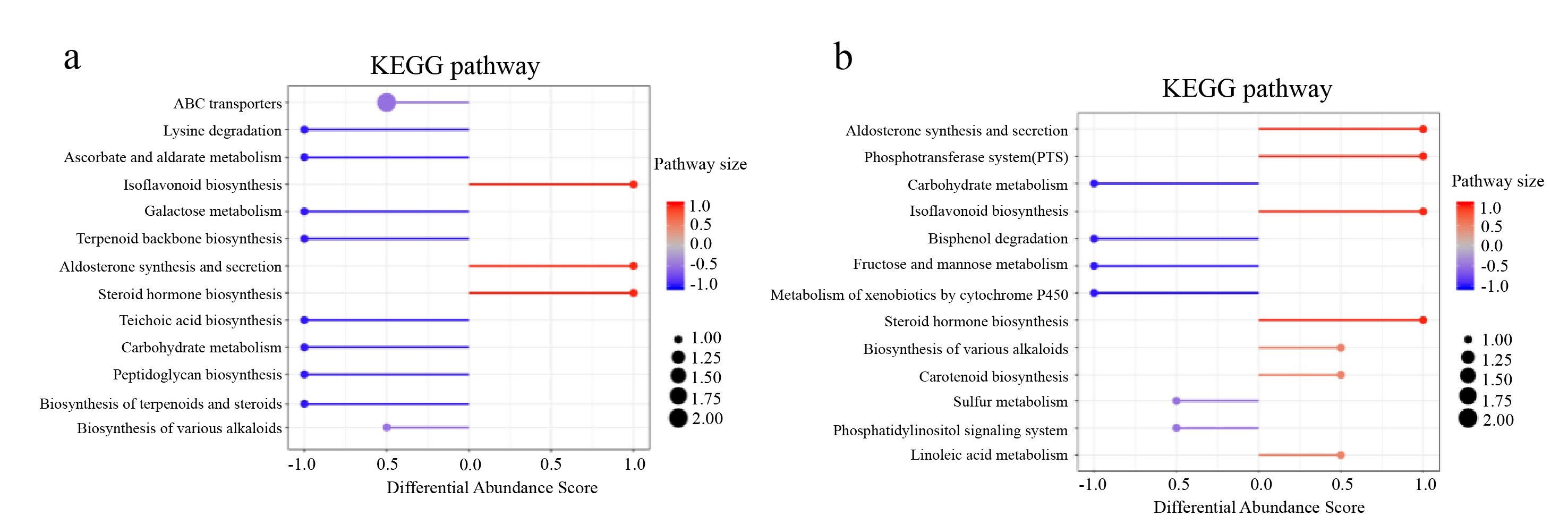


Supplementary Figure 15. Differences in KEGG Metabolism in Soil under Different Planting Patterns. a) Comparison of KEGG metabolic pathways in rhizosphere soil of MC and RR under different planting patterns; b) Comparison of KEGG metabolic pathways in rhizosphere soil of LR and RR under different planting patterns. Graphics are all created using R software version 4.4.1. Source data are provided in the source data file.


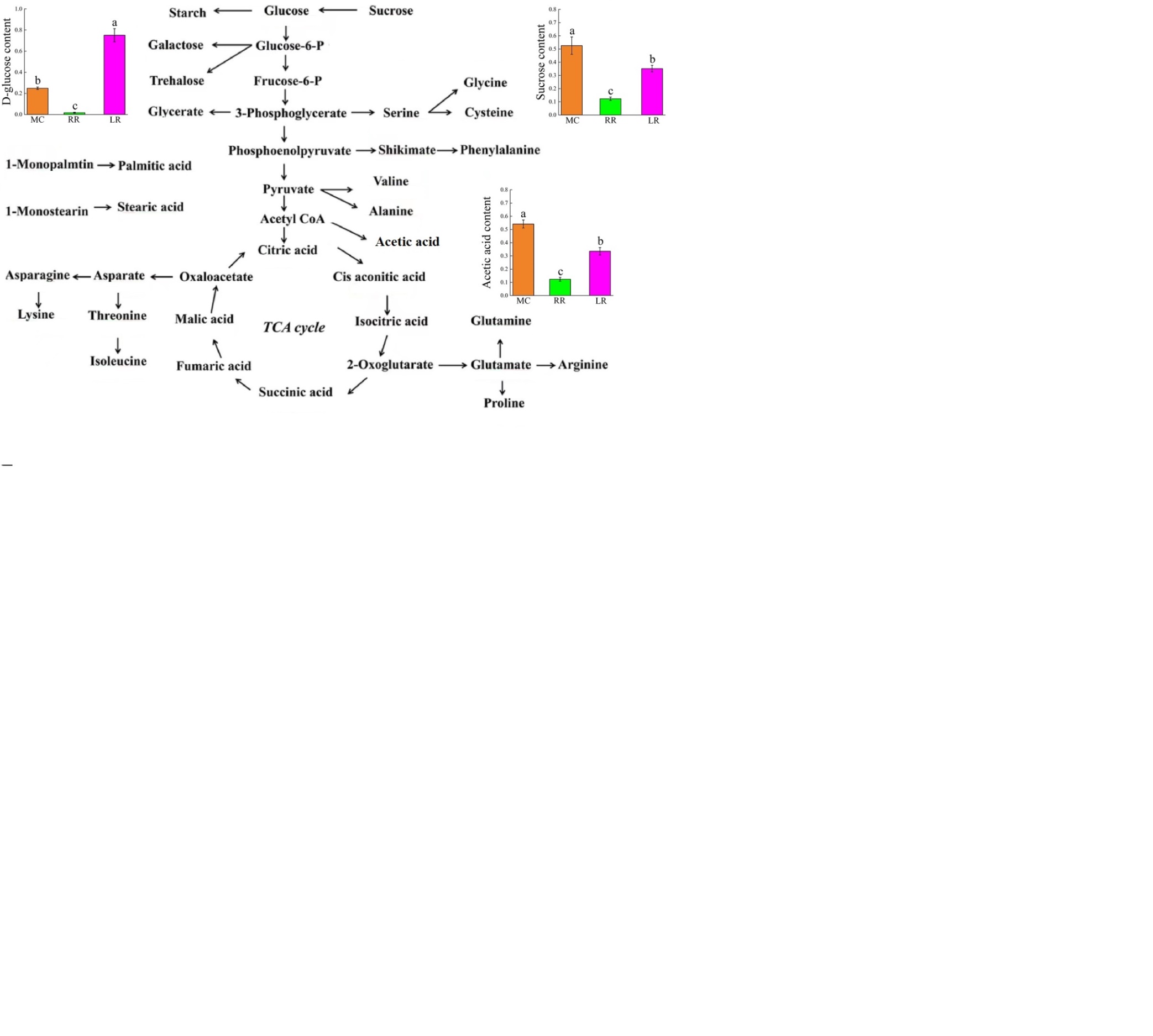


Supplementary Figure 16. Differences in soil metabolic pathways rice during heading stage. D-glucose, Sucrose, Acetic content differences in metabolism in soil under different planting patterns. Means ± SD. (n = 3 biological replicates), the different lowercase letters above the column graphs indicate significant differences by Duncan’s test (*p* < 0.05) and figures were plotted using Origin 2021 software.


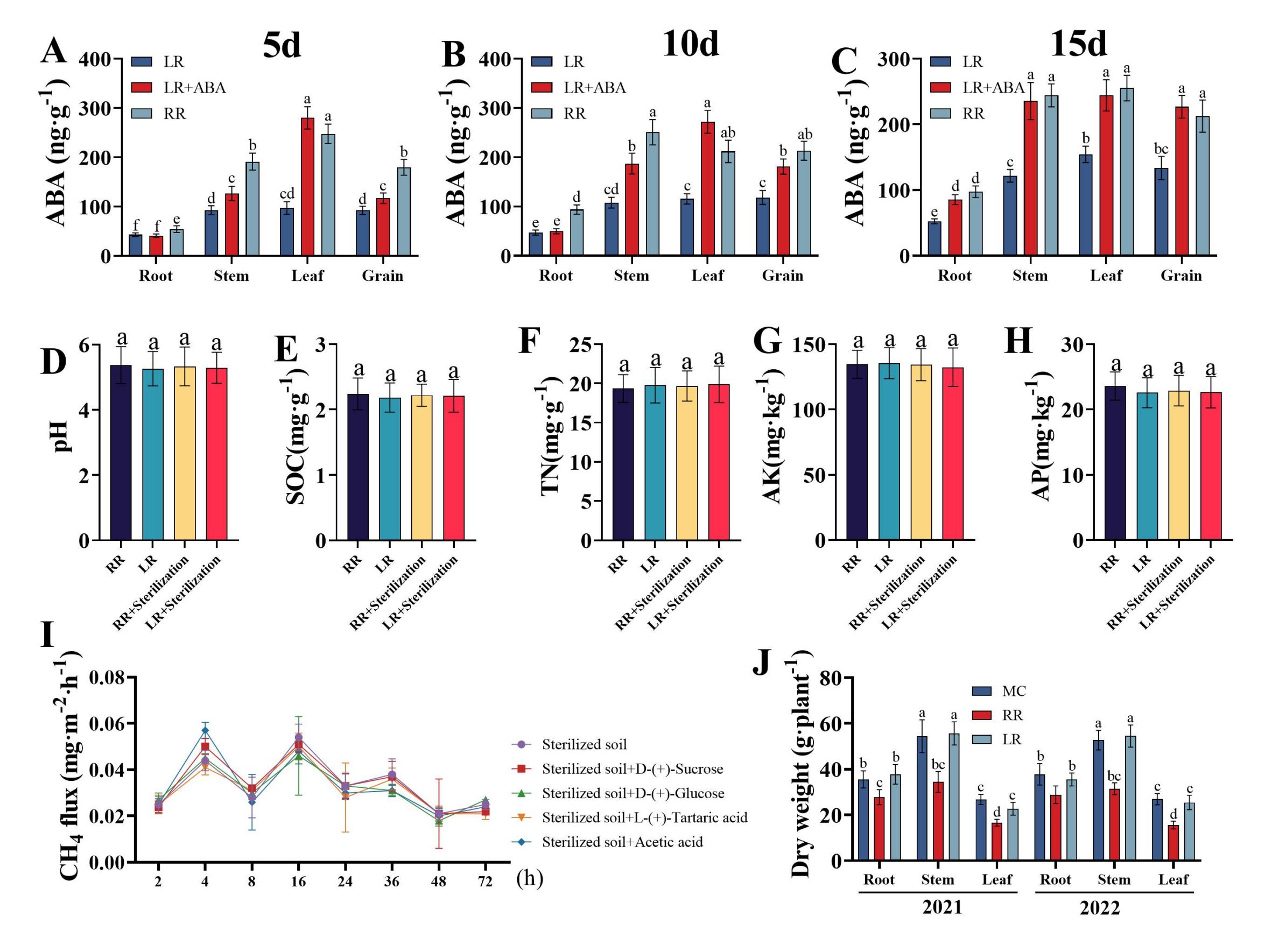


Supplementary Figure 17. CH_4_ emission flux of YY1540 ratoon season rice after adding different metabolites under soil sterilization conditions. Means ± SD. (n = 3 biological replicates), figures were plotted using Origin 2021 software. Source data are provided in the source data file.


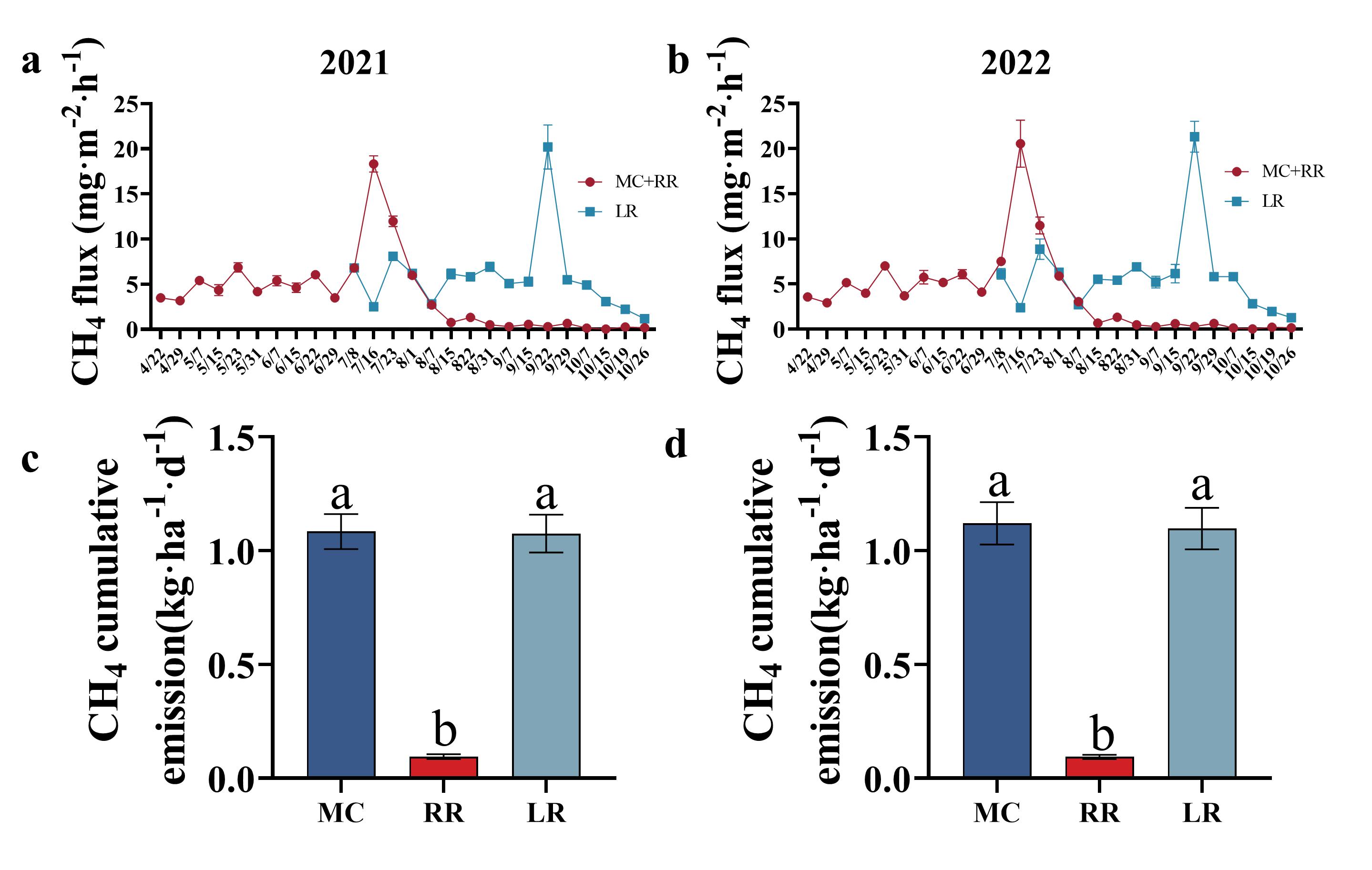


Supplementary Figure 18. CH_4_ flux emissions and daily average CH_4_ emissions of RR, MC, and LR of YY1540 in pot during the same period. a) CH_4_ emission flux for MC and RR in 2021; Means ± SD. (n = 3 biological replicates), figures were plotted using Origin 2021 software. b) CH_4_ emission flux for MC and RR in 2022; Means ± SD. (n = 3 biological replicates), figures were plotted using Origin 2021 software. c) Daily average CH_4_ emissions for MC and RR in 2021. Means ± SD. (n = 3 biological replicates), the different lowercase letters above the column graphs indicate significant differences by Duncan’s test (*p* < 0.05) and figures were plotted using Origin 2021 software. d) Daily average CH_4_ emissions for MC and RR in 2022. Means ± SD. (n = 3 biological replicates), the different lowercase letters above the column graphs indicate significant differences by Duncan’s test (*p* < 0.05) and figures were plotted using Origin 2021 software. Source data are provided in the source data file.

Supplementary table 6. Comparison of yield and its component among YY1540 of the MC, the RR, and the LR in 2021-2022.

| Year | Treatment | Panicle  Number(plant) | Spikelets per panicle | 1000-grain  weight (g) | Grain filling rate (%) | Yield  (t ha^-1^) |
| --- | --- | --- | --- | --- | --- | --- |
| 2021 | MC | 18.66b | 254.33a | 21.26b | 75.42c | 10.33a |
|  | RR | 35.53a | 183.38b | 23.55a | 85.54a | 7.16b |
|  | LR | 17.75b | 246.75a | 21.42b | 74.85c | 9.95a |
| 2022 | MC | 18.75b | 244.62a | 21.38b | 73.29c | 10.65a |
|  | RR | 33.38a | 182.67b | 23.44a | 85.52a | 7.22b |
|  | LR | 18.21b | 249.62a | 21.69b | 78.62b | 10.38a |

Note: MC refers to the main crop rice, RR refers to the ratoon season rice, LR refers to the (late rice) with the heading stage synchronized to that of the RR. Data are presented as n=20 independent replicates, values with a column followed by different letters are significantly different by Duncan’s test (*p* < 0.05).

Supplementary table 7. The NSC (Non-structural Carbohydrates) transport from RR, MC, and LR during the same period in 2021.

| Rice cultivation model | Variety | NSC transport amount g plant^-1^ | | | | NSC transfer rate% | | | | | | NSC contribution rate% | | | | |
| --- | --- | --- | --- | --- | --- | --- | --- | --- | --- | --- | --- | --- | --- | --- | --- | --- |
|  |  | root | stem | leaf | stubble | | root | stem | leaf | stubble | root | | stem | leaf | stubble |  |
| MC | YY1540 | 1.17c | 3.63b | 2.37b |  | | 28.88d | 31.98c | 37.73c |  | 1.93f | | 6.00c | 3.91e |  |  |
|  | YY1526 | 1.11c | 2.26d | 1.58d |  | | 28.38d | 22.26d | 28.46e |  | 1.99f | | 4.06ef | 2.84g |  |  |
|  | TFY3301 | 0.92c | 2.84c | 1.90c |  | | 23.89e | 29.39c | 37.84c |  | 1.83f | | 5.65cd | 3.78e |  |  |
|  | HHZ | 1.12c | 2.75c | 2.16b |  | | 27.51d | 28.35c | 41.37c |  | 2.31e | | 5.68cd | 4.46d |  |  |
| RR | YY1540 | 1.85a | 5.38a | 2.67a | 0.54a | | 55.89ab | 67.92b | 67.93a | 72.00a | 4.26b | | 12.40b | 6.15b | 1.24a |  |
|  | YY1526 | 1.85a | 5.66a | 2.55a | 0.58a | | 55.55ab | 73.69a | 65.89b | 69.88a | 4.65b | | 14.24a | 6.45ab | 1.45a |  |
|  | TFY3301 | 1.90a | 5.40a | 2.46ab | 0.42b | | 58.46a | 73.77a | 69.29a | 65.62b | 5.08a | | 14.44a | 6.58ab | 1.12b |  |
|  | HHZ | 1.96a | 5.40a | 2.68a | 0.39b | | 60.12a | 73.46a | 72.62a | 62.90b | 5.26a | | 14.50a | 7.19a | 1.04b |  |
| LR | YY1540 | 1.61b | 2.57c | 1.94c |  | | 37.61c | 25.12cd | 34.58d |  | 2.79d | | 4.45e | 3.36f |  |  |
|  | YY1526 | 1.59b | 2.24d | 2.21b |  | | 38.68c | 22.35d | 39.18c |  | 3.01c | | 4.23e | 4.17d |  |  |
|  | TFY3301 | 0.86cd | 2.61c | 1.86c |  | | 21.55e | 27.30c | 37.05c |  | 1.71f | | 5.18d | 3.69e |  |  |
|  | HHZ | 0.90c | 2.17d | 1.89c |  | | 22.78e | 24.05cd | 36.98c |  | 1.85f | | 4.46e | 3.89e |  |  |

Note: In YY1540, YY1526, TFY3301, and HHZ varieties, MC refers to the main crop rice, RR refers to the ratoon season rice, LR refers to the (late rice) with the heading stage synchronized to that of the RR. Data are presented as n=3 independent replicates, values with a column followed by different letters are significantly different by Duncan’s test (*p* < 0.05).

Supplementary table 8. The NSC (Non-structural Carbohydrates) transport from RR, MC, and LR during the same period in 2022.

| Rice cultivation model | Variety | NSC transport amount g plant^-1^ | | | | NSC transfer rate% | | | | | NSC contribution rate% | | | | |  |
| --- | --- | --- | --- | --- | --- | --- | --- | --- | --- | --- | --- | --- | --- | --- | --- | --- |
|  |  | root | stem | leaf | stubble | | root | stem | leaf | stubble | | root | stem | leaf | stubble | |
| MC | YY1540 | 2.08ab | 3.60c | 3.16a |  | | 42.19c | 29.65d | 45.73d |  | | 3.55c | 6.14cd | 5.39c |  | |
|  | YY1526 | 2.30a | 2.92d | 3.05a |  | | 46.37c | 31.18d | 45.45d |  | | 4.10bc | 6.99c | 5.44c |  | |
|  | TFY3301 | 1.97ab | 2.66d | 2.69c |  | | 41.64c | 26.54d | 40.39d |  | | 3.94bc | 5.33d | 5.39c |  | |
|  | HHZ | 2.13a | 2.73d | 3.24a |  | | 45.90c | 27.91d | 51.42c |  | | 4.12bc | 5.29d | 6.28b |  | |
| RR | YY1540 | 2.01ab | 4.81ab | 3.26a | 0.46a | | 61.65b | 61.04b | 77.43a | 66.67a | | 4.40b | 10.53b | 7.14a | 1.01a | |
|  | YY1526 | 2.17a | 5.13a | 2.90b | 0.44a | | 67.18a | 63.49b | 71.60b | 66.66a | | 5.01a | 11.83a | 6.69ab | 1.01a | |
|  | TFY3301 | 1.86b | 4.92a | 2.73c | 0.39b | | 60.58b | 68.71a | 75.83a | 60.93b | | 4.78a | 12.65a | 7.02a | 1.00a | |
|  | HHZ | 1.96ab | 4.68b | 2.65c | 0.45a | | 64.90ab | 66.42a | 73.81b | 68.18a | | 4.97a | 11.84a | 6.71ab | 1.14a | |
| LR | YY1540 | 1.95ab | 3.47c | 2.72c |  | | 39.15c | 31.57e | 40.96 |  | | 3.28c | 5.84d | 4.58d |  | |
|  | YY1526 | 1.34c | 4.35b | 2.90b |  | | 29.71d | 40.27c | 45.88d |  | | 2.34de | 7.36c | 5.08c |  | |
|  | TFY3301 | 1.09cd | 2.61d | 3.43a |  | | 25.11d | 27.53d | 53.93c |  | | 2.11e | 5.07e | 6.66ab |  | |
|  | HHZ | 1.25c | 2.61d | 3.36a |  | | 29.55d | 28.21d | 55.90c |  | | 2.69d | 5.63d | 7.25a |  | |

Note: In YY1540, YY1526, TFY3301, and HHZ varieties, MC refers to the main crop rice, RR refers to the ratoon season rice, LR refers to the (late rice) with the heading stage synchronized to that of the RR. Data are presented as n=3 independent replicates, values with a column followed by different letters are significantly different by Duncan’s test (*p* < 0.05).


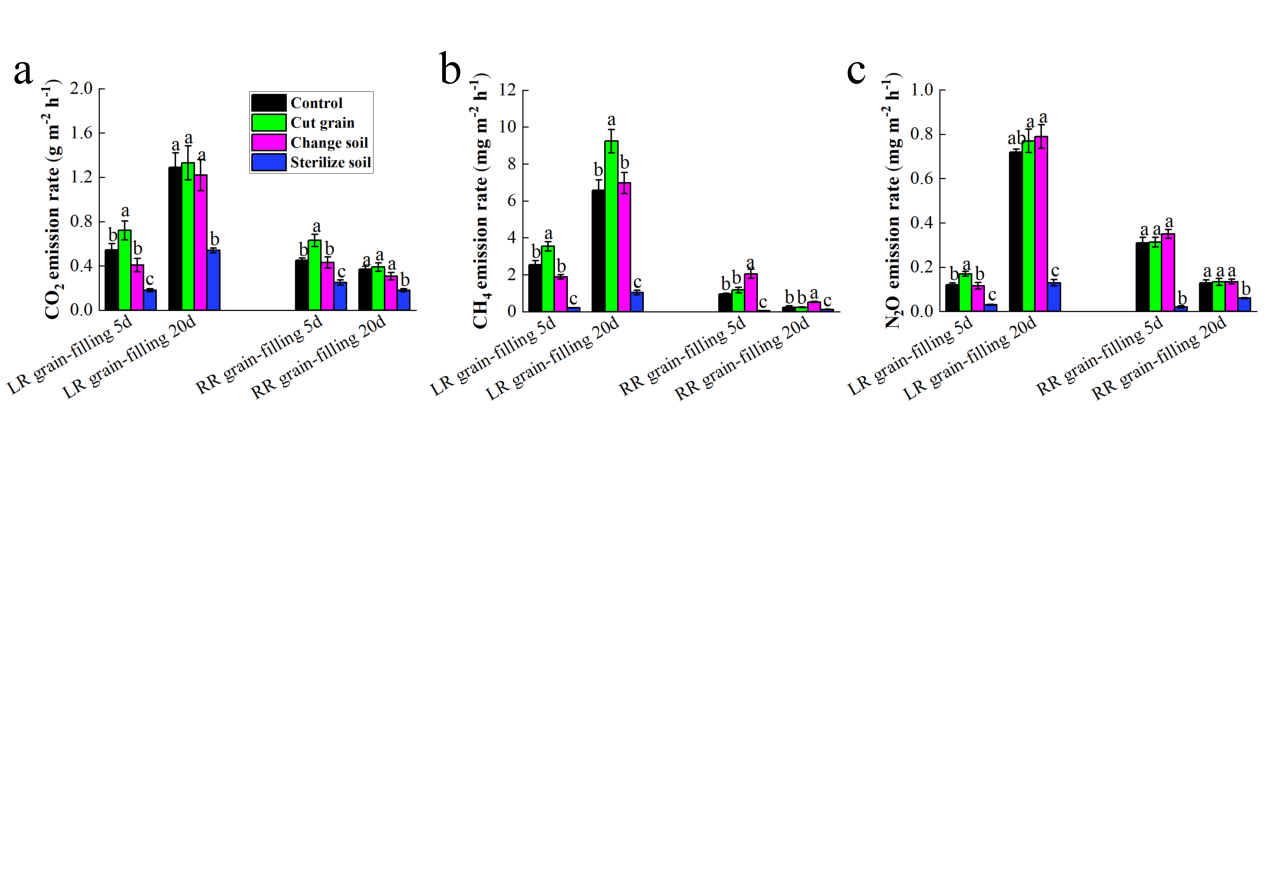


Supplementary Figure 19. Greenhouse gas emissions from defoliation, sterilization, and soil exchange of RR, MC, and LR during the same period. a) CO_2_ emissions from defoliation, sterilization, and soil exchange for different varieties of MC, RR, and LR; Means ± SD. (n = 3 biological replicates), the different lowercase letters above the column graphs indicate significant differences by Duncan’s test (*p* < 0.05) and figures were plotted using Origin 2021 software. b) CH_4_ emissions from defoliation, sterilization, and soil exchange for different varieties of MC, RR, and LR; Means ± SD. (n = 3 biological replicates), the different lowercase letters above the column graphs indicate significant differences by Duncan’s test (*p* < 0.05) and figures were plotted using Origin 2021 software. c) N_2_O emissions from defoliation, sterilization, and soil exchange for different varieties of MC, RR, and LR; Means ± SD. (n = 3 biological replicates), the different lowercase letters above the column graphs indicate significant differences by Duncan’s test (*p* < 0.05) and figures were plotted using Origin 2021 software. Note: MC refers to main crop rice, RR refers to ratoon season rice, and LR refers to synchronized in heading with the RR. Source data are provided in the source data file.


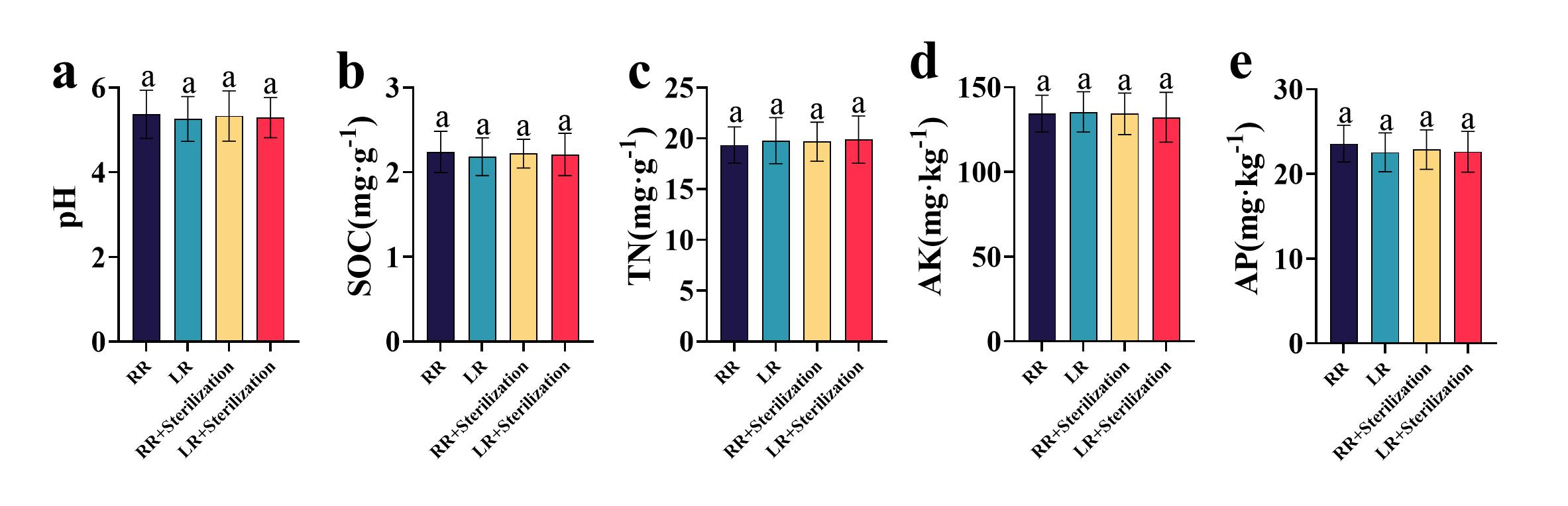


Supplementary Figure 20. Effects of rhizosphere soil sterilization on soil physical and chemical properties in YY1540. a) pH values for LR and RR control and sterilization; Means ± SD. (n = 3 biological replicates), the different lowercase letters above the column graphs indicate significant differences by Duncan’s test (*p* < 0.05) and figures were plotted using Origin 2021 software. b) Organic carbon (SOC) content for LR and RR control and sterilization; Means ± SD. (n = 3 biological replicates), the different lowercase letters above the column graphs indicate significant differences by Duncan’s test (*p* < 0.05) and figures were plotted using Origin 2021 software. c) Total nitrogen (TN) content for LR and RR control and sterilization; Means ± SD. (n = 3 biological replicates), the different lowercase letters above the column graphs indicate significant differences by Duncan’s test (*p* < 0.05) and figures were plotted using Origin 2021 software. d) Available potassium (AK) content for LR and RR control and sterilization; Means ± SD. (n = 3 biological replicates), the different lowercase letters above the column graphs indicate significant differences by Duncan’s test (*p* < 0.05) and figures were plotted using Origin 2021 software. e) Available potassium content for LR and RR control and sterilization; Means ± SD. (n = 3 biological replicates), the different lowercase letters above the column graphs indicate significant differences by Duncan’s test (*p* < 0.05) and figures were plotted using Origin 2021 software. Source data are provided in the source data file.


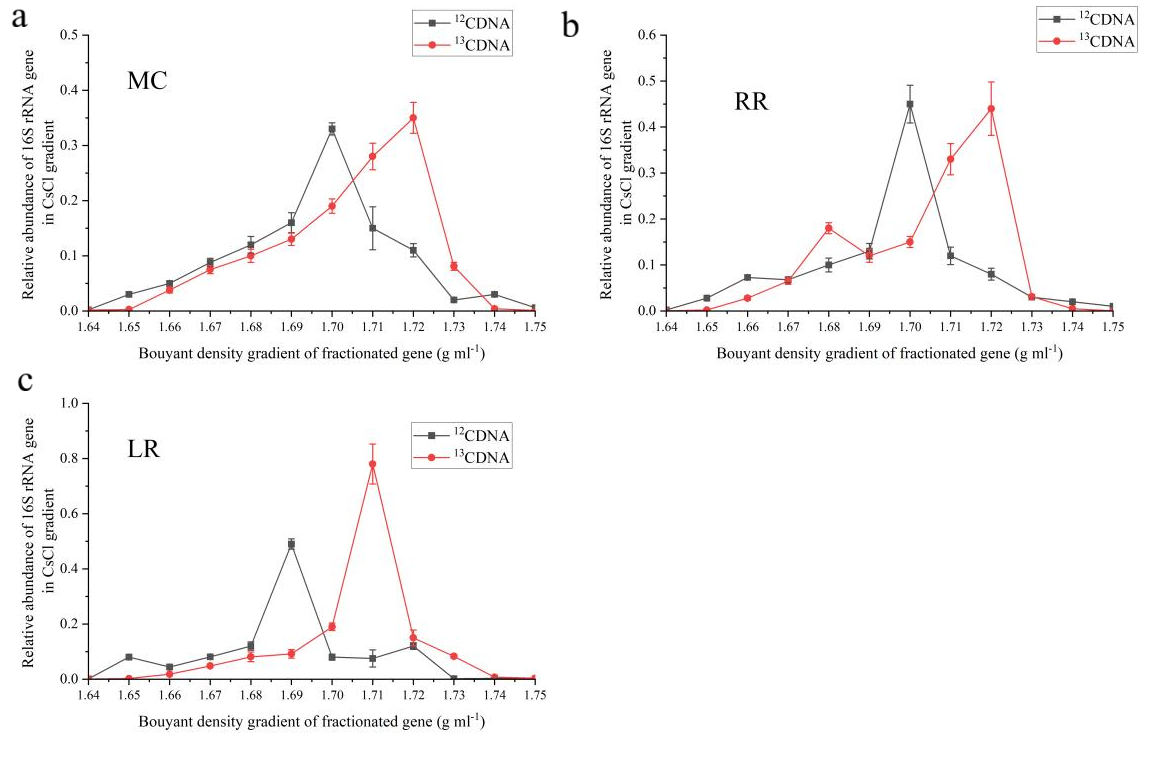


Supplementary Figure 21. Quantitative distribution of 16SrRNA Gene in the whole buoyancy density gradient of ^12^C (unlabeled) and ^13^C (labeled) rhizosphere soil DNA of rice plants at the main crop rice (MC), ratoon season rice (RR) and late rice (LR) by density analysis. a) Quantitative distribution of 16SrRNA Gene in the whole buoyancy density gradient of ^12^C (unlabeled) and ^13^C (labeled) rhizosphere soil DNA of rice plants at the main crop rice (MC) by density analysis; Means ± SD. (n = 3 biological replicates), figures were plotted using Origin 2021 software. b) Quantitative distribution of 16SrRNA Gene in the whole buoyancy density gradient of ^12^C (unlabeled) and ^13^C (labeled) rhizosphere soil DNA of rice plants at the ratoon season rice (RR) by density analysis; Means ± SD. (n = 3 biological replicates), figures were plotted using Origin 2021 software. c)Quantitative distribution of 16SrRNA Gene in the whole buoyancy density gradient of ^12^C (unlabeled) and ^13^C (labeled) rhizosphere soil DNA of rice plants at the late rice (LR) by density analysis; Means ± SD. (n = 3 biological replicates), figures were plotted using Origin 2021 software. Source data are provided in the source data file.


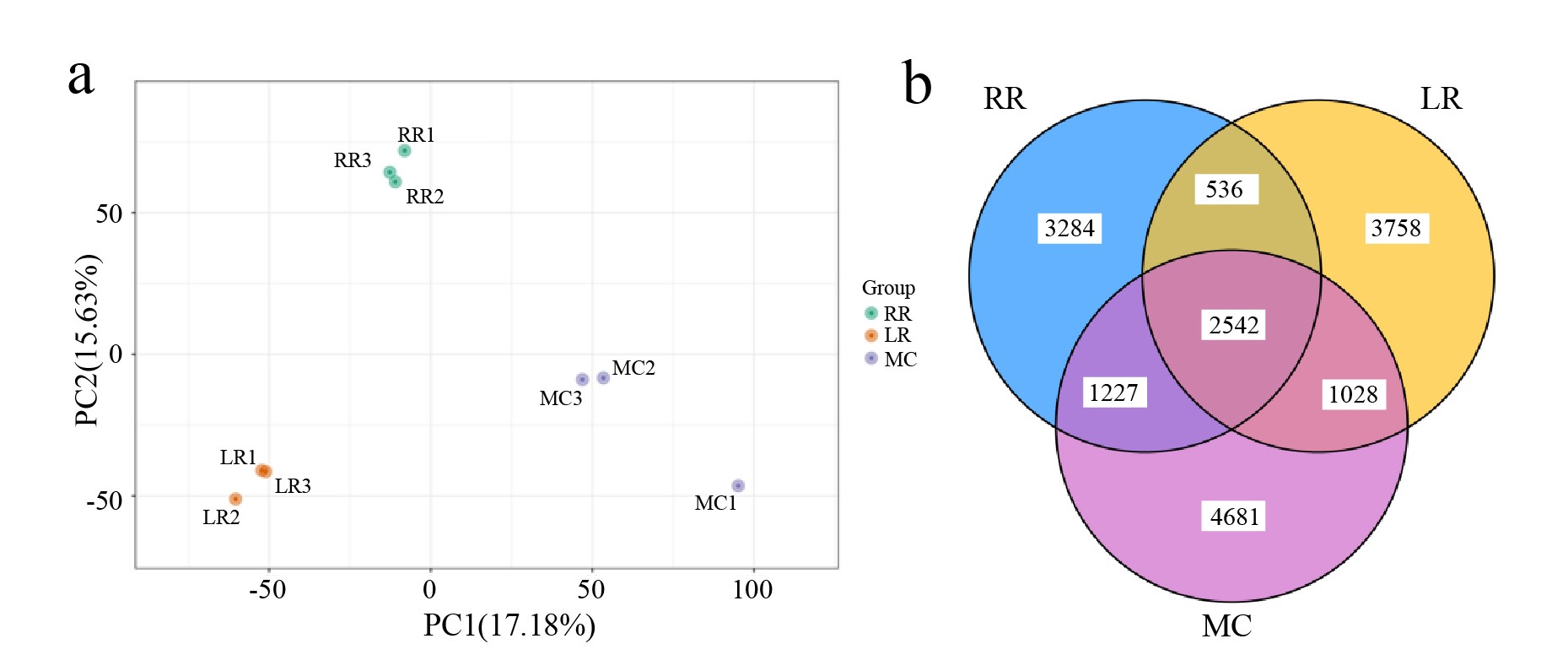


Supplementary Figure 22. Changes and Ven plots of microbial PCA under different Planting Patterns. a) PCA figure; b) Venn figure. Note: MC refers to YY1540 main crop rice, RR refers to YY1540 ratoon season rice, and LR refers to YY1540 (late season) synchronized in rice heading time. Graphics are all created using R software version 4.4.1. Source data are provided in the source data file.


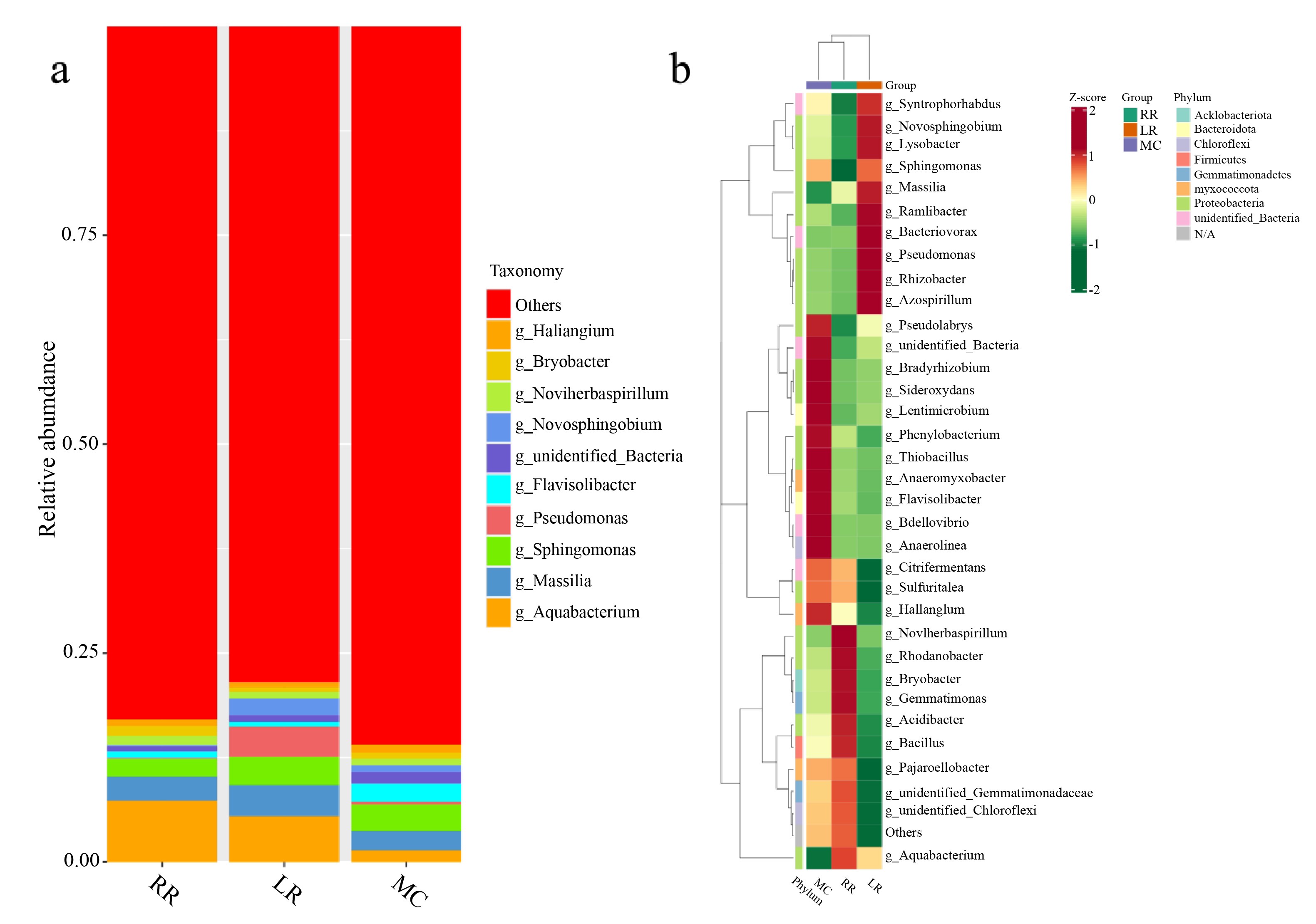


Supplementary Figure 23. Top ten relative abundances and top 35 heatmaps of microbial genus at the level of planting patterns. a) Top ten relative abundances of microbial genus at the level of planting patterns; b) Top 35 heatmaps of microbial genera at the level of planting patterns. Graphics are all created using R software version 4.4.1. Source data are provided in the source data file.


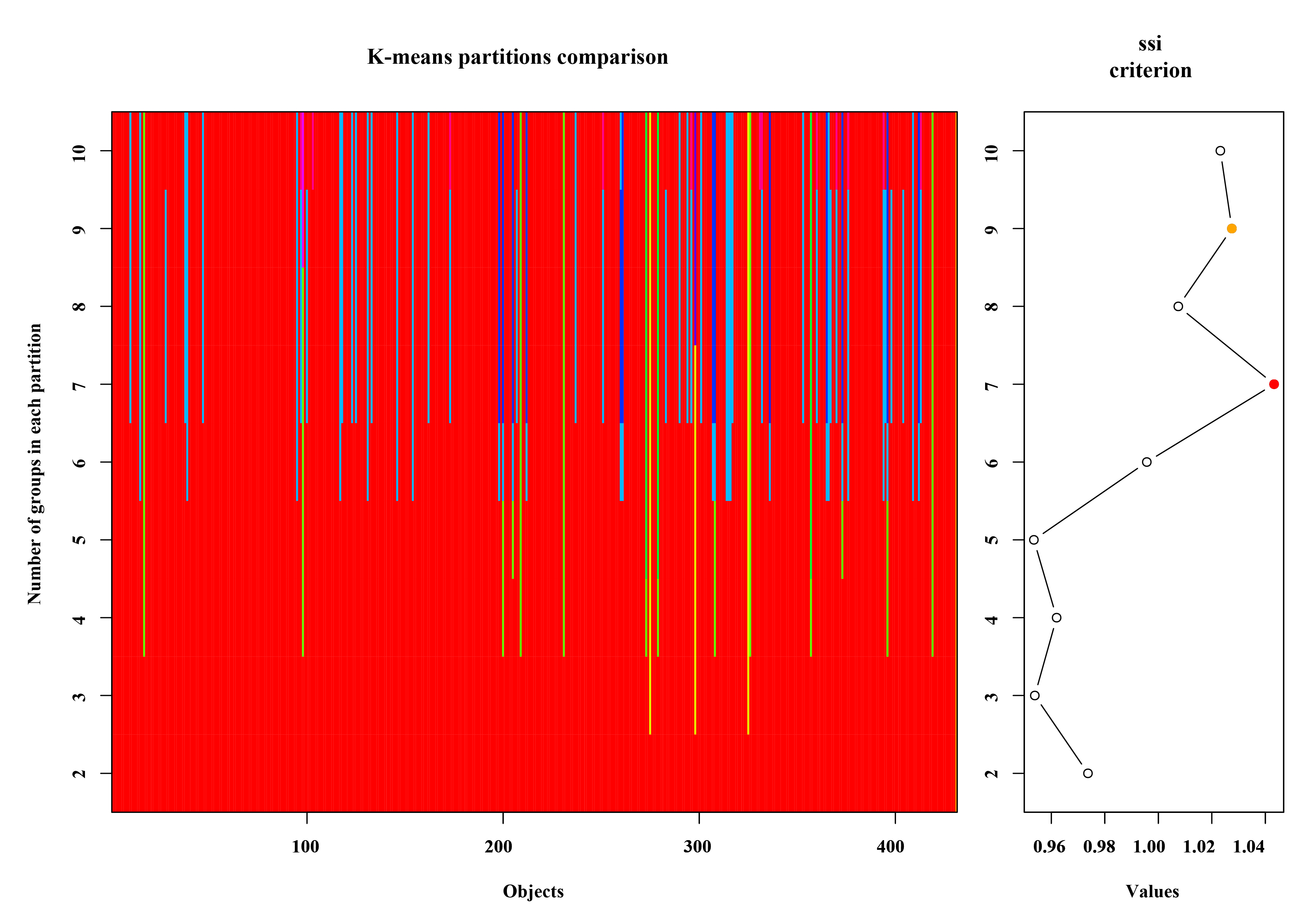


Supplementary Figure 24.K-means clustering analysis of microorganisms planting patterns. Graphics are all created using R software version 4.4.1. Source data are provided in the source data file.


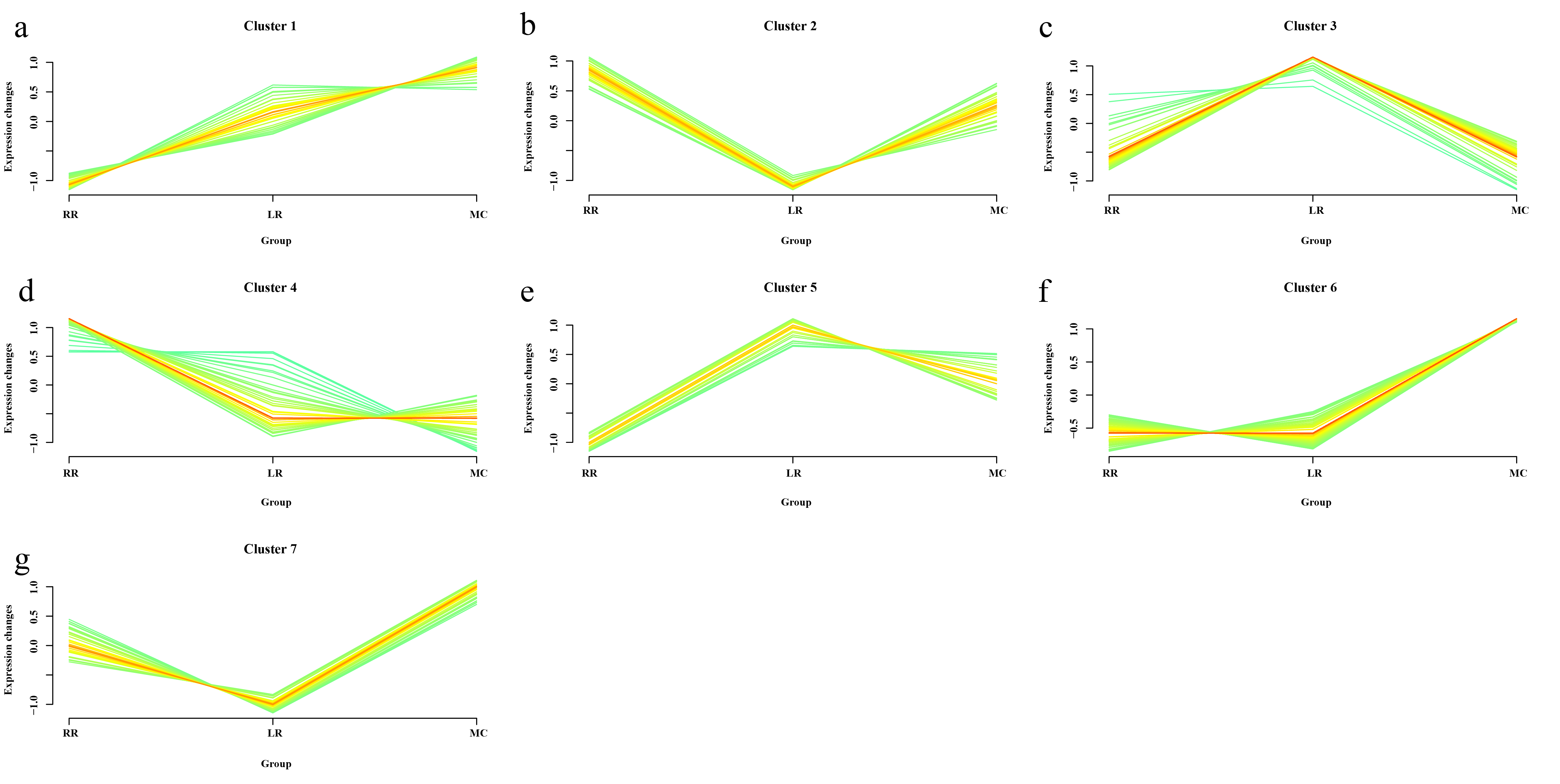


Supplementary Figure 25. Analysis of seven K-means clustering differences of microorganisms planting patterns. a) Cluster1 in RR, LR and MC; b) Cluster2 in RR, LR and MC; c) Cluster3 in RR, LR and MC; d) Cluster4 in RR, LR and MC; e) Cluster5 in RR, LR and MC; f) Cluster6 in RR, LR and MC; g) Cluster7 in RR, LR and MC. Graphics are all created using R software version 4.4.1. Note: MC refers to YY1540 main crop rice, RR refers to YY1540 ratoon season rice, and LR refers to YY1540 (late season) synchronized in rice heading time. Source data are provided in the source data file.


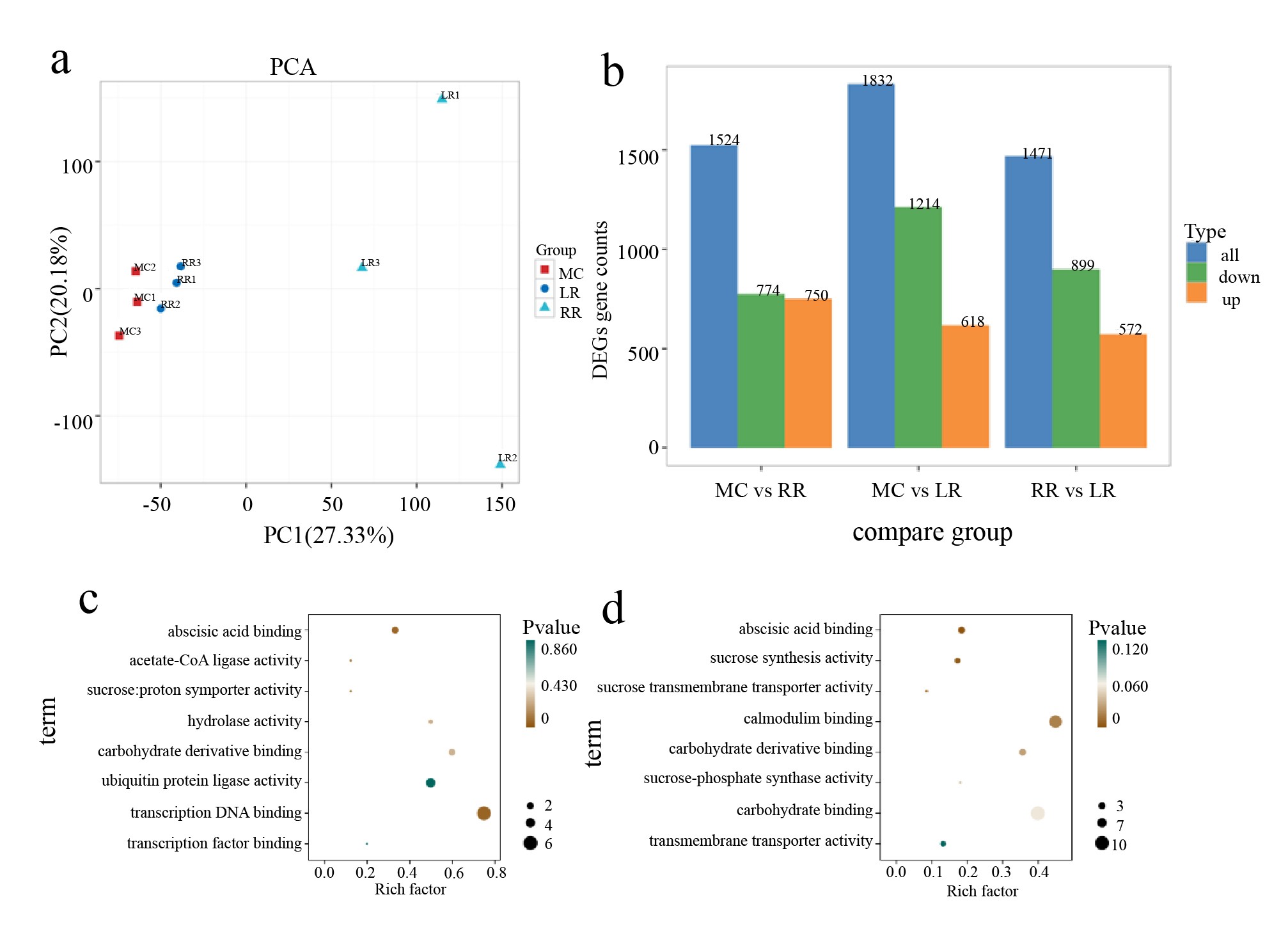


Supplementary Figure 26. Differential pathway analysis of the transcriptome genes. A) PCA figure; b) DEG figure; c) Differential pathway analysis between MC and RR; d) Differential pathway analysis between LR and RR. Graphics are all created using R software version 4.4.1. Source data are provided in the source data file.


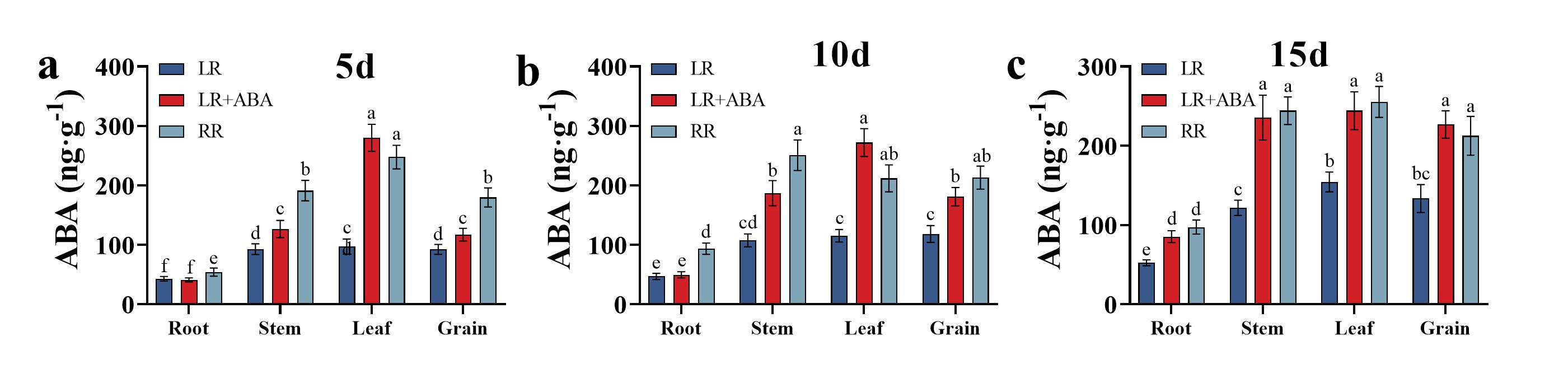


Supplementary Figure 27. Comparison of ABA content and RR in different organs on the 5th, 10th and 15th days after LR spraying ABA. a) ABA content on the 5th day; Means ± SD. (n = 3 biological replicates), the different lowercase letters above the column graphs indicate significant differences by Duncan’s test (*p* < 0.05) and figures were plotted using Origin 2021 software. b) ABA content on the 10th day; Means ± SD. (n = 3 biological replicates), the different lowercase letters above the column graphs indicate significant differences by Duncan’s test (*p* < 0.05) and figures were plotted using Origin 2021 software. c)ABA content on the 15th day; Means ± SD. (n = 3 biological replicates), the different lowercase letters above the column graphs indicate significant differences by Duncan’s test (*p* < 0.05) and figures were plotted using Origin 2021 software. Source data are provided in the source data file.

Supplementary table 9. Effect of ABA application on NSC transport in RR and LR at the same heading stage.

|  | NSC transport amount g plant^-1^ | | | NSC transfer rate% | | | | NSC contribution rate% | | | |
| --- | --- | --- | --- | --- | --- | --- | --- | --- | --- | --- | --- |
| Treatment | root | stem | leaf | | root | stem | leaf | | root | stem | leaf |
| RR+ABA | 0.84c | 6.04b | 1.01c | | 81.5a | 76.1a | 63.1a | | 2.60c | 18.7a | 3.13a |
| RR | 0.82c | 5.97b | 0.99c | | 79.9a | 75.4a | 61.9a | | 2.55c | 18.5a | 3.08a |
| LR+ABA | 1.72a | 6.96a | 1.44a | | 67.7b | 55.8b | 61.1a | | 3.20a | 12.9b | 2.68b |
| LR | 1.57b | 5.88b | 1.18b | | 61.8c | 47.2c | 50.0b | | 3.06b | 11.4c | 2.30c |

Note: RR+ABA refers to ABA spray on RR at heading stage, RR refers to ratoon season rice; LR+ABA refers to ABA spray on LR at heading stage, LR refers to the (late rice) with the heading stage synchronized to that of the RR. Data are presented as n=3 independent replicates, values with a column followed by different letters are significantly different by Duncan’s test (*p* < 0.05).


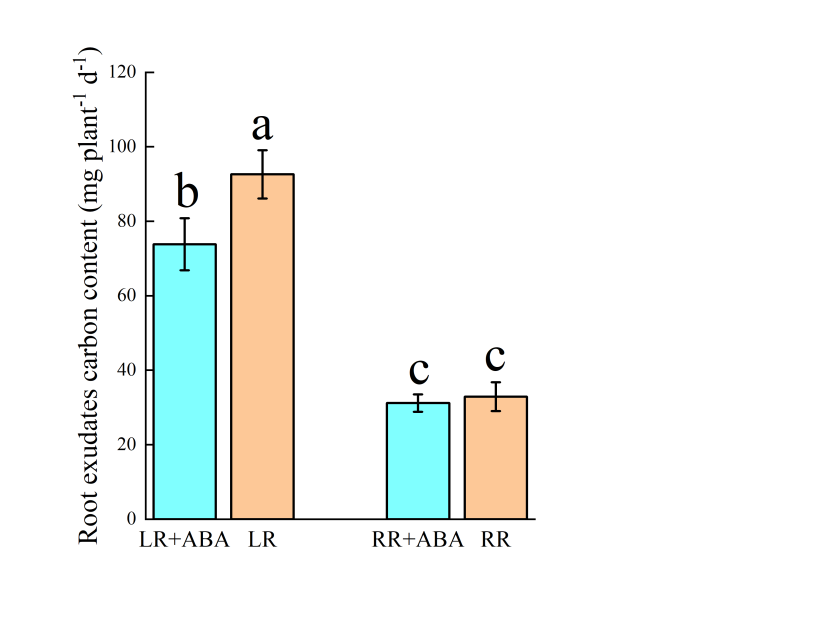


Supplementary Figure 28. Effects of ABA application on root exudates of plants. Note: LR refers to YY1540 (late season) synchronized in rice heading time. LR+ABA refers to ABA application YY1540 (late season) synchronized in rice heading time. RR refers to YY1540 ratoon season rice. RR+ABA refers to ABA application YY1540 ratoon season rice. Means ± SD. (n = 3 biological replicates), the different lowercase letters above the column graphs indicate significant differences by Duncan’s test (*p* < 0.05) and figures were plotted using Origin 2021 software. Source data are provided in the source data file.

Supplementary table 10. Effect of ABA application on yield of ratoon rice and late rice at the same heading stage.

| Treatment | Panicles m^-2^ | Spikelets per panicle | Grain filling rate(%) | 1000-grain weight  (g) | Grain yield  (t ha^−1^) | Harvest  index |
| --- | --- | --- | --- | --- | --- | --- |
| RR+ABA | 418.24a | 105.27 b | 88.36a | 23.41a | 6.45 c | 58.28 a |
| RR | 418.24 a | 105.46 b | 88.33 a | 23.38 a | 6.42 c | 57.93 a |
| LR+ABA | 259.37b | 263.85 a | 82.71 b | 23.02 a | 10.72 a | 56.31 ab |
| LR | 259.37 b | 263.72 a | 78.34 c | 22.55 ab | 10.25 b | 53.43 c |

Note: RR+ABA refers to ABA spray on ratoon season rice at heading stage; RR refers to ratoon season rice; LR+ABA refers to ABA spray on late rice at heading stage, LR refers to the (late rice) with the heading stage synchronized to that of the RR. Data are presented as n=3 independent replicates, values with a column followed by different letters are significantly different by Duncan’s test (*p* < 0.05).


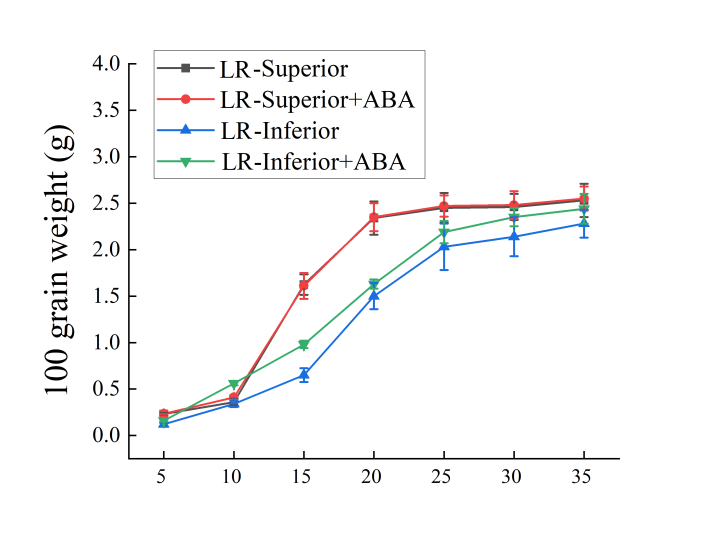


Supplementary Figure 29. Changes in hundred-grain weight after ABA application. Means ± SD. (n = 3 biological replicates), figures were plotted using Origin 2021 software. Source data provided in the source data file.

Supplementary table 11. Effect of wild-type ZH11, OsCIPK2 overexpression, and OsCIPK2 mutant on NSC transport.

| Treatment | NSC transport amount g plant^-1^ | | | | | NSC transfer rate% | | | | NSC contribution rate% | | |
| --- | --- | --- | --- | --- | --- | --- | --- | --- | --- | --- | --- | --- |
|  | root | stem | | leaf | root | | stem | | leaf | root | stem | leaf |
| ZH11 | 0.65b | 3.42b | 0.61ab | | | 52.3b | | 54.2b | 59.6ab | 4.16b | 21.89a | 3.90a |
| OE-OsCIPK2 | 0.84a | 3.94a | 0.63a | | | 61.8a | | 64.3a | 63.1a | 4.83a | 22.65a | 3.62b |
| KO-OsCIPK2 | 0.54c | 3.12c | 0.58b | | | 48.6c | | 49.5c | 52.7b | 3.78bc | 21.84a | 4.06a |

Note: ZH11 refers to the wild-type ZH11, OE-OsCIPK2 refers to the overexpression of OsCIPK2, KO-OsCIPK2 refers to the OsCIPK2 mutant. Data are presented as n=3 independent replicates, values with a column followed by different letters are significantly different by Duncan’s test (*p* < 0.05).

Supplementary table 12. Effect of wild-type ZH11, OsCIPK2 overexpression, and OsCIPK2 ^13^C mutant on content.

| Treatment | soil | root | stem | leaf | grain |
| --- | --- | --- | --- | --- | --- |
| ZH11 | 19.82b | 31.64b | 86.27b | 51.85b | 185.45b |
| OE-OsCIPK2 | 13.06c | 26.23c | 67.28c | 46.28c | 227.63a |
| KO-OsCIPK2 | 23.86a | 40.87a | 102.39a | 58.42a | 152.73c |

Note: ZH11 refers to the wild-type ZH11, OE-OsCIPK2 refers to the overexpression of OsCIPK2, KO-OsCIPK2 refers to the OsCIPK2 mutant. Data are presented as n=3 independent replicates, values with a column followed by different letters are significantly different by Duncan’s test (*p* < 0.05).


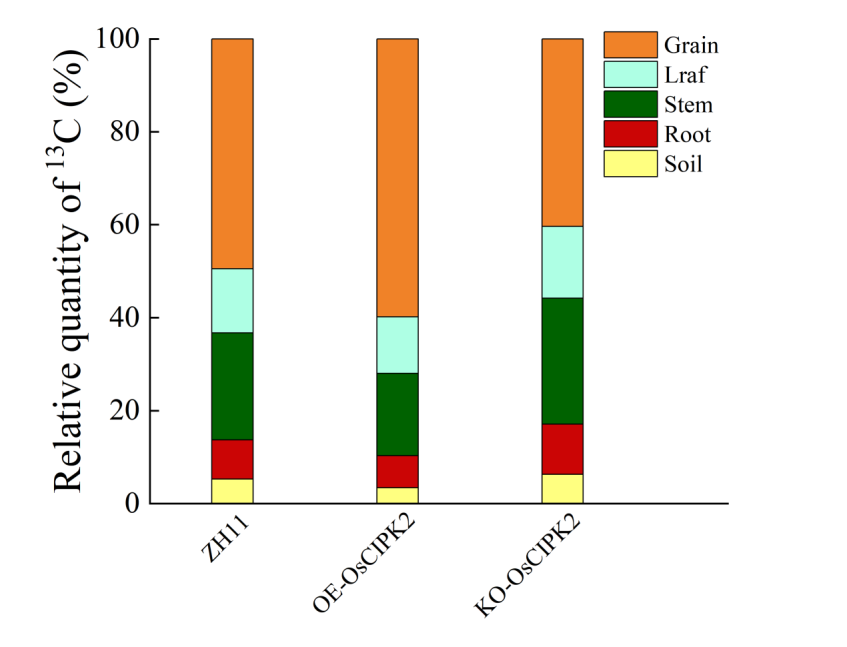


Supplementary Figure 30. Effects of wild-type ZH11, overexpressed OsCIPK2, and the mutant OsCIPK2 ^13^C on distribution rates. ZH11 refers to the wild-type ZH11, OE-OsCIPK2 refers to the overexpression of OsCIPK2, KO-OsCIPK2 refers to the OsCIPK2 mutant. Data are presented as n=3 independent replicates and figures were plotted using Origin 2021 software. Source data are provided in the source data file.

Supplementary table 13. Effects of wild-type ZH11, overexpression of OsCIPK2, and the mutant OsCIPK2 on yield.

| Variety | Panicles per plant | Spikelets per panicle | Grain filling rate (%) | 1000-grain  weight (g) | Grain yield  Per plant(g) | Harvest  index |
| --- | --- | --- | --- | --- | --- | --- |
| ZH11 | 35.25a | 62.43a | 82.43b | 22.38b | 15.62b | 48.32b |
| OE-OsCIPK2 | 36.75a | 62.95a | 89.67a | 24.57a | 17.39a | 52.69a |
| KO-OsCIPK2 | 34.97a | 62.58a | 75.34c | 20.25c | 14.28c | 44.27c |

Note: ZH11 refers to the wild-type ZH11, OE-OsCIPK2 refers to the overexpression of OsCIPK2, KO-OsCIPK2 refers to the OsCIPK2 mutant. Data are presented as n=3 independent replicates, values with a column followed by different letters are significantly different by Duncan’s test (*p* < 0.05). Source data are provided in the source data file.


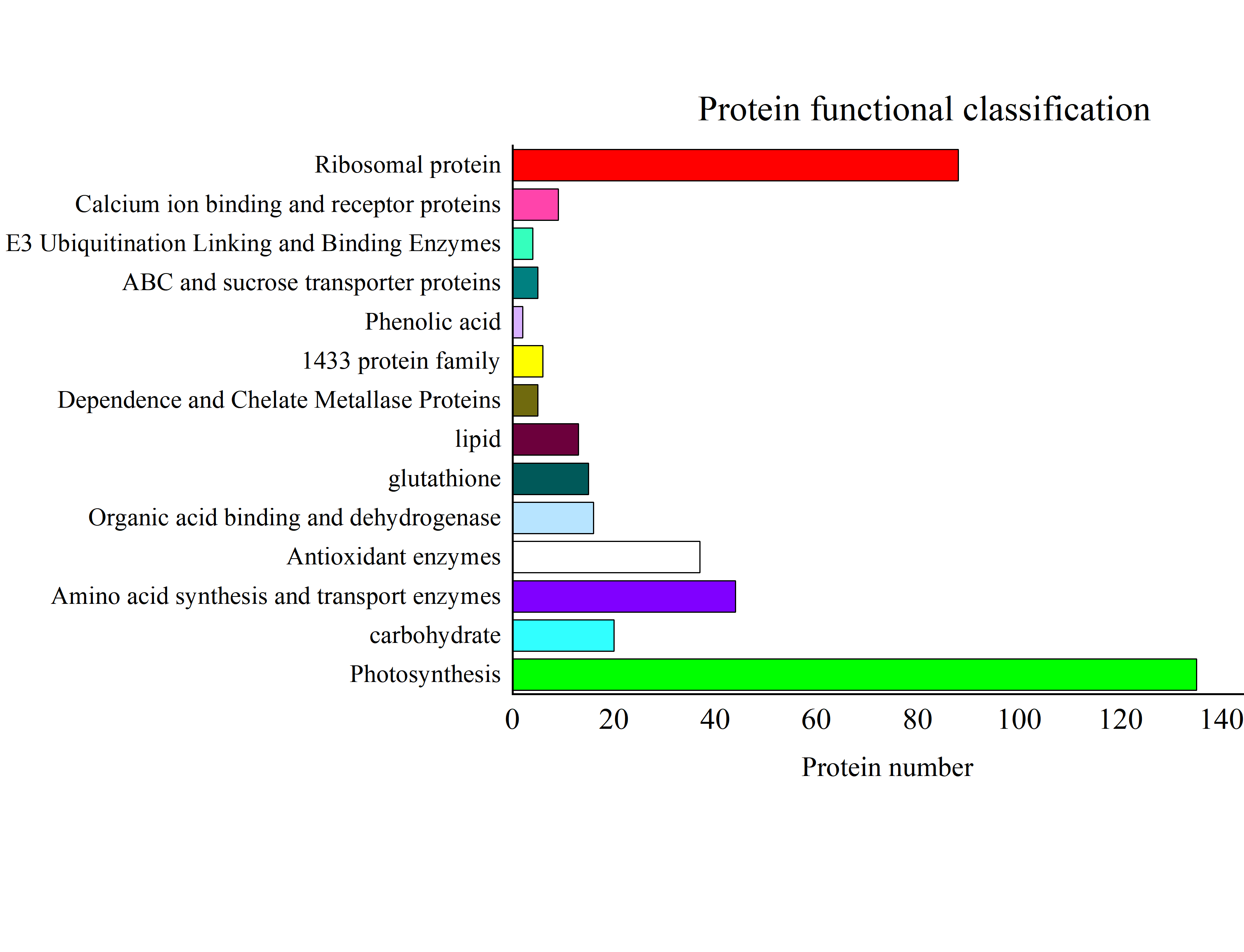


Supplementary Figure 31. Analysis of the OsCIPK2-interacting protein KEGG pathway. Graphics are all created using R software version 4.4.1. Source data are provided in the source data file.

Supplementary table 14. Effects of overexpression of OsSWEET1A on the transport of non-structural carbohydrates (NSC)

| Variety | NSC transport amount g plant^-1^ | | | | NSC transfer rate% | | | | | NSC contribution rate% | | | | |
| --- | --- | --- | --- | --- | --- | --- | --- | --- | --- | --- | --- | --- | --- | --- |
|  | root | stem | | leaf | | root | stem | leaf | | root | | stem | | leaf |
| ZH11 | 0.65b | 3.52b | 0.61b | | 52.3b | | 54.2b | | 52.6b | 4.16a | 22.53a | | 3.90a | |
| OE-OsSWEET1A | 0.69a | 3.81a | 0.69a | | 55.4a | | 59.3a | | 58.4a | 4.12a | 22.78a | | 4.12a | |

Note: ZH11 refers to wild-type ZH11, OE-OsSWEET1A refers to overexpression OsSWEET1A. Data are presented as n=3 independent replicates, values with a column followed by different letters are significantly different by Duncan’s test (*p* < 0.05).

Supplementary table 15. Effects of overexpression of OsSWEET1A on yield.

| Variety | Panicles per plant | Spikelets per panicle | Grain filling rate (%) | 1000-grain  weight (g) | Grain yield  Per plant(g) | Harvest  index |
| --- | --- | --- | --- | --- | --- | --- |
| ZH11 | 35.25a | 62.43a | 82.43b | 22.38b | 15.62b | 48.32ab |
| OE-OsSWEET1A | 35.47a | 62.29a | 87.29a | 23.82a | 16.72a | 50.24a |

Note: ZH11 refers to wild-type ZH11, OE-OsSWEET1A refers to overexpression OsSWEET1A. Data are presented as n=3 independent replicates, values with a column followed by different letters are significantly different by Duncan’s test (*p* < 0.05).
